# Real-world evidence on RSV vaccine uptake, effectiveness, and safety in older adults: a systematic review and meta-analysis

**DOI:** 10.1016/j.lanepe.2026.101623

**Published:** 2026-02-20

**Authors:** Daira Trusinska, Bohee Lee, Sohail Ferdous, Louise Lansbury, Cedric Burden, Atul Anand, Julia Stowe, Anna Mensah, WeiShen LIM, Kimberly Marsh, Cheryl Gibbons, Ting Shi

**Affiliations:** a Usher Institute, University of Edinburgh, Edinburgh, UK; b National Heart & Lung Institute, Imperial College London, London, UK; c School of Medicine, University of Nottingham, UK; d Swansea University, UK; e Centre for Cardiovascular Sciences, Institute for Neuroscience and Cardiovascular Research, University of Edinburgh, Edinburgh, UK; f Immunisation & Vaccine Preventable Diseases, UK Health Security Agency, UK; g Public Health Scotland, Glasgow, UK

**Keywords:** Respiratory syncytial virus (RSV), RSV vaccines for older adults, RSV vaccine uptake, RSV vaccine effectiveness, RSV vaccine safety

## Abstract

**Background:**

Vaccines to prevent respiratory syncytial virus (RSV)–associated lower respiratory tract disease in older adults have become available in recent years. We investigated RSV vaccine uptake, effectiveness, and safety signals in older adults reported in post-licensure real-world studies.

**Methods:**

For this systematic review and meta-analysis, we conducted 11 monthly searches (between November 5, 2024, and November 10, 2025) in Ovid Medline, Embase, and Global Health databases. Meta-analyses, using random-effects modelling, were performed for uptake, effectiveness, and safety signals. PROSPERO registration: CRD42025643585.

**Findings:**

A total of 3900 studies were identified, of which 36 were included, published between December 22, 2023, and October 28, 2025, and covering over 121.8 million individuals across the United States, United Kingdom, Italy, Australia, Czech Republic, Switzerland, France, Canada, and Israel. In the United States, RSV vaccine uptake among adults aged ≥60 years during the 2023/24 RSV season was 18.0% (95% confidence interval (CI): 12.2–25.7; ten studies), varying by clinical and socio-demographic subgroups. Among adults aged ≥60 years, pooled estimates of vaccine effectiveness were 75.3% (95% CI: 73.7–76.9; three studies) against any laboratory-confirmed RSV-positive infection, 76.4% (95% CI: 74.2–78.5; four studies) against RSV-related emergency department or urgent care visits, 74.8% (95% CI: 66.8–82.9; six studies) against RSV-related hospital admissions, and 79.8% (95% CI: 68.1–91.5; four studies) against severe RSV-associated disease. Following vaccination, Guillain-Barré syndrome (GBS) was reported in two studies with between 5.2 and 6.5 cases per one million doses for RSVPreF3+AS01 (*Arexvy*, GSK) vaccines and between 9.0 and 18.2 cases per one million doses for RSVpreF (*Abrysvo*, Pfizer) vaccines.

**Interpretation:**

RSV vaccine uptake in older adults was low globally with substantial disparities between sociodemographic and clinical subgroups. Our study showed a favourable safety profile and high effectiveness of the RSV vaccines, highlighting the value of wide implementation of these vaccines.

**Funding:**

There was no funding for the study.

**Research in context**

**Evidence before this study**

Respiratory syncytial virus (RSV) is a substantial health threat to adults aged 60 years and older. RSV-associated acute respiratory infections (ARI) lead to substantial healthcare utilisation, including hospital admissions, as well as cause functional decline, frailty, disability, and mortality in this age group. To prevent severe RSV-associated disease, three new RSV vaccine products have been approved and introduced in countries across Europe, North America, Asia, and Australia, with more countries preparing to implement RSV immunisation programmes. With the countries in the Northern Hemisphere in the 2025/26 RSV season, it is critical to closely monitor vaccine uptake and early reports of effectiveness and safety in real-world settings to adjust vaccination programmes and increase public confidence in the vaccines. To explore existing literature, we used search terms such as “respiratory syncytial virus”, “vaccines” and “systematic review” in PubMed in October 2024. To our knowledge, this is the first systematic review and meta-analysis to summarise geographically broad real-world data on RSV vaccines in older adults, as previously studies only reported data from the United States.

**Added value of this study**

Up to November 10, 2025, real-world uptake, effectiveness, and/or safety data were reported in 36 studies from nine countries (the United States, United Kingdom, Italy, Australia, Czech Republic, Switzerland, France, Canada, and Israel) on a total of 121.8 million people. During the 2023/24 and 2024/25 RSV seasons in the Northern hemisphere, uptake of RSV vaccines among older adults was low and substantial disparities were observed by population subgroups in the United States. Our findings reflected high RSV vaccine effectiveness in preventing healthcare utilisation using different outcome metrics (ranging from 74.8% against RSV-related hospital admissions to 79.8% against RSV-related severe disease (defined as intensive care unit (ICU) admission, use of supplemental oxygen, or in-hospital death). The overall safety profile was favourable; however, a slight increase in Guillain-Barré syndrome (GBS) incidence post vaccination was seen in two independent hypothesis testing studies from the United States.

**Implications of all the available evidence**

Our findings show an up-to-date overview of the results of RSV vaccine introduction in a real-world setting, offering policy makers insights into vaccine uptake disparities, consistently high vaccine effectiveness, and highlighting areas where ongoing monitoring is essential.

## Introduction

Respiratory syncytial virus (RSV) presents a substantial disease burden in adults aged 60 years and older.^1^, ^2^, ^3^ However, studies have shown that the burden of RSV in older adults is underreported or under ascertained due to a variety of reasons including lack of healthcare attendance and lack of awareness among patients and healthcare providers.^4^ In recent population-based cohort studies from high-income countries, RSV was detected in 2.4%–4.6% of adults aged 60 years or older with acute respiratory infections (ARI).^5^, ^6^, ^7^ In 2023, an estimated 225,000 RSV-related hospital admissions and 20,000 deaths among adults aged 65 years and older occurred in the United States.^8^ Studies show that while hospital admissions associated with SARS-CoV-2 or seasonal influenza are more common than those for RSV in this age group, clinical outcomes for RSV-positive ARI patients are more severe.^9^ RSV infection requiring hospitalisation substantially deconditions older patients who survive, resulting in functional decline, loss of independence and requiring onset of new care.^10^^,^^11^

There are higher rates of RSV-associated hospital admissions with increasing age. For example, in the United Kingdom, the annual estimated RSV-associated hospitalisation rates were 71 (95% CI: 52–90) and 251 (95% CI: 186–316) per 100,000 people in age groups 65–74 years old and 75 years and older, respectively.^12^ Older individuals who are immunocompromised or who have certain comorbidities such as chronic obstructive pulmonary disease, cardiovascular disease, diabetes, and kidney disease are also at higher risk of more severe disease. A population-based surveillance study in the United States showed that hospitalisation rates due to RSV in older adults with congestive heart failure, diabetes, and chronic obstructive pulmonary disease were 4.0–33.2 times, 2.4–11.4 times, and 3.2–13.4 times higher, respectively, compared to older adults without these comorbidities.^13^ Additionally, some reports suggest that RSV infection contributes to exacerbations of underlying health conditions and may trigger cardiac events in vulnerable older adults.^14^^,^^15^

To prevent RSV-associated lower respiratory tract disease, three RSV vaccines were approved for use in adults aged 60 and older in recent years: RSVPreF3+AS01—an adjuvanted recombinant stabilized prefusion F protein vaccine (*Arexvy*, GSK); RSVpreF—a non-adjuvanted recombinant stabilized prefusion F protein vaccine (*Abrysvo*, Pfizer); and *mRESVIA* (Moderna)—an mRNA-based vaccine which encodes a stabilized prefusion F protein.^16^, ^17^, ^18^ Vaccine eligibility criteria vary by country: in the United States, it is recommended to all adults aged 75 and older and adults aged 50–74 years old who are at increased risk of severe RSV-associated disease^19^; in the United Kingdom, the vaccination programme has been rolled out to adults aged 75–79 years old only (during the 2024/25 RSV season)^20^^,^^21^; in Australia, RSV vaccines are recommended to all adults aged 75 and older and those 60–74 years old who are Aboriginal and Torres Strait Islander people or at an increased risk of severe RSV-associated disease due to comorbidities.^22^ In clinical trials, RSV vaccines showed high efficacy in older adults. Efficacy against RSV-associated lower respiratory tract disease was reported by Walsh *et al*. (2023) at 87.5% (95% CI: 58.9–97.6) for *Abrysvo* (Pfizer) vaccines and by Papi *et al*. (2023) at 84.6% (95% CI: 32.0–98.3) for *Arexvy* (GSK) vaccines in adults aged 60 years and older across one RSV season.^23^^,^^24^ Additionally, Wilson *et al*. (2023) reported an efficacy of 83.7% (95% CI: 66.0–92.2) for *mRESVIA* (Moderna) against RSV-associated lower respiratory tract infections (LRTI) and 68.4% (95% CI: 50.9–79.7) efficacy against RSV-associated acute respiratory disease.^25^ Modelling studies based on effectiveness estimates from clinical trials indicated that RSV vaccines could significantly reduce the RSV disease burden in older adults, provided that high vaccine uptake rates are achieved.^26^^,^^27^ Data from clinical trials reported generally favourable safety profiles.^24^^,^^25^ Although clinical trials had strict inclusion criteria and limited power to assess rare adverse events, for both *Arexvy* (GSK) and *Abrysvo* (Pfizer) vaccines a small excess risk of Guillain-Barré syndrome (GBS) and atrial fibrillation was reported in intervention compared to control groups.^28^ We did not observe a similar risk for *mRESVIA* (Moderna) vaccines.^25^ Studies conducted in real-world settings, when vaccines are administered to large numbers of individuals, are essential.

In this systematic review and meta-analysis, we aimed to investigate RSV vaccine uptake, vaccine effectiveness, and safety profiles in older adults, including those with comorbidities, reported in real-world post-licensure studies.

## Methods

### Search strategy and selection criteria

We carried out eleven monthly searches in Ovid Medline, Ovid Embase, and Ovid Global Health databases between November 5, 2024, and November 10, 2025. The search strategy was developed by following the Peer Review of Electronic Search Strategies (PRESS) statement.^29^ Full search strategies for each database are available in the Supplementary Materials (pp 4–5). Other relevant studies were added manually from reference lists of the included studies.

Covidence software was used to remove duplicates and complete the study screening process.^30^ Each study was independently screened by two of five reviewers (DT, SF, BL, LL, CB). We included primary studies reporting real-world evidence on RSV vaccine uptake, effectiveness, and safety in older adults and adults with comorbidities (detailed study selection criteria are provided in Supplementary Materials, p 6). Data from the included studies were extracted and quality assessments completed using a standard data extraction form. The risk-of-bias quality assessments were carried out using Joanna Briggs Institute (JBI) Critical Appraisal Tools tailored for different study designs.^31^ In line with previously published systematic reviews,^32^^,^^33^ we considered studies to be at ‘low’ risk of bias, ‘medium’ risk of bias and ‘high’ risk of bias if quality assessment scores were above 75%, 51–74%, and 50% or less, respectively. All disagreements were resolved in discussion with a third reviewer.

### Data analysis and statistics

Vaccine uptake (%) was calculated from the number of immunised individuals within an eligible population. Vaccine effectiveness was defined as the relative reduction in the odds or risk ratio of RSV-associated healthcare utilisation (severe RSV-associated disease was defined as intensive care unit (ICU) admission, use of supplemental oxygen, and/or death). Vaccine effectiveness and corresponding 95% CIs were calculated according to formula: effectiveness (%) = (1-adjusted effect ratio) x 100%. In the meta-analysis, safety signals were evaluated according to the prevalence of adverse events following vaccination (calculated from the number of adverse event reports within a vaccinated population). For GBS, the included studies reported attributable risk per one million vaccine doses (summarised descriptively). Vaccine uptake, effectiveness, and safety signals were assessed by population subgroups including age groups, ethnic/racial groups, sex, and chronic health conditions. If the same study population was reported in several studies, only the most recent data were included in the meta-analysis to avoid duplicating study populations.

Meta-analyses were carried out to provide pooled estimates when at least three studies reported: (1) population-based uptake data from the same country, (2) vaccine effectiveness against specific healthcare utilisation outcomes, or (3) data on specific adverse events post vaccination. For uptake subgroup meta-analyses, odds ratios (ORs) and 95% CIs were calculated by comparing uptake data across subgroups within each study. Meta-analyses with at least three data points were conducted using random effects modelling. Where sufficient data were available, we carried out sensitivity analyses only including data from studies classified as at ‘low risk of bias’. Study heterogeneity was assessed with I^2^ statistic: low heterogeneity (<25%), moderate heterogeneity (25–50%), high heterogeneity (>50%). Publication bias was assessed using funnel plots and Egger's test for meta-analyses including at least ten studies.^34^ All meta-analyses were completed in R software (version 4.2.3) using ‘meta’ package.

### Ethics approval

A protocol for this study was registered on PROSPERO (registration number: CRD42025643585), and the reporting of the study followed the Preferred Reporting Items for Systematic Reviews and Meta-Analyses (PRISMA) guidelines.^35^ Ethical approvals for this study were not required, as we only used published data.

### Role of the funding source

The funder was not involved in the study design, data collection, data analysis, interpretation, writing of the manuscript or decision to submit the manuscript for publication.

## Results

**Fig. 1:**
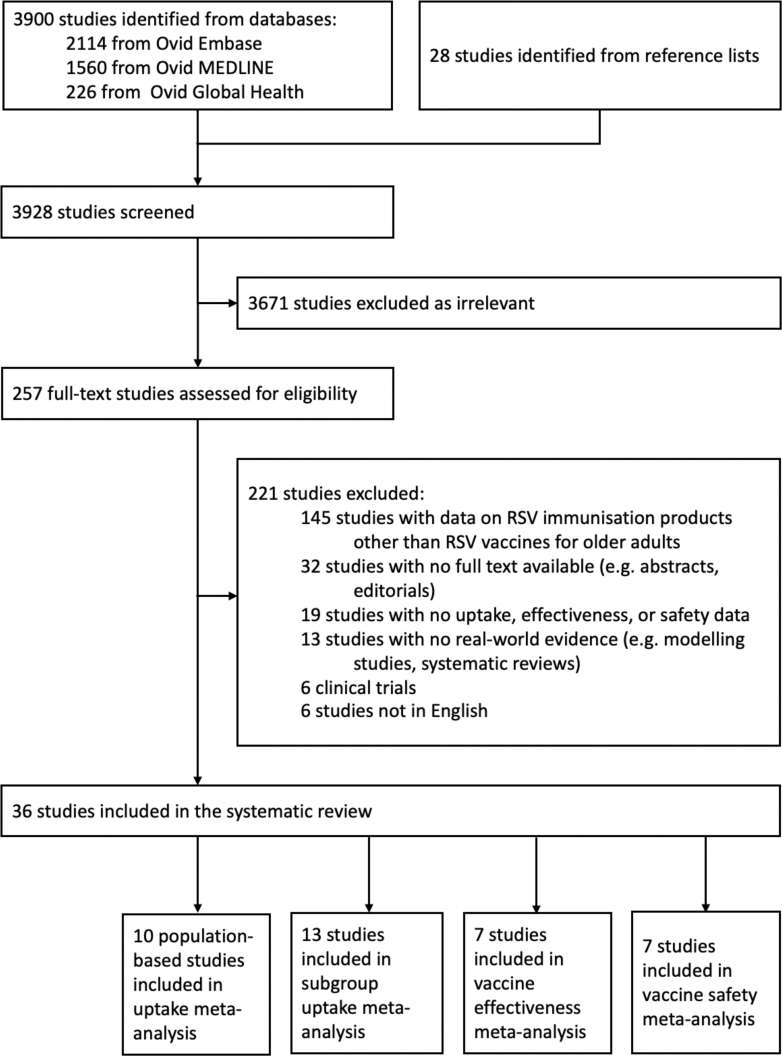
**PRISMA flow chart**.

After removing duplicates, our search strategy identified a total of 3900 studies, and another 28 potentially relevant articles were added manually from reference lists. After the initial screening, 257 full-text studies were assessed for eligibility and 221 studies excluded—exclusion reasons are shown in the PRISMA flow chart (Fig. 1) and the full list of excluded studies are available in the Supplementary Materials (pp 7–10). Thirty-six studies, published between December 22, 2023, and October 28, 2025, were included in the systematic review, encompassing data on more than 121.8 million people from studies conducted across nine countries (United States, United Kingdom, Italy, Australia, Czech Republic, Switzerland, France, Canada, and Israel). Uptake of RSV vaccines among older adults was reported in 23 studies: 19 from the United States,^9^^,^^36^, ^37^, ^38^, ^39^, ^40^, ^41^, ^42^, ^43^, ^44^, ^45^, ^46^, ^47^, ^48^, ^49^, ^50^, ^51^, ^52^, ^53^ three from the United Kingdom,^54^, ^55^, ^56^ and one from Canada.^57^ Thirteen of these were population-based studies, of which ten were included in the uptake meta-analysis (all studies from the United States for the 2023/24 RSV season). Vaccine effectiveness data were presented in nine studies: eight from the United States^37^^,^^39^^,^^44^^,^^48^^,^^50^, ^51^, ^52^^,^^58^ and one from the United Kingdom.^56^ Two studies from the United Kingdom reported population-level impact of RSV vaccines.^54^^,^^55^ Adverse events following RSV vaccination among older adults were explored in 14 studies: seven from the United States,^36^^,^^37^^,^^59^, ^60^, ^61^, ^62^, ^63^ and one from each of Canada,^64^ Italy,^65^ Australia,^66^ Czech Republic,^67^ Switzerland,^68^ France,^69^ and Israel.^70^

Of the included studies, 16 (44.4%) were cohort studies, ten (27.8%) were cross-sectional studies, six (16.7%) were test-negative case–control studies, two (5.6%) were regression discontinuity design studies, one (2.8%) study was a case series, and one (2.8%) presented data for two different study designs (case series and test-negative case–control studies). One (2.8%) study was a pre-print.^61^ In the Northern hemisphere, 22 (61.1%) studies reported real-world data from the 2023/24 RSV season, seven (19.4%) studies focused on the 2024/25 RSV season, and six (16.7%) studies reported data from both seasons. Additionally, one (2.8%) study reported data from the 2024 RSV season in the Southern hemisphere.^66^ Table 1 shows study design and population characteristics for all included studies. According to the risk-of bias assessment using JBI Critical appraisal tools, 16 (44.4%) studies were classified as at ‘low risk of bias’, 12 (33.3%) were ‘medium risk of bias’, and seven (19.4%) were ‘high risk of bias’; one (2.8%) study, which presented two study designs, was classified as at ‘low risk of bias’ (case series design) and ‘medium risk of bias’ (test-negative case–control design).^37^ Full quality assessments for each study are shown in the Supplementary Materials (pp 11–16).

**Table 1:** Characteristics of the included studies.

Study	Country	Recruitment period	Study design	Target population for vaccines	Followed study population	Study setting	Outcomes reported	Sample size	Population demographics
Bajema *et al*. (2025)	USA	Sep 1–Dec 31, 2023	Cohort study	Adults ≥60 years old	Adults ≥60 years old with RSV-positive ARI	Primary care, secondary care	VE against RSV-positive: disease, hospitalisation, ED visits	3,414,288	Median (IQR) age: 74.4 (67.8–78.6) years; 5.8% females; Ethnicity and race: 74.1% White (non-Hispanic), 16.2% Black or African American (non-Hispanic), 2.3% other, 7.4% unknown
Bao *et al*. (2025)	USA	May 3, 2023–Mar 28, 2025	Cross-sectional study	Adults ≥60 years old	Adults ≥60 years old	Community	Adverse events	7916	Median (IQR) age: 72 (66–77) years for RSVPreF3 recipients, 70 (62–77) years for RSVpreF recipients, 68 (60–76) years for mRNA-1345 recipients; 65.1% females
Birabaharan *et al*. (2024)	USA	May 3, 2023–May 3, 2024	Cohort study	Adults ≥60 years old	Adults ≥60 years old	Community	Vaccine uptake (population-based); adverse events	357,814	Mean (SD) age: 72.5 (7.1) years; 59.0% females; Ethnicity: 3.5% Hispanic or Latino, 88.0% not Hispanic or Latino, 8.5% unknown; Race: 6.1% Black or African American, 0.2% AIAN, 2.3% Asian, 0.2% NHOPI, 2.4% other, 85.3% White, 3.5% unknown
Domnich *et al*. (2025)	Italy	Feb–Sep 2024	Cohort study	Adults ≥60 years old	Adults ≥60 years old	Community	Adverse events	453	Mean (SD) age: 74.9 (8.0) years; 49.4% females; Ethnicity: 97.1% Italian, 2.9% other
Donahue *et al*. (2025)	USA	Aug 1, 2023–Sep 28, 2024	Cohort study	Adults ≥60 years old	Adults ≥60 years old	Community	Adverse events	436,823 episodes	By age group: 12.6% were 60–64 y.o., 19.9% were 65–69 y.o., 23.8% were 70–74 y.o., 21.9% were 75–79 y.o., 12.8% were 80–84 y.o., 6.1% were 85–89 y.o., 3.0% were ≥90 y.o.; 56.3% females; Ethnicity and race: 61.2% White, 10.1% Black, 12.5% Asian, 0.3% Hawaiian/Pacific Islander, 0.2% Native American/Alaskan Native, 3.5% Multiracial/Other; 9.9% Hispanic, 2.3% unknown
Fry *et al*. (2025)	USA	Oct 1, 2023–Apr 30, 2024	Test-negative case–control study	Adults ≥60 years old	Adults ≥60 years old with medically attended ARI	Primary care, secondary care	Vaccine uptake; VE against RSV-related: medical encounters, ED visits, hospitalisation	787,822	Median (IQR) age for cases: 74 (67–82) years, median (IQR) age for controls: 73 (67–81) years; Ethnicity and race: 80.7% White (non-Hispanic), 14.1% Black or African American (non-Hispanic), 2.4% Asian (non-Hispanic), 0.9% AIAN, 0.3% NHOPI, 7.8% other
(as above)	USA	Jul 1, 2023–Jun 30, 2024	Case series	Adults ≥60 years old	Adults ≥60 years old	Primary care, secondary care	Adverse events	4,746,518	Median (IQR) age: 74 (69–79) years; Ethnicity and race: 86.7% White (non-Hispanic), 6.9% Black or African American (non-Hispanic), 3.4% Asian (non-Hispanic), 0.5% AIAN, 0.3% NHOPI, 7.8% other
Geng and Wang (2024)	USA	Jan 9–Mar 4, 2024	Cross-sectional study	Adults ≥60 years old	Adults ≥60 years old	Community	Vaccine uptake (population-based)	49,322	26.7% were aged 60–64 years, 28.6% were aged 65–69 years, 22.7% were aged 70–74 years, 13.4% were aged 75–79 years, 8.6% were aged ≥80 years; 53.0% females; Ethnicity and race: 73.5% White (non-Hispanic), 10.3% Hispanic; 9.6% Black or African American (non-Hispanic), 3.5% Asian (non-Hispanic), 3.1% other or multiple races
Godonou *et al*. (2025)	USA	Aug 1, 2023–Mar 1, 2024	Cohort study	Adults ≥60 years old	Adults ≥60 years old	Community	Vaccine uptake (population-based); VE against RSV-positive: any infection, symptomatic illness	281	Median (IQR) age: 67 (60–87) years; 71.2% females; Ethnicity: 2.9% Hispanic or Latino, 95.0%, not Hispanic or Latino, 2.1% unknown; Race: 1.8% Black or African American, 1.4% multiple, 1.1% Asian, <1% Middle Eastern or North African, 93.6% White, 1.8% unknown
Hall *et al*. (2025)	Canada	Sep 19, 2024–Feb 9, 2025	Cohort study	Transplant recipients >18 years old	Transplant recipients >18 years old	Secondary care	Adverse events	86	Median (IQR) age: 64 (59–69) years for allogenic haematopoietic cell transplant recipients, 59 (46–68) years for lung transplant recipients; 41.9% females
Hameed *et al*. (2025)	Scotland, UK	Aug 12–Dec 8, 2024	Quasi-experimental (regression discontinuity design)	Adults 75–79 years old	Adults 74–79 years old with RSV-positive hospitalisation	Secondary care	Vaccine uptake (population-based), VE against RSV-associated hospitalisations	294,506	No population characteristics reported
Hause *et al*. (2024)	USA	May 3, 2023–Apr 14, 2024	Cohort study	Adults ≥60 years old	Adults ≥60 years old	Community	Adverse events	16,220	Median (IQR) age: 70 (60–94) years; 59.7% females
(as above)	USA	May 3, 2023–Apr 14, 2024	Cohort study	Adults ≥60 years old	Adults ≥60 years old	Community	Adverse events	3200	Median (IQR) age: 72 (60–112) years; 69.9% females
Havlin *et al*. (2025)	Czech Republic	Jan 9–Mar 11, 2024	Cohort study	Lung transplant recipients aged ≥60 years old	Lung transplant recipients aged ≥60 years old	NA	Adverse events	30	Median (IQR) age: 66.5 (64.0–68.0) years; 20.0% females
Kim *et al*. (2025)	USA	Sep 1, 2023–Dec 31, 2024	Cohort study	Adults ≥60 years old	Adults ≥60 years old	Community	Vaccine uptake (population based)	6,255,100	5.2% females; Race and ethnicity: 0.6% AIAN, 0.9% Asian, 17.3% Non-Hispanic Black, 5.5% Hispanic or Latino, 0.7% Native Hawaiian/Other Pacific Islander, 73.4% Non-Hispanic White, 1.6% more than one race
La *et al*. (2025)	USA	Aug 1, 2023–Feb 28, 2025	Cross-sectional study	Adults ≥60 years old	Adults ≥60 years old	Community	Vaccine uptake (population based)	77,925,739	Mean (SD) age: 71.1 (7.7) years; 55.5% females; Ethnicity and race for patients with available consumer attribute data (59.1%): 6.4% Hispanic, 2.6% Non-Hispanic Asian, 5.3% Non-Hispanic Black, 73.7% Non-Hispanic White, 12.0% Other
Levy *et al*. (2025)	Israel	Feb 2024	Cohort study	Lung transplant recipients	Lung transplant recipients	NA	Adverse events	28	Median (IQR) age: 62 (53–67) years; 25.0% females
Li *et al*. (2025)	USA	May 3, 2023–Dec 27, 2024	Cross-sectional study	Adults ≥60 years old	Adults ≥60 years old	NA	Adverse events	4544	Median age: 73 years; 69.6% females
Lloyd *et al*. (2025)–preprint	USA	May 3, 2023–Jan 28, 2024	Case series	Adults ≥60 years old	Adults ≥60 years old	NA	Adverse events	3,226,689	18.8% were aged 65–69 years, 29.9% were aged 70–74 years, 25.0% were aged 75–79 years, 15.2% were aged 80–84 years, 7.2% were aged 85–89 years, 3.8% were aged ≥90 years; 57.5% females; Race: 89.4% White, 2.7% Black or African American, 0.4% Hispanic, 1.8% Asian, 0.2% AIAN, 1.9% other, 3.7% unknown
Lotscher *et al*. (2025)	Switzerland	Dec 2023–Dec 2024	Cohort study	Allogeneic hematopoietic stem cell transplant recipients	Allogeneic hematopoietic stem cell transplant recipients	Secondary care	Adverse events	82	34.1% females
Mensah *et al*. (2025)	England, UK	Nov 4, 2024–Jan 6, 2025	Quasi-experimental (regression discontinuity design)	Adults 75–79 years old	Adults 75–79 years old	Secondary care	Vaccine uptake (population-based); VE against RSV-associated hospitalisations	2,541,696	No population characteristics reported
Morrison *et al*. (2025)	Canada	Feb 2024	Cross-sectional study	Adults ≥60 years old	Adults ≥60 years old	Long-term care homes	Vaccine uptake	NA	No population characteristics reported
Motta *et al*. (2025)	USA	Oct 20–Nov 6, 2023	Cross-sectional study	Adults ≥60 years old	Adults ≥60 years old	Community	Vaccine uptake (population-based)	358	Mean (SD) age: 69.4 (6.7) years; 55.0% females; Race: 73% White, 11% Black or African, 10% Hispanic or Latino, 1% Asian or Asian American, 1% Native American, 2% multiple races, 2% other
Murphy *et al*. (2025)	USA	Jul 1, 2023–Jun 30, 2024	Cohort study	Adults ≥65 years old	Adults ≥65 years old	Community	Vaccine uptake (population-based)	15,841,938	Mean (SD) age: 76.1 (7.3) years; 58.0% females; Race: 87.1% White, 4.6% Black or African American, 1.4% Hispanic, 2.2% Asian, 4.7% other
Nguyen *et al*. (2025)	Australia	Feb 29, 2024–Sep 27, 2025	Cross-sectional study	Adults ≥60 years old	Adults ≥60 years old	Community	Adverse events	2013	Median (IQR) age: 75 (70–80) years; 62.0% females
Patrick *et al*. (2025)	USA	Sep 23, 2023–Apr 9, 2024	Cohort study	Adults ≥60 years old	Adults ≥60 years old	Community	Vaccine uptake (population-based)	1,003,132	49.4% were aged 60–69 years, 34.6% were aged 70–79 years, 16.0% were aged ≥80 years; 54.3% females, Ethnicity and race: 42.5% White (non-Hispanic), 31.4% Hispanic, 12.0% Asian (non-Hispanic), 9.4% Black or African American (non-Hispanic), 2.1% multiple or other, 0.2% AIAN, 2.4% unknown
Payne *et al*. (2024)	USA	Oct 1, 2023–Mar 31, 2024	Test-negative case–control study	Adults ≥60 years old	Adults ≥60 years old hospitalised with RSV-associated ARI	Secondary care	Vaccine uptake; VE against RSV-associated hospitalisations	36,706 episodes	Median (IQR) age: 76 (69–84) years; 52.5% females; Ethnicity and race: 74% White (non-Hispanic), 9% Hispanic, 8% Black or African American (non-Hispanic), 9% other (non-Hispanic), 1% unknown
(as above)	USA	Oct 1, 2023–Mar 31, 2024	Test-negative case–control study	Adults ≥60 years old	Adults ≥60 years old at ED with RSV-associated ARI	Secondary care	Vaccine uptake; VE against RSV-associated ED visits	37,842 episodes	Median (IQR) age: 75 (67–82) years 54.9% females; Ethnicity and race: 66% White (non-Hispanic), 12% Hispanic, 9% Black or African American (non-Hispanic), 13% other (non-Hispanic), 1% unknown
Redjoul *et al*. (2025)	France	Oct 1–Nov 21, 2024	Cohort study	Allogeneic hematopoietic stem cell transplant recipients	Allogeneic hematopoietic stem cell transplant recipients	Secondary care	Adverse events	92	Median (IQR) age: 63 (53–70) years; 33.7% females
Reses *et al*. (2023)	USA	2023/24 season (till Dec 10, 2023)	Cohort study (surveillance study)	Adults ≥60 years old	Nursing home residents	Nursing homes	Vaccine uptake (population-based)	238,449	NA (no population demographics provided)
Reses *et al*. (2024)	USA	2024/25 season (till Nov 10, 2024)	Cohort study (surveillance study)	Adults ≥60 years old	Nursing home residents	Nursing homes	Vaccine uptake (population-based)	661,075	NA (no population demographics provided)
Rizzo *et al*. (2025)	USA	Aug 1, 2023–Jun 30, 2024	Cross-sectional study	Adults ≥60 years old	Adults ≥60 years old who received seasonal influenza vaccine	Community	Vaccine uptake	4,471,042	Median (IQR) age: 71 (65–78) years; 54.9% females; Race: 44.9% White, 19.0% Latino, 14.7% Asian, 4.1% Black or African American, 1.0% Multiracial, 0.4% NHOPI, 12.6% other, 0.4% AIAN, 2.9% unknown
Surie *et al*. (2024)	USA	Oct 1, 2023–Mar 31, 2024	Test negative case–control study	Adults ≥60 years old	Adults ≥60 years old, hospitalised due to ARI	Secondary care	Vaccine uptake; VE against RSV-associated hospitalisations	2978	Median (IQR) age: 72 (66–80) years; 51.2% females; Ethnicity and race: 62.7% White (non-Hispanic), 11.3% Hispanic or Latino, 19.5% Black or African American (non-Hispanic), 6.5% other
Surie *et al*. (2025a)	USA	Oct 1, 2023–Apr 30, 2024	Cross-sectional study	Adults ≥60 years old	Adults ≥60 years old, hospitalised with RSV-negative ARI	Secondary care	Vaccine uptake	6746	Median (IQR) age: 73 (66–80) years; 51.2% females; Ethnicity and race: 65.1% White (non-Hispanic), 10.6% Hispanic, 19.5% Black or African American (non-Hispanic), 4.7% other
Surie *et al*. (2025b)	USA	Oct 1, 2023–Mar 31, 2024; Oct 1, 2024–Apr 30, 2025	Test-negative case–control study	Adults ≥60 years old	Adults ≥60 years old, hospitalised with ARI	Secondary care	Vaccine uptake; VE against RSV-associated hospitalisations and severe in-hospital outcomes	6958	Median (IQR) age: 72 (66–80) years; 50.8% females; Ethnicity and race: 11.0% Hispanic or Latino, 20.7% Non-Hispanic Black or African American, 62.0% Non-Hispanic White, 4.4% Other, 1.9% unknown
Symes *et al*. (2025)	England, UK	Oct 1, 2024–Mar 31, 2025	Test-negative case–control study	Adults 75–79 years old	Adults 75–79 years old with ARI-related hospital admission	Secondary care	Vaccine uptake; VE against RSV-associated hospitalisation and severe disease (ICU, supplemental oxygen)	1006	Median (IQR) age: 77 (76–78) years; 52.3% females; Ethnicity: 89.0% White, 0.3% Mixed or multiple ethnic groups, 4.2% Asian or Asian British, 1.1% Black or Black British or Caribbean or African, 1.4% other, 4.1% missing data
Tartof *et al*. (2024)	USA	Nov 24, 2023–Apr 9, 2024	Test-negative case–control study	Adults ≥60 years old	Adults ≥60 years old hospitalised or at ED with LRTI	Secondary care	Vaccine uptake; VE against RSV-related hospitalisations or ED visits, severe disease (supplemental oxygen)	7047	Mean (SD) age: 76.8 (9.6) years; 54.2% females; Ethnicity and race: 36.9% White (non-Hispanic), 33.0% Hispanic, 17.0% Black or African American (non-Hispanic), 11.9% NHOPI (non-Hispanic), 1.2% multiple or other or unknown
Tartof *et al*. (2025)	USA	Nov 24, 2023–Apr 9, 2024	Test-negative case–control study	Adults ≥60 years old	Adults ≥60 years old hospitalised or at ED with ARI	Secondary care	Vaccine uptake; VE against RSV-related ARI hospitalisations or ED visits, severe disease (supplemental oxygen)	8965	Mean (SD) age: 77.8 (1.4) years; 54.7% females; Ethnicity and race: 36.7% White (non-Hispanic), 33.7% Hispanic, 16.7% Black or African American (non-Hispanic), 11.8% NHOPI (non-Hispanic), 1.1% multiple or other or unknown
Viskupic *et al*. (2025)	USA	Jul 2023–Apr 2024	Cross-sectional study	Adults ≥60 years old	Adults ≥60 years old	Community	Vaccine uptake (population-based)	376	Mean age: 71 years; 47.9% females

**Note**: The characteristics of Fry *et al*. (2025), Hause *et al*. (2024), and Payne *et al*. (2024) studies are each described in two separate rows because each of these studies presented data for two separate study populations with different outcomes.

**Abbreviations**: AIAN = American Indian or Alaska Native; ARI = acute respiratory infection; ED = emergency department; ICU = intensive care unit; IQR = interquartile range; LRTI = lower respiratory tract infection; NA = not applicable; NHOPI = Native Hawaiian or Other Pacific Islander; RSV = respiratory syncytial virus; SD = standard deviation; VE = vaccine effectiveness.

### RSV vaccine uptake and influencing factors for low/high uptake

**Fig. 2:**
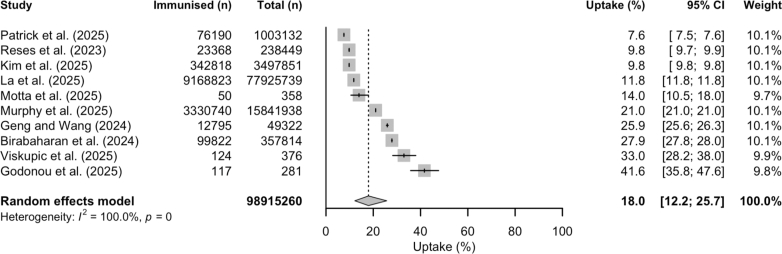
**Uptake (%) of RSV vaccines among eligible adults (60 years or older) in the United States during the 2023/24 RSV season**. **Abbreviations**: 95% CI = 95% confidence interval (shown with error bars); n = number of individuals.

During the 2023/24 RSV season in the United States, the overall meta-estimate for uptake of RSV vaccines among adults aged 60 and older was 18.0% (95% CI: 12.2–25.7) from ten population-based studies (Fig. 2).^9^^,^^36^^,^^38^, ^39^, ^40^, ^41^, ^42^, ^43^^,^^45^^,^^53^ Sensitivity analysis pooling data from studies assessed at ‘low’ risk of bias did not significantly change the estimate (Supplementary materials, p 17). Subgroup analyses by study design showed uptake of 16.8% (95% CI: 9.3–28.6; six studies) for cohort studies and 19.8% (95% CI: 11.9–31.2; four studies) for cross-sectional studies. One study reported uptake of 58.5% in long-term care homes in Ontario, Canada during the 2023/24 RSV season.^57^

As shown in Table 2, higher uptake was seen in older adults aged 75 years and older compared to the 60–74 age group (OR 1.58 (95% CI: 1.40–1.79); 12 studies). Uptake was higher in older adults with any reported comorbidity compared to those without comorbidities (OR 1.52 (95% CI: 1.17–1.98); five studies) and older adults with immunocompromised status compared to immunocompetent individuals (OR 1.47 (95% CI: 1.21–1.80); five studies). Data pooled from five studies showed that older adults with cardiovascular disease (OR 1.73; 95% CI: 1.26–2.37), metabolic or endocrinologic disease (OR 1.57; 95% CI: 1.18–2.08), kidney disease (OR 1.57; 95% CI: 1.10–2.25), and lung disease (OR 1.96; 95% CI: 1.37–2.82) had a higher RSV vaccine uptake than older adults without comorbidities. Having two comorbidities compared to no comorbidities was also associated with a higher vaccine uptake (OR 1.76 (95% CI: 1.41–2.19); five studies). Lower vaccine uptake was seen in Black or African American (non-Hispanic) population (OR 0.51 (95% CI: 0.43–0.60); 12 studies), Hispanic populations (OR 0.49 (95% CI: 0.40–0.60); 12 studies), and non-Hispanic racial groups described as Mixed or Other (OR 0.80 (95% CI: 0.70–0.92); 11 studies) compared to White (non-Hispanic) population. Uptake was also lower in Hispanic older adults than in non-Hispanic groups (OR 0.53 (95% CI: 0.43–0.64); 12 studies). Forest plots for all subgroup meta-analyses are available in Supplementary Materials (pp 18–40). Sensitivity analyses with studies at ‘low’ risk of bias did not significantly change the subgroup meta-estimates (Supplementary Materials, pp 18–40).

**Table 2:** Meta-analysis of RSV vaccine uptake in adults aged 60 years and older reported in at least three studies from the United States.

Variable	Subgroup	Reference group	No. of studies	Odds ratio (95% CI)	I^2^
Age group	≥75 years	60–74 years	12	1.58 (1.40–1.79)	100.0%
Sex	Males	Females	10	1.00 (0.96–1.03)	96.1%
Comorbidities	Any comorbidity	No comorbidities	5	1.52 (1.17–1.98)	99.9%
	Immunocompromised	Not immunocompromised	5	1.47 (1.21–1.80)	97.3%
	Cardiovascular disease	No comorbidities	5	1.73 (1.26–2.37)	100.0%
	Metabolic or endocrinologic disease	No comorbidities	5	1.57 (1.18–2.08)	100.0%
	Kidney disease	No comorbidities	5	1.57 (1.10–2.25)	100.0%
	Lung disease	No comorbidities	5	1.96 (1.37–2.82)	100.0%
Number of comorbidities	One comorbidity	No comorbidities	5	1.52 (1.26–1.84)	99.9%
	Two comorbidities	No comorbidities	5	1.76 (1.41–2.19)	99.9%
	Two comorbidities	One comorbidity	5	1.14 (1.06–1.22)	99.3%
Race and ethnicity	Black or African American (non-Hispanic)	White (non-Hispanic)	12	0.51 (0.43–0.60)	99.8%
	Asian (non-Hispanic)	White (non-Hispanic)	9	0.89 (0.70–1.12)	99.6%
	Hispanic	White (non-Hispanic)	12	0.49 (0.40–0.60)	99.7%
	Mixed or Other (non-Hispanic)	White (non-Hispanic)	11	0.80 (0.70–0.92)	97.8%
	Hispanic	Non-Hispanic	12	0.53 (0.43–0.64)	100.0%

**Abbreviations**: RSV = respiratory syncytial virus; 95% CI = 95% confidence interval.

In the United Kingdom, RSV vaccine uptake halfway through the 2024/25 RSV season among older adults aged 75–79 years old were reported in two population-level studies. Uptake reached 68.6% in Scotland (by December 8, 2024) and 46.6% in England (by January 6, 2025).^54^^,^^55^ One study reported uptake of 17.9% in nursing care homes in the United States at the start of the 2024/25 RSV season (by November 10, 2024).^46^

### RSV vaccine effectiveness

**Fig. 3:**
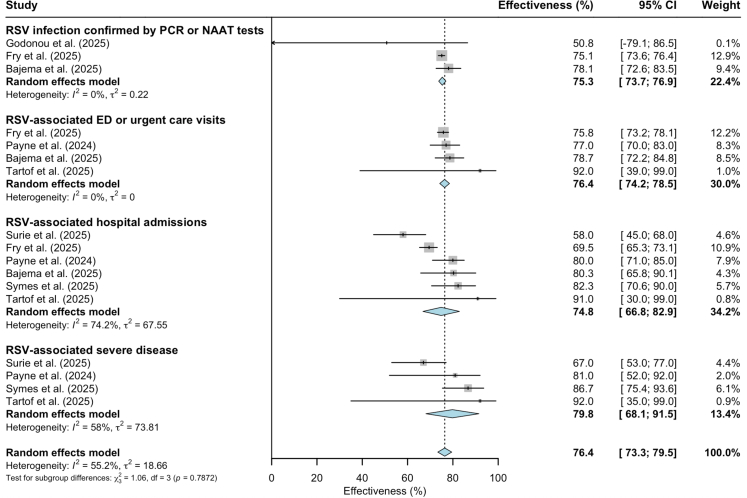
**RSV vaccine effectiveness (%) against RSV-associated disease outcomes in older adults (60 years or older)**. **Note**: RSV-associated severe disease is defined as admissions to intensive care unit (ICU), use of supplemental oxygen, or in-hospital death. **Abbreviations**: 95% CI = 95% confidence interval (shown with error bars); PCR = polymerase chain reaction; NAAT = nucleic acid amplification test; RSV = respiratory syncytial virus; ED = emergency department.

Seven studies from the United States and United Kingdom reporting RSV vaccine effectiveness in older adults were included in the meta-analysis (Fig. 3).^37^^,^^39^^,^^44^^,^^50^^,^^52^^,^^56^^,^^58^ Sensitivity analysis with only studies at ‘low’ risk of bias did not significantly alter the results of any meta-analyses (Supplementary Materials, pp 41).

Effectiveness against any RSV infection confirmed by polymerase chain reaction (PCR) or nucleic acid amplification test (NAAT) in adults aged 60 years and older pooled from three studies was 75.3% (95% CI: 73.7–76.9, p < 0.001). Two studies reported effectiveness in preventing any laboratory-confirmed RSV infections stratified by age group with similar effectiveness estimates to the pooled estimate.^37^^,^^58^ One study found a lower effectiveness of 70.4% (95% CI: 67.8–72.7) among older adults aged 60 years and older with an immunocompromised status^37^; however, another study did not report a significant difference in this group.^58^ Additionally, one study focusing on solid organ transplant recipients and stem cell transplant recipients aged 60 and older reported vaccine effectiveness of 73.4% (95% CI: 61.9–81.4) and 33.4% (95% CI: 12.3–49.4), respectively, in these patients.^37^

The pooled effectiveness from four studies in preventing RSV-associated emergency department (ED) or urgent care visits in older adults aged 60 and above was 76.4% (95% CI: 74.2–78.5, p < 0.001). No significant differences in effectiveness were observed by age group (two studies)^37^^,^^44^ or in immuno-compromised people (one study).^37^ One study reported a lower effectiveness in transplant recipients aged 60 and older (58.4% (95% CI: 37.4–72.3)).^37^ In one study, *Arexvy* (GSK) and *Abrysvo* (Pfizer) vaccines were assessed separately with effectiveness against ED visits of 77% (95% CI: 70–83) and 79% (95% CI: 59–89), respectively, in adults aged 60 or older.^44^

Effectiveness against RSV-associated hospital admissions pooled from six studies among adults aged 60 years or older was 74.8% (95% CI: 66.8–82.9, p < 0.001). Subgroup meta-analyses stratified by age group showed similar effectiveness against RSV-associated hospitalizations in adults aged 60–74 years (65.6% (95% CI: 47.6–83.6; three studies) and 75 years or older (75.4% (95% CI: 70.0–80.8); four studies). In immunocompromised older adults, pooled effectiveness against RSV-related hospitalizations was slightly lower but the 95% confidence intervals overlapped (60.3% (95% CI: 45.0–75.6); four studies). Fry *et al*. (2025) reported lower effectiveness in transplant recipients: 55.9% (95% CI: 40.0–67.5).^37^ Vaccine effectiveness in preventing RSV-related hospitalizations in older adults (60 years and older) was reported by Payne *et al*. (2024) separately for *Arexvy* (GSK) and *Abrysvo* (Pfizer) vaccines as 83% (95% CI: 73–89) and 73% (95% CI: 52–85), respectively.^44^ A study from England in older adults aged 75–79 years explored vaccine effectiveness against RSV-related hospitalizations by admission reason. This study reported effectiveness of 88.6% (95% CI: 75.6–95.6), 77.4% (95% CI: 42.4–92.8), and 78.8% (95% CI: 47.8–93.0) for people admitted with LRTI, lung disease exacerbation without LRTI, and lung disease, heart disease or frailty exacerbation without LRTI, respectively.^56^ Vaccine effectiveness against RSV-associated severe disease, which was defined as ICU admission, use of supplemental oxygen or in-hospital death, pooled from four studies was 79.8% (95% CI: 68.1–91.5, p < 0.001).

### RSV vaccine impact at population level

A regression discontinuity design study from Scotland compared RSV-related hospitalization rates in older adults before and after the introduction of RSFpreF vaccine (*Abrysvo*, Pfizer) on August 12, 2024. Rates in the population eligible for vaccination (74–79 years of age) were compared with non-eligible age groups (70–73 and 80–84 years of age). The study found a 62.1% (95% CI: 35.0–79.8) reduction in RSV-associated hospitalizations among the eligible age group by December 8, 2024, with a population vaccine coverage of 68.6%.^54^ A similar design study investigating the vaccine impact in England, where RSFpreF vaccine (*Abrysvo*, Pfizer) was introduced on September 1, 2024, showed a 30% (95% CI: 18–40) reduction in RSV-related hospital admissions in older adults eligible for vaccination by January 6, 2025, where uptake was 46.6%.^55^

### RSV vaccine safety

**Fig. 4:**
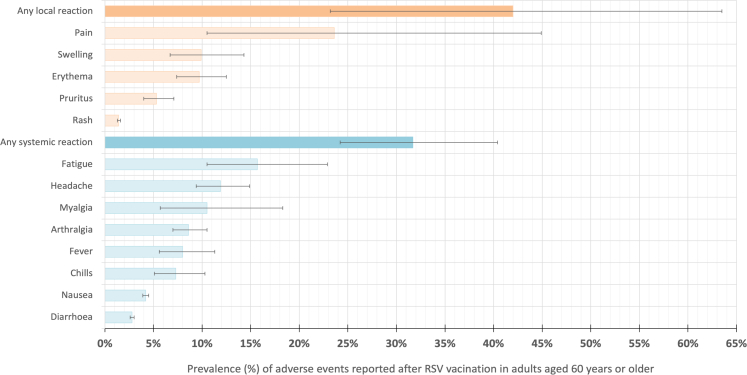
**Meta-analysis of adverse event prevalence (%) in older adults aged 60 years and older after RSV vaccination reported in at least three studies**. **Abbreviation**: 95% CI = 95% confidence intervals (shown with error bars). Specific numbers are available in Supplementary 8 (page 43).

Seven studies reporting adverse events were included in the meta-analysis (Fig. 4).^59^^,^^62^^,^^65^, ^66^, ^67^, ^68^^,^^70^ estimates for all local injection site reactions after vaccination were reported in three studies with a pooled prevalence of 42.0% (95% CI: 23.2–63.5). Data on combined systemic adverse reactions were reported in three studies with a prevalence meta-estimate of 31.7% (95% CI: 24.2–40.4).

The risk of Guillain-Barré syndrome (GBS) following RSV vaccination was reported in four studies.^37^^,^^59^^,^^61^^,^^63^ An initial signal was seen in the United States through the V-Safe and VAERS voluntary surveillance systems with Hause *et al*. (2024) estimating 1.8 and 4.4 GBS reports per one million doses for *Arexvy* (GSK) and *Abrysvo* (Pfizer), respectively.^59^ Donahue *et al*. (2025) compared risk interval of 1–21 days post vaccination *versus* 43–63 day control interval post vaccination and did not observe a statistically significant risk of GBS during the risk interval (for *Arexvy* (GSK), adjusted rate ratio: 3.58 (95% CI: 0.29–113.17)).^63^ Two self-controlled case series studies compared the incidence of GBS within the period of 1–42 days after vaccination to the control period 43–90 days post vaccination. Both studies from the United States utilised large electronic health record databases identifying cases of GBS by ICD-10 coding with Lloyd *et al*. (2025) also carrying out a medical record review. They found that the attributable risk of GBS after *Arexvy* (GSK) vaccination was 5.2 (incidence rate ratio (IRR) 1.5; 95% CI: 0.9–2.2) and 6.5 (IRR 2.46; 95% CI: 1.19–5.08) cases per one million vaccine doses; after *Abrysvo* (Pfizer) vaccination, the reported attributable risk of GBS was 18.2 (IRR 2.4; 95% CI: 1.5–4.0) and 9.0 (IRR 2.02; 95% CI: 0.93–4.40) cases per one million doses.^37^^,^^61^

Five studies with under 100 participants each explored adverse events in transplant recipients after receiving an RSV vaccine.^64^^,^^67^, ^68^, ^69^, ^70^ In lung transplant and hematopoietic stem cell transplant recipients, local adverse events pooled from three studies were reported in 36.5% (95% CI: 21.8–54.3) of vaccinees. A study from Czech Republic in lung transplant recipients over the age of 60 years found systemic adverse reactions in 16.7% (95% CI: 3.3–30.0) of vaccinees.^67^

A study from Italy observed fewer adverse events reported after vaccination with each one-year increase in vaccine recipients’ age: OR 0.92 (95% CI: 0.89–0.95).^65^ One study reported no increased risk of new-onset atrial fibrillation (risk ratio (RR) 1.06 (95% CI: 0.90–1.25)) and a decreased risk of recurrent atrial fibrillation (RR 0.94 (95% CI: 0.91–0.97) within 42 days of receiving RSV vaccine compared to influenza vaccine.^36^ Other adverse events that were reported in less than three studies are summarised in the Supplementary Materials (pp 42–84).

### Publication bias assessment

Visual inspection of a funnel plot showed symmetry (Supplementary Materials, p 85), and the Egger's test indicated no significant publication bias in the meta-analyses for uptake (p = 0.750). However, publication bias could not be assessed for effectiveness and safety because fewer than 10 studies were included.

## Discussion

During the 2023/24 and 2024/25 RSV seasons, uptake of RSV vaccines among older adults was low and substantial disparities were observed by population subgroups in the United States. Our findings reflected high RSV vaccine effectiveness in preventing healthcare utilisation using different outcome metrics. Although studies showed an overall favourable safety profile, a slight increase in Guillain-Barré syndrome (GBS) incidence post vaccination was seen in two independent hypothesis testing studies from the United States.

Our overall population-based vaccine uptake estimates in the United Kingdom and United States were in line with reports from public health agencies and governmental organizations. On June 29, 2024, U.S. Centers for Disease Control and Prevention (CDC) reported that 21.0% of older adults aged 65 years and older had received an RSV vaccination during the 2023/24 RSV season.^71^ When RSV vaccines were first introduced in the United States in June 2023, the CDC recommended the use of shared clinical decision-making (SCDM) between patients and healthcare providers when deciding on whether to receive the vaccine.^1^^,^^72^ Studies have reported that SCDM recommendation may have led to lower vaccine uptake during the 2023/24 RSV season due to the need to arrange individual medical consultations, access and transportation barriers arising from socio-economic factors, and misunderstandings from health insurance companies.^38^^,^^42^^,^^49^ In June 2024, SCDM recommendation was removed, and RSV vaccines were indicated for all adults aged 75 years and older, as well as those aged 60–74 years at high risk for severe RSV-related disease.^1^ As of April 26, 2025, CDC estimates have shown higher vaccine uptake following this change in recommendations: 38.1% (95% CI: 35.7–40.4) and 47.5% (95% CI: 45.9–49.1) in 60–74 age group and 75 and older age group, respectively. In England, UK Health Security Agency (UKHSA) reported RSV vaccine coverage of 62.9% by June 30, 2025, among older adults aged 75–79 years old.^73^ Continued analyses on how changes in vaccination guidelines affect uptake are needed to inform RSV immunisation strategies in the United States and in other countries.

In our meta-analysis, older adults in the United States with risk factors for severe RSV-associated disease, including advanced age, presence of comorbidities and immunocompromised status, had higher RSV vaccine uptake estimates than older adults without these risk factors. Patrick *et al*. (2025) reported variations in vaccine coverage by comorbidities with higher uptake found among older adults who had lung conditions or immunodeficiencies than those with kidney disease or oncologic conditions.^43^ Their findings suggested differences in access and provider recommendations by underlying health conditions which warrant further investigation. We found RSV vaccine uptake was significantly lower among non-White racial groups compared to White groups in the United States. Similarly, CDC reported substantial differences in RSV vaccine uptake by race/ethnicity ranging from 7.1% in Hispanic populations to 21.9% in White (non-Hispanic) groups.^71^ Notably, UKHSA reported differences in uptake by ethnic groups in England as well, ranging from 21.9% in Pakistani populations to 65.2% among White British ethnic groups.^73^ Similar disparities in vaccine coverage have been reported with other vaccines such as SARS-CoV-2, human papillomavirus (HPV), and influenza vaccines.^74^ During the 2023/24 season, seasonal influenza vaccine uptake in the United States was lower among Black and Hispanic older adults than in White individuals of similar age.^75^ These findings may reflect ethnic differences in individual-level factors such as attitudes towards vaccines, economic considerations, as well as cultural and environmental factors including barriers to accessing vaccination services.

Since our study focuses on population-level cohorts with a pre-specified protocol, we did not include clinical trial studies. Reassuringly, vaccine effectiveness findings in our study were similar to estimates reported in clinical trials. Meta-analyses showed vaccine effectiveness between 74.8% and 79.8% in preventing different RSV-related healthcare utilisation outcomes. Although the 95% CIs for all these meta-estimates overlapped, effectiveness was higher against severe RSV-related disease (ICU admission, supplemental oxygen, death). Higher vaccine effectiveness against the most severe disease is the primary aim of RSV vaccines and similar pattern has also been reported with SARS-CoV-2 vaccines.^76^ No differences in effectiveness were seen between adults aged 60–74 years compared to adults aged 75 years and older.^77^

Several knowledge gaps regarding RSV vaccine effectiveness remain. First, a slightly lower vaccine effectiveness was observed in immunocompromised older adults. When focusing on stem cell transplant recipients, effectiveness against RSV-related healthcare utilisation outcomes was significantly lower.^37^ This may be due to immuno-suppressive medications used in transplant recipients which interfere with immune responses and therefore the vaccines’ effectiveness.^78^ More data are needed to evaluate how RSV vaccine effectiveness in immunocompromised older adults may differ from healthy older individuals. Second, RSV infections might put additional strain on patients with chronic lung or cardiovascular conditions, increasing the risk of exacerbations of the underlying comorbidities. Symes *et al*. (2025) reported high vaccine effectiveness in preventing RSV-positive hospital admissions in older adults who were admitted primarily due to chronic lung disease exacerbations (without LRTI).^56^ These findings outline potential further benefits of RSV vaccines. Third, potential differences between the RSV vaccine products with regards to effectiveness need to be further investigated. Fourth, continued surveillance of vaccine effectiveness in vaccinated individuals over the coming RSV seasons is needed to establish the durability of effectiveness from one vaccine dose and determine optimal timeframe for revaccination.

GBS is an autoimmune disorder which affects the peripheral nervous system leading to weakness in limbs or muscles, reduced deep tendon reflexes, tingling sensation, and pain of varying severity. A systematic review and meta-analysis estimated the background incidence of GBS to be approximately 22.2 cases per million people aged 70–79 years old per year and 26.6 cases per million people per year in the 80 to 89 age group.^79^ Studies included in this review estimated the attributable risk of GBS to be between 5.2 and 6.5 per one million *Arexvy* (GSK) vaccine doses and between 9.0 and 18.2 per one million *Abrysvo* (Pfizer) vaccine doses. The 95% confidence intervals for the two vaccine products overlapped; therefore, the differential risk between vaccine products needs to be investigated in further studies. Although other vaccines have also been associated with a slight increase in GBS risk, the reported risk associated with RSV vaccines is higher. For instance, seasonal influenza vaccines and herpes zoster vaccines were reported to be associated with less than one excess GBS case per one million and three GBS cases per one million vaccine doses, respectively.^80^^,^^81^ A more granular analysis of the severity and duration of post-vaccination GBS is required to further demonstrate the associated risks. One study reported no increased risk of new-onset atrial fibrillation (risk ratio (RR) 1.06 (95% CI: 0.90–1.25)) and a decreased risk of recurrent atrial fibrillation (RR 0.94 (95% CI: 0.91–0.97) within 42 days of receiving RSV vaccine compared to influenza vaccine.^36^ Domnich *et al*. (2025) suggested that older vaccine recipients were less likely to report adverse events after vaccination, which could be due to higher adverse event tolerance in older adults or reduced innate immune responses compared to younger individuals.^65^ When analysing safety data, it is important to consider that variations in vaccine eligibility among countries, for instance by age group, may lead to different rates of adverse events reported in studies. Longer monitoring after vaccination and studies with larger sample sizes may help identify late-onset adverse events, describe prevalence of rare adverse events, and characterise safety profiles in more granular population subgroups.

To our knowledge, this is the first systematic review and meta-analysis to summarize RSV vaccine uptake, effectiveness and safety data in older adult populations from real-world studies conducted across countries in North America, Europe, Asia, and Australia, covering more than 121.8 million individuals. We were able to conduct meta-analyses for several vaccine effectiveness outcomes, prevalence of specific adverse events following vaccination, and compare vaccine uptake and performance in different sociodemographic and clinical subpopulations. The pooled estimates of RSV vaccine effectiveness against any laboratory-confirmed RSV infection and ED or urgent care visits showed low study heterogeneity while the pooled estimates against hospital admissions and severe RSV-associated disease showed some heterogeneity (Fig. 3). When only including studies at ‘low risk of bias’ according to the quality assessment, the pooled estimate against RSV-associated hospitalisations also had low I^2^ value. (Supplementary Materials, p 41). These findings indicate good consistency among the included studies and reliability of our results.

However, our study is subject to several limitations. First, the field is rapidly changing with new data emerging monthly, which requires continued consistent monitoring to ensure that evidence is up to date. As more studies are published care needs to be taken not to report data from the same populations multiple times which may lead to biased estimates. Second, we did not include data reported by public health agencies in this review—literature review is unlikely to capture national statistics on public health websites or dashboards; however, our findings are similar to those reported by CDC and UKHSA. Third, the studies included varied substantially by study designs, outcome definitions, study periods and populations leading to high heterogeneity (I^2^ statistic) among studies reporting vaccine uptake and safety data. For the pooled estimates, 95% CIs were wide due to studies with small sample sizes. Fourth, when summarising the safety signals, we used prevalence of adverse events reported among vaccinated individuals. As there were no comparison groups of unvaccinated participants, these estimates do not fully account for background rates of these events and should be interpreted with caution. Fifth, there is currently limited data on RSV vaccines in older adults from outside the United States which limits generalisability to other countries. However, three studies from Europe have been added from the updated search in November 2025 and we have updated the results accordingly. Nevertheless, real-world evidence is needed from more countries. Lastly, although we tried to avoid multiple publications from the same research group by including only the most recent report, it was inevitable to avoid potential overlap of participants across different studies.

Our findings provide a comprehensive overview of RSV vaccine uptake and performance in older adults, as well as identify several knowledge gaps to be addressed in future research. These findings might help policy makers and healthcare providers tailor RSV immunisation programmes to optimise the benefits of reduced RSV disease burden on individual level and on population level, and to increase public awareness and confidence in RSV vaccines.

## Contributors

TS conceptualised the study with input from DT. DT, BL, SF, LL and CB contributed to data collection. DT led data analysis with input from BL and SF. DT wrote the codes for analysis. TS, AA, JS, AM, WSL, KM and CG led the data interpretation. DT wrote the first draft of the manuscript with input from TS and SF. All authors contributed to data interpretation and critically revised the manuscript. All authors read and approved the final version of the manuscript. DT, BL and TS had full access to and verified the underlying data of the study. All authors had final responsibility for the decision to submit for publication.

## Data sharing statement

Deidentified summary data and analysis codes are available at https://github.com/dtrusinska/RSV-vaccines-olderadults-meta-analysis-code.

## Declaration of interests

DT is funded by Health Data Research UK Inflammation and Immunity Driver Programme. BL is funded by Asthma + Lung UK Early Career Grant. WSL's institution has received unrestricted investigator-initiated research funding from Pfizer for an unrelated multi-centre study in pneumonia in which WSL was the Chief Investigator (study ended 31 Dec 2023). WSL's institution has received research funding from National Institute of Health and Care Research (NIHR) for unrelated studies in which WSL is the Chief Investigator or co-applicant. WSL is chair of Joint Committee on Vaccination and Immunisation (JCVI)-this is an unpaid role. WSL is chair of the Acute Respiratory Infection (ARI) National Research Strategy Group (NRSG)–this is an unpaid role. All other authors declare no competing interests.
