# Supplementary material for: Real-world evidence on RSV vaccine uptake, effectiveness, and safety in older adults: a systematic review and meta-analysis
Source: Lancet Reg Health Eur. 2026 Feb 20;64:101623. doi: 10.1016/j.lanepe.2026.101623 (PMC12936786; doi:10.1016/j.lanepe.2026.101623)
Supplement: Supplementary Materials [file mmc1.docx]

**Supplementary materials for “Real-world evidence on RSV vaccine uptake, effectiveness, and safety in older adults: a systematic review and meta-analysis”**

**Table of Contents**

[Supplement 1: Search strategies 4](#_Toc220619007)

[Supplement 2: Study selection criteria 6](#_Toc220619008)

[Supplement 3: List of excluded studies 7](#_Toc220619009)

[Supplement 4: ‘Risk-of-bias’ quality assessment 11](#_Toc220619010)

[Supplement 5: Sensitivity analysis for RSV vaccine uptake 17](#_Toc220619011)

[Supplement 6: RSV vaccine uptake in the United States by subgroup (as reported in Table 2) 18](#_Toc220619012)

[Supplement 7: Sensitivity analysis for RSV vaccine effectiveness in older adults 42](#_Toc220619013)

[Supplement 8: Meta-analysis of adverse event prevalence (%) in older adults aged 60 years and older after RSV vaccination 43](#_Toc220619014)

[Supplement 9: Overview of all reported safety signals 44](#_Toc220619015)

[Any adverse reaction 44](#_Toc220619016)

[Local adverse reactions 44](#_Toc220619017)

[Any local reaction 44](#_Toc220619018)

[Bruising 45](#_Toc220619019)

[Cellulitis 45](#_Toc220619020)

[Discharge 45](#_Toc220619021)

[Discolouration 46](#_Toc220619022)

[Discomfort 46](#_Toc220619023)

[Erythema 46](#_Toc220619024)

[Hypoaesthesia 47](#_Toc220619025)

[Induration 48](#_Toc220619026)

[Inflammation 48](#_Toc220619027)

[Irritation 48](#_Toc220619028)

[Itching 49](#_Toc220619029)

[Mass 49](#_Toc220619030)

[Mobility (reduced) 49](#_Toc220619031)

[Nodule 50](#_Toc220619032)

[Pain 50](#_Toc220619033)

[Pruritus 52](#_Toc220619034)

[Rash 52](#_Toc220619035)

[Scab 53](#_Toc220619036)

[Swelling 53](#_Toc220619037)

[Ulcer 54](#_Toc220619038)

[Underarm swelling/tenderness 55](#_Toc220619039)

[Warmth 55](#_Toc220619040)

[Systemic adverse reactions 55](#_Toc220619041)

[Any systemic reaction 55](#_Toc220619042)

[Abnormal limb (detected on x-ray) 56](#_Toc220619043)

[Ascending flaccid paralysis 56](#_Toc220619044)

[Allergic reaction 56](#_Toc220619045)

[Anaphylaxis 57](#_Toc220619046)

[Arrhythmia 57](#_Toc220619047)

[Arthralgia 57](#_Toc220619048)

[Asthenia 59](#_Toc220619049)

[Atrial fibrillation 59](#_Toc220619050)

[Bell’s palsy 60](#_Toc220619051)

[Cellulitis 60](#_Toc220619052)

[Cerebrospinal fluid (CSF) protein (increased) 61](#_Toc220619053)

[Chills 61](#_Toc220619054)

[Congestive heart failure 62](#_Toc220619055)

[Death 62](#_Toc220619056)

[Deep venous thrombosis 62](#_Toc220619057)

[Diarrhea 63](#_Toc220619058)

[Discomfort 64](#_Toc220619059)

[Dyspnea or cough 64](#_Toc220619060)

[Ecchymosis 64](#_Toc220619061)

[Encephalitis or aseptic meningitis 64](#_Toc220619062)

[Erythema (systemic) 65](#_Toc220619063)

[Fatigue/malaise/tiredness 66](#_Toc220619064)

[Fever/pyrexia 67](#_Toc220619065)

[Gastrointestinal event 68](#_Toc220619066)

[Guillain-Barré syndrome (GBS) 68](#_Toc220619067)

[Headache 71](#_Toc220619068)

[Hyperglycemia 72](#_Toc220619069)

[Immune thrombocytopenia/idiopathic thrombocytopenic purpura (ITP) 73](#_Toc220619070)

[Induration 73](#_Toc220619071)

[Lethargy 74](#_Toc220619072)

[Lymphadenopathy 74](#_Toc220619073)

[Mobility (reduced) 74](#_Toc220619074)

[Myalgia 75](#_Toc220619075)

[Myocardial infarction 76](#_Toc220619076)

[Nausea 76](#_Toc220619077)

[Nodule 77](#_Toc220619078)

[Pain 77](#_Toc220619079)

[Pain in extremity 78](#_Toc220619080)

[Pericarditis 78](#_Toc220619081)

[Pneumonia 79](#_Toc220619082)

[Pruritus (generalised) 79](#_Toc220619083)

[Pulmonary embolism 79](#_Toc220619084)

[Rash 80](#_Toc220619085)

[RSV infection 81](#_Toc220619086)

[Sepsis, bacteremia, or both 81](#_Toc220619087)

[Shoulder pain 81](#_Toc220619088)

[Skin swelling 82](#_Toc220619089)

[Sleep disorder 82](#_Toc220619090)

[Stroke or transient ischemic attack 82](#_Toc220619091)

[Syncope 83](#_Toc220619092)

[Tenderness 83](#_Toc220619093)

[Thromboembolic event 84](#_Toc220619094)

[Transverse myelitis 84](#_Toc220619095)

[Vomiting 85](#_Toc220619096)

[Other 85](#_Toc220619097)

[Publication bias assessment 87](#_Toc220619098)

[References 88](#_Toc220619099)

# Supplement 1: Search strategies

Table S1. Search strategy for Ovid Embase (Embase Classic+Embase <1947 to 2025 August 05>)

| 1 | exp Human respiratory syncytial virus/ |
| --- | --- |
| 2 | (Human respiratory syncytial virus or respiratory syncytial virus or RSV).ti,ab,kf. |
| 3 | 1 or 2 |
| 4 | exp respiratory syncytial virus vaccine/ |
| 5 | exp vaccine/ |
| 6 | exp vaccination/ or exp immunization/ |
| 7 | (vaccin* or innoculat$ or jab or immuni#tion* or Nirsevimab or Monoclonal antibod* or PF?06928316 or RSVpreF or Prophyla* or Arexvy or Abrysvo or Mresvia).mp. |
| 8 | 4 or 5 or 6 or 7 |
| 9 | exp drug safety/ |
| 10 | exp adverse event/ or exp drug monitoring/ or exp adverse drug reaction/ |
| 11 | ((adverse or side) adj2 (effect* or reaction* or event*)).mp. |
| 12 | (safety or harm* or disadvantage*).mp. |
| 13 | exp clinical effectiveness/ |
| 14 | exp drug efficacy/ |
| 15 | (effect* or efficac* or benefit* or advantage*).mp. |
| 16 | 9 or 10 or 11 or 12 or 13 or 14 or 15 |
| 17 | 3 and 8 and 16 |
| 18 | 17 not ((exp animal/ or nonhuman/) not exp human/) |
| 19 | 18 and (202212* or 2023* or 2024*).dd. |

| 1 | exp Respiratory Syncytial Virus, Human/ |
| --- | --- |
| 2 | (Human respiratory syncytial virus or respiratory syncytial virus or RSV).ti,ab,kf. |
| 3 | 1 or 2 |
| 4 | Viral Vaccines/ |
| 5 | exp Immunization/ |
| 6 | (vaccin* or innoculat$ or jab or immuni#tion* or Nirsevimab or Monoclonal antibod* or PF?06928316 or RSVpreF or Prophyla* or Arexvy or Abrysvo or Mresvia).mp. |
| 7 | 4 or 5 or 6 |
| 8 | exp "Drug-Related Side Effects and Adverse Reactions"/ or exp Adverse Drug Reaction Reporting Systems/ |
| 9 | exp Safety/ |
| 10 | ((adverse or side) adj2 (effect* or reaction* or event*)).mp. |
| 11 | (safety or harm* or disadvantage*).mp. |
| 12 | Comparative Effectiveness Research/ |
| 13 | exp Vaccine Efficacy/ |
| 14 | (effect* or efficac* or benefit* or advantage*).mp. |
| 15 | 8 or 9 or 10 or 11 or 12 or 13 or 14 |
| 16 | 3 and 7 and 15 |
| 17 | exp animals/ not humans.sh. |
| 18 | 16 not 17 |
| 19 | 18 and (202212* or 2023* or 2024*).ed. |

Table S2. Search strategy for Ovid Medline (Ovid MEDLINE(R) ALL <1946 to August 05, 2025>)

Table S3. Search strategy for Global Health database (<1973 to 2025 Week 32>)

| 1 | (Human respiratory syncytial virus or respiratory syncytial virus or RSV).ti,ab,hw. |
| --- | --- |
| 2 | exp vaccines/ |
| 3 | exp immunization/ |
| 4 | (vaccin* or innoculat$ or jab or immuni#tion* or Nirsevimab or Monoclonal antibod* or PF?06928316 or RSVpreF or Prophyla* or Arexvy or Abrysvo or Mresvia).mp. |
| 5 | 2 or 3 or 4 |
| 6 | exp adverse effects/ |
| 7 | ((adverse or side) adj2 (effect* or reaction* or event*)).mp. |
| 8 | (safety or harm* or disadvantage*).mp. |
| 9 | exp efficacy/ |
| 10 | (effectiveness or efficac* or benefit* or advantage*).mp. |
| 11 | 6 or 7 or 8 or 9 or 10 |
| 12 | 1 and 5 and 11 |
| 13 | 12 and (202212* or 2023* or 2024*).dp. |

# Supplement 2: Study selection criteria

Table S4. Study selection criteria

|  | **Inclusion criteria** | **Exclusion criteria** |
| --- | --- | --- |
| Population | Adults aged 60 years and older of any sex and ethnic/racial group, with or without comorbidities | Pregnant people receiving RSV maternal vaccine intended to prevent severe RSV-associated disease in infants |
| Intervention | A single dose of an RSV vaccine (Abrysvo, Pfizer; Arexvy, GSK) | RSV monoclonal antibodies |
| Comparison | Not applicable | Not applicable |
| Outcome | Uptake of RSV vaccines (% of immunised individuals within an eligible population); RSV vaccine effectiveness in preventing RSV-associated healthcare utilisation; population-level impact of RSV vaccines; RSV vaccine safety signals (adverse events following vaccination) | Vaccine efficacy and safety signals reported in clinical trials; reports of RSV antibody titres after vaccination |
| Study design | Observational studies reporting primary real-world data, e.g. case-control studies, cohort studies, case series, quasi-experimental studies, cross-sectional studies; pre-prints were included | Clinical trials, modelling studies, cost-effectiveness evaluations, animal studies, systematic reviews, reports from public health agencies or governmental organisations (grey literature) |
| Language | English | Not English |
| Setting | All real-world settings (community, primary care, secondary care) | None |

# Supplement 3: List of excluded studies

| **Reason** | **Study** |
| --- | --- |
| **Data on RSV maternal vaccine or nirsevimab** | 1. Abraham et al. (2024)^1^ 2. Aguera et al. (2024)^2^ 3. Agyeman et al. (2025)^3^ 4. Ahmed et al. (2025)^4^ 5. Alami et al. (2025)^5^ 6. Alejandre et al. (2024)^6^ 7. Andina Martinez et al. (2024)^7^ 8. Andina Martinez et al. (2025)^8^ 9. Ares-Gomez et al. (2024)^9^ 10. Arico et al. (2025)^10^ 11. Assad et al. (2024)^11^ 12. Attaianese et al. (2025)^12^ 13. Baldenweg et al. (2025)^3^ 14. Barbas Del Buey et al. (2024)^13^ 15. Bermúdez-Barrezueta et al. (2025)^14^ 16. Blauvelt C.A. et al. (2025)^15^ 17. Bloomfield et al. (2025)^16^ 18. Bonnel et al. (2025)^17^ 19. Boundy et al. (2025)^18^ 20. Boutin et al. (2025)^19^ 21. Bracaloni et al. (2024)^20^ 22. Cantais et al. (2024)^21^ 23. Carazo et al. (2025)^22^ 24. Carbajal et al. (2024)^23^ 25. Carcione et al. (2025)^24^ 26. Chauvel et al. (2024)^25^ 27. Cocchi et al. (2025)^26^ 28. Coma et al. (2024)^27^ 29. Coma et al. (2025)^28^ 30. Coma et al. (2025)^29^ 31. Consolati et al. (2024)^30^ 32. Copi et al. (2025)^31^ 33. Costantino et al. (2025)^32^ 34. Creus-Costa et al. (2025)^33^ 35. Dahly et al. (2025)^34^ 36. de Sentuary et al. (2025)^35^ 37. Deck et al. (2025)^36^ 38. DeSilva et al. (2025)^37^ 39. Dong et al. (2025)^38^ 40. Ernst et al. (2024)^39^ 41. Espeleta-Fox et al. (2024)^40^ 42. Estrella-Porter et al. (2024)^41^ 43. Estrella-Porter et al. (2025)^42^ 44. Ezpeleta et al. (2024)^43^ 45. Fafi et al. (2025)^44^ 46. Feitosa et al. (2025)^45^ 47. Fortunato et al. (2025)^46^ 48. Furgier et al. (2025)^47^ 49. Fusco et al. (2025)^48^ 50. Gabet et al. (2025)^49^ 51. Garcia-Garcia et al. (2025)^50^ 52. Garcia Acevedo et al. (2025)^51^ 53. Gentile et al. (2025)^52^ 54. Gentile et al. (2025)^53^ 55. Gonzalez-Bertolin et al. (2025)^54^ 56. Grahic-Mujcinovic et al. (2024)^55^ 57. Gregori-García et al. (2025)^56^ 58. Guerrero-del-Cueto et al. (2025)^57^ 59. Hammitt et al. (2025)^58^ 60. Helwig et al. (2025)^59^ 61. Höck et al. (2025)^60^ 62. Homo et al. (2024)^61^ 63. Homo et al. (2025)^62^ 64. Hsiao et al. (2025)^63^ 65. Hsieh et al. (2025)^64^ 66. Irving et al. (2025)^65^ 67. Izquierdo et al. (2025)^66^ 68. Jabagi et al. (2025)^67^ 69. Jesus Perez Martin et al. (2025)^68^ 70. Jeziorski et al. (2025)^69^ 71. Jimeno Ruiz et al. (2024)^70^ 72. Joseph et al. (2025)^71^ 73. Kalya et al. (2025)^72^ 74. Kemp et al. (2025)^73^ 75. Lantigua-Martinez et al. (2025)^74^ 76. Lassoued et al. (2024)^75^ 77. Lastrucci et al. (2025)^76^ 78. Lefferts et al. (2024)^77^ 79. Lenglart et al. (2025)^78^ 80. Lenglart et al. (2025)^79^ 81. Levy et al. (2024)^80^ 82. Lipsett et al. (2025)^81^ 83. Litman et al. (2025)^82^ 84. Lopez-Lacort et al. (2024)^83^ 85. Lopez-Lacort et al. (2024)^84^ 86. Ma et al. (2025)^85^ 87. Mallah et al. (2024)^86^ 88. Manzanares et al. (2025)^87^ 89. Manzoni et al. (2025)^88^ 90. Marouk et al. (2025)^89^ 91. Martinón-Torres et al. (2023)^90^ 92. Mazagatos et al. (2024)^91^ 93. McLachlan et al. (2025)^92^ 94. Mendez-Echevarria et al. (2025)^93^ 95. Molina Gutierrez et al. (2024)^94^ 96. Moline et al. (2024)^95^ 97. Moline et al. (2025)^96^ 98. Moreno-Perez et al. (2025)^97^ 99. Moro et al. (2024)^98^ 100. Moro et al. (2025)^99^ 101. Nguyen et al. (2025)^100^ 102. Nieddu et al. (2025)^101^ 103. Nunez et al. (2025)^102^ 104. Paireau et al. (2024)^103^ 105. Paramo et al. (2025)^104^ 106. Paris et al. (2025)^105^ 107. Pastor-Barriuso et al. (2025)^106^ 108. Patton et al. (2025)^107^ 109. Payne et al. (2025)^108^ 110. Pelletier et al. (2025)^109^ 111. Perez Marc et al. (2025)^110^ 112. Pérez Martín et al. (2024)^111^ 113. Perramon-Malavez et al. (2024)^112^ 114. Perramon-Malavez et al. (2025)^113^ 115. Perramon-Malavez et al. (2025)^114^ 116. Petat et al. (2025)^115^ 117. Puckett et al. (2024)^116^ 118. Raguz et al. (2022)^117^ 119. Razai et al. (2025)^118^ 120. Razzaghi et al. (2024)^119^ 121. Reina et al. (2024)^120^ 122. Remmele et al. (2024)^121^ 123. Rius-Peris et al. (2025)^122^ 124. Schaffer De Roo et al. (2025)^123^ 125. Scruzzi et al. (2025)^124^ 126. Shedlock et al. (2025)^125^ 127. Silva-Afonso et al. (2025)^126^ 128. Sindy et al. (2025)^3^ 129. Somers et al. (2025)^127^ 130. Son et al. (2024)^128^ 131. Steinberg et al. (2025)^129^ 132. Steinberg et al. (2025)^3^ 133. Torres et al. (2025)^130^ 134. Touati et al. (2025)^131^ 135. Vazquez-Lopez et al. (2025)^132^ 136. Veyrenche et al. (2025)^133^ 137. Villani et al. (2025)^134^ 138. Wadia et al. (2025)^135^ 139. Wilcox et al. (2025)^136^ 140. Williams et al. (2025)^137^ 141. Williams et al. (2025)^138^ 142. Xu et al. (2024)^139^ 143. Xu et al. (2025)^140^ 144. Yan et al. (2025)^141^ 145. Zhou et al. (2025)^142^ |
| **No full text available (e.g. abstracts, editorials)** | 1. Albar et al. (2025)^143^ 2. Anonymous^144^ 3. Blauvelt, C.A. et al. (2024)^145^ 4. Carino et al. (2025)^146^ 5. Creus-Costa et al. (2025)^147^ 6. Georgiadis et al. (2024)^148^ 7. Green et al. (2025)^149^ 8. Haddad et al. (2025)^150^ 9. Hamid et al. (2024)^151^ 10. Hsiao et al. (2024)^152^ 11. Jacobson et al. (2025)^153^ 12. Jawad et al. (2025)^154^ 13. La et al. (2024)^155^ 14. Lai et al. (2023)^156^ 15. Loeb et al. (2024)^157^ 16. Lorenzini et al. (2023) 17. Lupton et al. (2025)^158^ 18. MacDonald et al. (2025)^159^ 19. Mann et al. (2025)^160^ 20. Martin et al. (2024)^161^ 21. Molnar et al. (2023)^162^ 22. Morris et al. (2025)^163^ 23. Rallabhandi et al. (2024)^164^ 24. Ransohoff et al. (2025)^165^ 25. Reich et al. (2025)^166^ 26. Shelley et al. (2025)^167^ 27. Simeone et al. (2025)^168^ 28. Smith-Jeffcoat et al. (2025)^169^ 29. Tartof et al. (2025)^170^ 30. Torres et al. (2025)^171^ 31. Trivedi et al. (2025)^172^ 32. Verheust et al. (2025)^173^ |
| **No uptake, effectiveness, or safety data** | 1. Alami et al. (2024)^174^ 2. Caillault et al. (2025)^175^ 3. Domnich et al. (2025)^176^ 4. Falsey et al. (2024)^177^ 5. Fourati et al. (2025)^178^ 6. Gaffney et al. (2025)^179^ 7. Gratzl et al. (2024)^180^ 8. Hannaford et al. (2025)^181^ 9. Jasseh et al. (2025)^182^ 10. Jasset et al. (2025)^183^ 11. Kalampokini et al. (2025)^184^ 12. Karaba et al. (2025)^185^ 13. Machida et al. (2025)^186^ 14. Mestre-Ferrandiz et al. (2024)^187^ 15. Murray et al. (2025)^188^ 16. Nyiro et al. (2022)^189^ 17. Seby et al. (2025)^190^ 18. Wei et al. (2025)^191^ 19. Wetzke et al. (2025)^192^ |
| **No real-world evidence** | 1. Adhikari et al. (2024)^193^ 2. Bowe et al. (2025)^194^ 3. Brault et al. (2024)^195^ 4. Du et al. (2025)^196^ 5. Hansen et al. (2025)^197^ 6. Maculaitis et al. (2024)^198^ 7. Marcellusi et al. (2025)^199^ 8. Sallam et al. (2025)^200^ 9. Saure et al. (2025)^201^ 10. Trubin et al. (2024)^202^ 11. Trusinska et al. (2025)^203^ 12. Verelst et al. (2025)^204^ 13. Wang et al. (2025)^205^ |
| **Clinical trial** | 1. Biegus et al. (2024)^206^ 2. Domachowske et al. (2022)^207^ 3. Domachowske et al. (2023)^208^ 4. Ilangovan et al. (2025)^209^ 5. Walsh et al. (2025)^210^ 6. Wilson et al. (2023)^211^ |
| **Studies not in English** | 1. Andina Martinez et al. (2025)^212^ 2. Matt et al. (2025)^213^ 3. Novoa Pizarro et al. (2023)^214^ 4. Rodriguez-Fernandez et al. (2024)^215^ 5. Scruzzi et al. (2025)^216^ 6. Vera-Punzano et al. (2025)^217^ |

# Supplement 4: ‘Risk-of-bias’ quality assessment

To assess the risk of bias, we used Joanna Briggs Institute (JBI) Critical Appraisal Tools for cohort studies, case control studies, cross-sectional studies, quasi-experimental studies, or case series depending on the study designs.^218^

‘Risk of bias’ assessments were completed by two reviewers for each study, marking each criterion as ‘yes’, ‘no’, ‘unclear’, or ‘not applicable’. Conflicts were resolved in a discussion. The number of ‘yes’ responses for each study (total) were divided by the total number of criteria to obtain a score ranging from 0% to 100%. Studies with scores 50% and less were classified as ‘high risk’, scores between 51% and 75% were considered ‘medium risk’, and studies with scores above 75% were considered ‘low risk’.

Sensitivity analyses of the pooled estimates were conducted using data from studies classified as at ‘low risk of bias’. This sensitivity analysis could not be carried out for all meta-estimates because less than three studies reporting the relevant data were classified as at ‘low risk of bias’.

**Table S5. ‘Risk of bias’ assessment for cohort studies**

**JBI criteria:**

Q1: Were the two groups similar and recruited from the same population?

Q2: Were the exposures measured similarly to assign people to both exposed and unexposed groups?

Q3: Was the exposure measured in a valid and reliable way?

Q4: Were confounding factors identified?

Q5: Were strategies to deal with confounding factors stated?

Q6: Were the groups/participants free of the outcome at the start of the study (or at the moment of exposure)?

Q7: Were the outcomes measured in a valid and reliable way?

Q8: Was the follow up time reported and sufficient to be long enough for outcomes to occur?

Q9: Was follow up complete, and if not, were the reasons to loss to follow up described and explored?

Q10: Were strategies to address incomplete follow up utilized?

Q11: Was appropriate statistical analysis used?

| **Study** | **Q1** | **Q2** | **Q3** | **Q4** | **Q5** | **Q6** | **Q7** | **Q8** | **Q9** | **Q10** | **Q11** | **Total** | **Total (%)** | **Risk of bias** |
| --- | --- | --- | --- | --- | --- | --- | --- | --- | --- | --- | --- | --- | --- | --- |
| Bajema et al. (2025)^219^ | Yes | Yes | Yes | Yes | Yes | Yes | Yes | Yes | Yes | Yes | Yes | 11 | 100.0% | low |
| Birabaharan et al. (2024)^220^ | Yes | Yes | Yes | Yes | Yes | Yes | Yes | Yes | Yes | Yes | Yes | 11 | 100.0% | low |
| Domnich et al. (2025)^221^ | Yes | Not applicable | Yes | Yes | Yes | Yes | No | Yes | Unclear | No | Yes | 7 | 63.6% | medium |
| Donahue et al. (2025)^222^ | Yes | Yes | Yes | Yes | Yes | Unclear | Yes | Yes | Unclear | Unclear | Yes | 8 | 72.7% | medium |
| Godonou et al. (2025)^223^ | Yes | Yes | Yes | Yes | Yes | Yes | Yes | Yes | Unclear | Unclear | Yes | 9 | 81.8% | low |
| Hall et al. (2025)^224^ | Yes | Yes | Yes | No | Not applicable | Yes | Yes | Yes | Unclear | No | Yes | 7 | 63.6% | medium |
| Hause et al. (2024)^225^ | Yes | Yes | No | No | No | No | Unclear | Yes | Unclear | Unclear | No | 3 | 27.3% | high |
| Havlin et al. (2025)^226^ | Yes | Yes | Yes | Yes | No | Yes | No | Yes | Yes | Not applicable | Yes | 8 | 72.7% | medium |
| Kim et al. (2025)^227^ | Yes | Yes | Yes | Yes | Yes | Yes | Yes | Yes | Not applicable | Not applicable | Yes | 9 | 81.8% | low |
| Levy et al. (2025)^228^ | Not applicable | Not applicable | Yes | Unclear | Unclear | Yes | Yes | Unclear | Yes | Not applicable | Unclear | 4 | 36.4% | high |
| Lotscher et al. (2025)^229^ | Not applicable | Not applicable | Yes | No | No | Yes | Yes | Yes | Yes | No | Unclear | 5 | 45.5% | high |
| Murphy et al. (2025)^230^ | Yes | Yes | Yes | No | No | Not applicable | Yes | Yes | Not applicable | Not applicable | Yes | 6 | 54.5% | medium |
| Patrick et al. (2025)^231^ | Yes | Yes | Yes | Yes | Yes | Yes | Yes | Yes | Yes | Yes | Yes | 11 | 100.0% | low |
| Redjoul et al. (2025)^232^ | Not applicable | Not applicable | Yes | No | No | Yes | Yes | Unclear | Yes | Not applicable | Unclear | 4 | 36.4% | high |
| Reses et al. (2023)^233^ | Yes | Yes | Yes | No | No | No | Yes | Unclear | No | No | Yes | 5 | 45.5% | high |
| Reses et al. (2024)^234^ | Yes | Yes | Yes | No | No | No | Yes | Unclear | No | No | Yes | 5 | 45.5% | high |

**Table S6. ‘Risk of bias’ assessment for cross-sectional studies**

**JBI criteria:**

Q1: Were the criteria for inclusion in the sample clearly defined?

Q2: Were the study subjects and the setting described in detail?

Q3: Was the exposure measured in a valid and reliable way?

Q4: Were objective, standard criteria used for measurement of the condition?

Q5: Were confounding factors identified?

Q6: Were strategies to deal with confounding factors stated?

Q7: Were the outcomes measured in a valid and reliable way?

Q8: Was appropriate statistical analysis used?

| **Study** | **Q1** | **Q2** | **Q3** | **Q4** | **Q5** | **Q6** | **Q7** | **Q8** | **Total** | **Total (%)** | **Risk of bias** |
| --- | --- | --- | --- | --- | --- | --- | --- | --- | --- | --- | --- |
| Bao et al. (2025)^235^ | Yes | Yes | Yes | Unclear | No | No | Unclear | Unclear | 3 | 37.5% | high |
| Geng et al. (2024)^236^ | Yes | Yes | No | Unclear | Yes | Yes | Yes | Yes | 6 | 75.0% | medium |
| La et al. (2025)^237^ | Yes | Yes | Yes | Yes | Not applicable | Not applicable | Yes | Yes | 6 | 75.0% | low |
| Li et al. (2025)^238^ | Yes | Yes | Yes | Yes | Unclear | Unclear | Yes | Unclear | 5 | 62.5% | medium |
| Morrison et al. (2025)^239^ | Yes | No | Yes | Unclear | No | No | Yes | Yes | 4 | 50.0% | medium |
| Motta et al. (2025)^240^ | Yes | Unclear | No | Unclear | Yes | Yes | No | Yes | 4 | 50.0% | high |
| Nguyen et al. (2025)^241^ | Yes | Yes | Yes | No | No | No | No | Yes | 4 | 50.0% | medium |
| Rizzo et al. (2025)^242^ | Yes | Yes | Yes | Yes | Yes | Unclear | Unclear | Yes | 6 | 75.0% | low |
| Surie et al. (2025a)^243^ | Yes | Yes | Yes | Yes | Yes | Yes | Yes | Yes | 8 | 100.0% | low |
| Viskupic et al. (2025)^244^ | Yes | No | Unclear | Unclear | Yes | Yes | Unclear | Yes | 4 | 50.0% | medium |

**Table S7. ‘Risk of bias’ assessment for case-control studies**

**JBI criteria:**

Q1: Were the groups comparable other than the presence of disease in cases or the absence of disease in controls?

Q2: Were cases and controls matched appropriately?

Q3: Were the same criteria used for identification of cases and controls?

Q4: Was exposure measured in a standard, valid and reliable way?

Q5: Was exposure measured in the same way for cases and controls?

Q6: Were confounding factors identified?

Q7: Were strategies to deal with confounding factors stated?

Q8: Were outcomes assessed in a standard, valid and reliable way for cases and controls?

Q9: Was the exposure period of interest long enough to be meaningful?

Q10: Was appropriate statistical analysis used?

| **Study** | **Q1** | **Q2** | **Q3** | **Q4** | **Q5** | **Q6** | **Q7** | **Q8** | **Q9** | **Q10** | **Total** | **Total (%)** | **Risk of bias** |
| --- | --- | --- | --- | --- | --- | --- | --- | --- | --- | --- | --- | --- | --- |
| Fry et al. (2025)^245^ – vaccine effectiveness study | Yes | Not applicable | Yes | Yes | Yes | Unclear | No | Yes | Yes | No | 6 | 60.0% | medium |
| Payne et al. (2024)^246^ | Yes | No | Yes | Yes | Yes | Yes | Yes | Yes | Yes | Yes | 9 | 90.0% | low |
| Surie et al. (2024)^247^ | Yes | Yes | Yes | Yes | Yes | Yes | Yes | Yes | Unclear | Yes | 9 | 90.0% | low |
| Surie et al. (2025b)^243^ | Yes | Yes | Yes | Yes | Yes | Yes | Yes | Yes | Unclear | Yes | 9 | 90.0% | low |
| Symes et al. (2025) ^248^ | Yes | Yes | Yes | Yes | Yes | Yes | Yes | Yes | Yes | Yes | 10 | 100.0% | low |
| Tartof et al. (2024)^249^ | Yes | No | Yes | Yes | Yes | Yes | Yes | Yes | Unclear | Yes | 8 | 80.0% | low |
| Tartof et al. (2025)^250^ | Yes | No | Yes | Yes | Yes | Yes | Yes | Yes | Unclear | Yes | 8 | 80.0% | low |

**Table S8. ‘Risk of bias’ assessment for quasi-experimental studies (incl. regression discontinuity design studies)**

**JBI criteria:**

Q1: Is it clear in the study what is the “cause” and what is the “effect” (i.e. there is no confusion about which variable comes first)?

Q2: Was there a control group?

Q3: Were participants included in any comparisons similar?

Q4: Were the participants included in any comparisons receiving similar treatment/care, other than the exposure or intervention of interest?

Q5: Were there multiple measurements of the outcome, both pre and post the intervention/exposure?

Q6: Were the outcomes of participants included in any comparisons measured in the same way?

Q7: Were outcomes measured in a reliable way?

Q8: Was follow-up complete and if not, were differences between groups in terms of their follow-up adequately described and analyzed?

Q9: Was appropriate statistical analysis used?

| **Study** | **Q1** | **Q2** | **Q3** | **Q4** | **Q5** | **Q6** | **Q7** | **Q8** | **Q9** | **Total** | **Total (%)** | **Risk of bias** |
| --- | --- | --- | --- | --- | --- | --- | --- | --- | --- | --- | --- | --- |
| Hameed et al. (2025)^251^ | Yes | Yes | Unclear | Unclear | No | Yes | Yes | Unclear | Yes | 5 | 55.6% | medium |
| Mensah et al. (2025)^252^ | Yes | Yes | Unclear | Unclear | No | Yes | Yes | Unclear | Yes | 5 | 55.6% | medium |

**Table S9. ‘Risk of bias’ assessment for case series**

**JBI criteria:**

Q1: Were there clear criteria for inclusion in the case series?

Q2: Was the condition measured in a standard, reliable way for all participants included in the case series?

Q3: Were valid methods used for identification of the condition for all participants included in the case series?

Q4: Did the case series have consecutive inclusion of participants?

Q5: Did the case series have complete inclusion of participants?

Q6: Was there clear reporting of the demographics of the participants in the study?

Q7: Was there clear reporting of clinical information of the participants?

Q8: Were the outcomes or follow up results of cases clearly reported?

Q9: Was there clear reporting of the presenting site(s)/clinic(s) demographic information?

Q10: Was statistical analysis appropriate?

| **Study** | **Q1** | **Q2** | **Q3** | **Q4** | **Q5** | **Q6** | **Q7** | **Q8** | **Q9** | **Q10** | **Total** | **Total (%)** | **Risk of bias** |
| --- | --- | --- | --- | --- | --- | --- | --- | --- | --- | --- | --- | --- | --- |
| Fry et al. (2025)^245^ – vaccine safety | Yes | Yes | Yes | Yes | Unclear | No | Yes | Yes | Yes | Yes | 8 | 80.0% | low |
| Lloyd et al. (2025)^253^ | Yes | Yes | Yes | Unclear | Unclear | Yes | Yes | Yes | Yes | Yes | 8 | 80.0% | low |

# Supplement 5: Sensitivity analysis for RSV vaccine uptake

Sensitivity analyses were carried out using data from studies classified as ‘low risk of bias’ in the risk-of-bias quality assessment (Supplement 3).

Figure S1. Sensitivity analysis of uptake of RSV vaccines among eligible adults aged 60 years and older in the United States


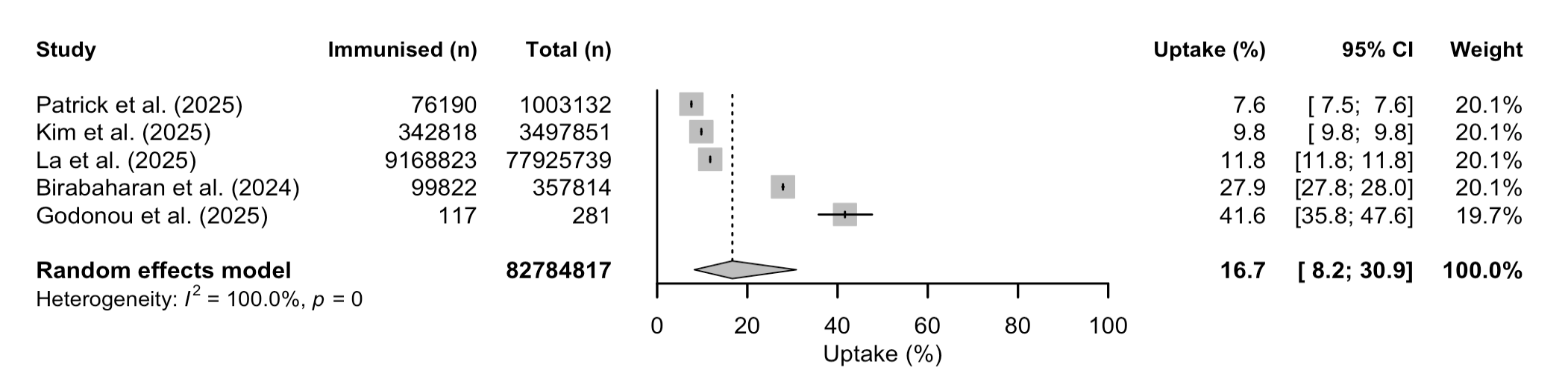


# Supplement 6: RSV vaccine uptake in the United States by subgroup (as reported in Table 2)

Figure S2. **Uptake of RSV vaccines among older adults aged 60 years or older in the United States stratified by age group^a^**


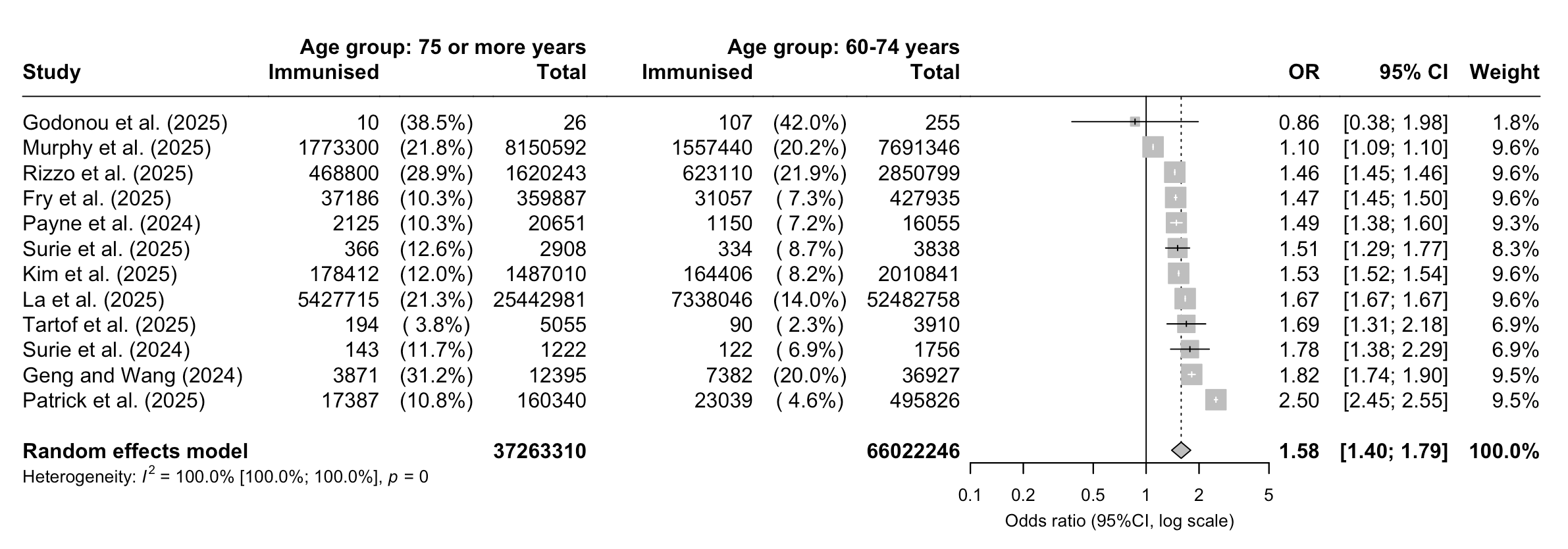


^a^ People 75 or more years old compared to people 60-74 years old (ref.).

Figure S3. **Uptake of RSV vaccines among older adults aged 60 years or older in the United States stratified by age group^a^ (sensitivity analysis – ‘low risk-of-bias’ studies only)**


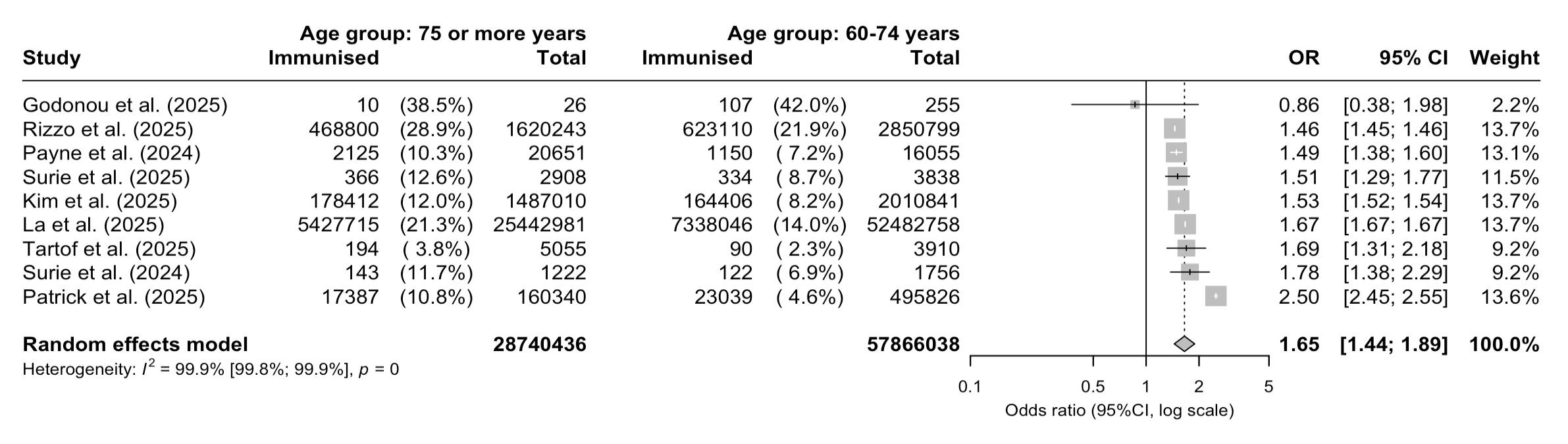


^a^ People 75 or more years old compared to people 60-74 years old (ref.).

**Figure S4. Uptake of RSV vaccines among older adults aged 60 years or older in the United stratified by sex: males and females (ref.).**


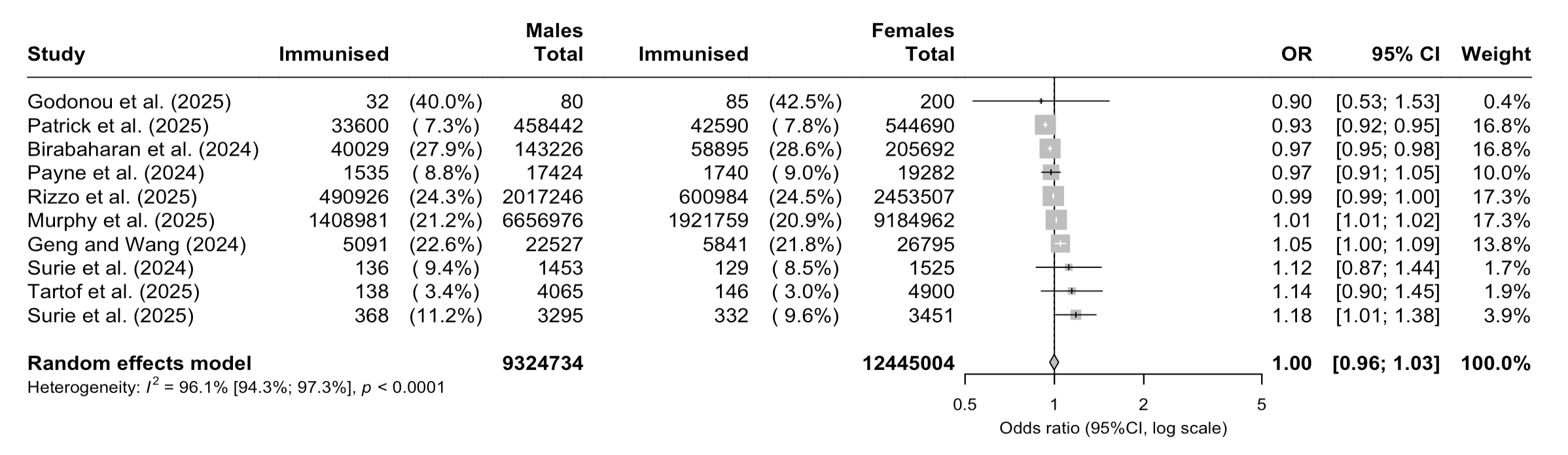


**Figure S5. Uptake of RSV vaccines among older adults aged 60 years or older in the United stratified by sex: males and females (ref.) (sensitivity analysis – ‘low risk-of-bias’ studies only)**


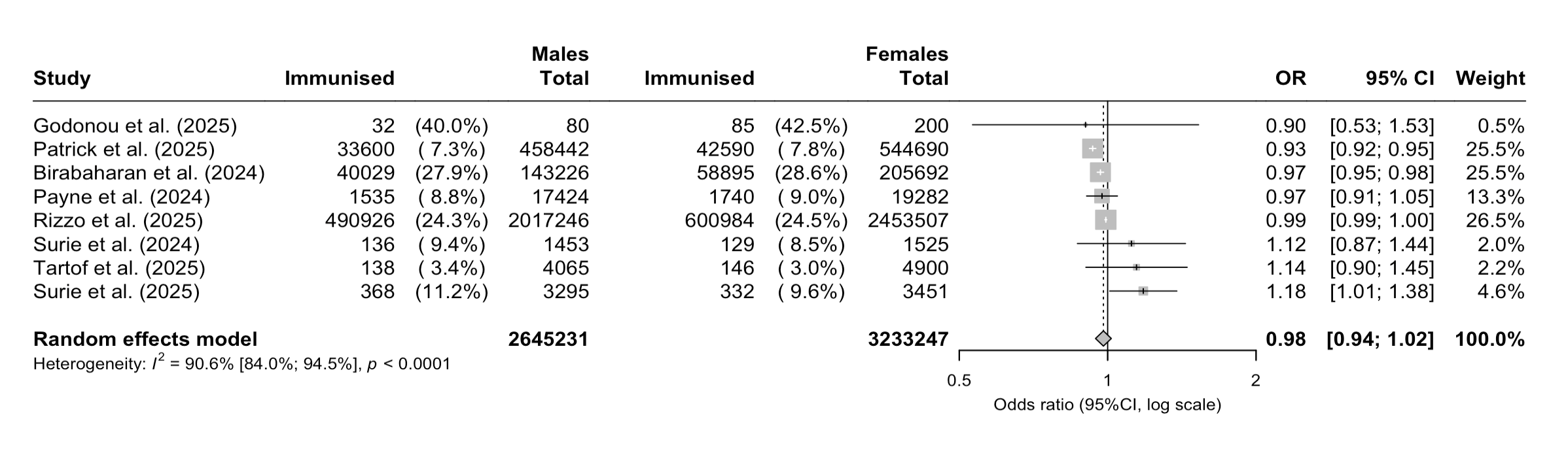


**Figure S6. Uptake of RSV vaccines among eligible older adults aged 60 years or older in the United stratified by presence of comorbidities^a^**


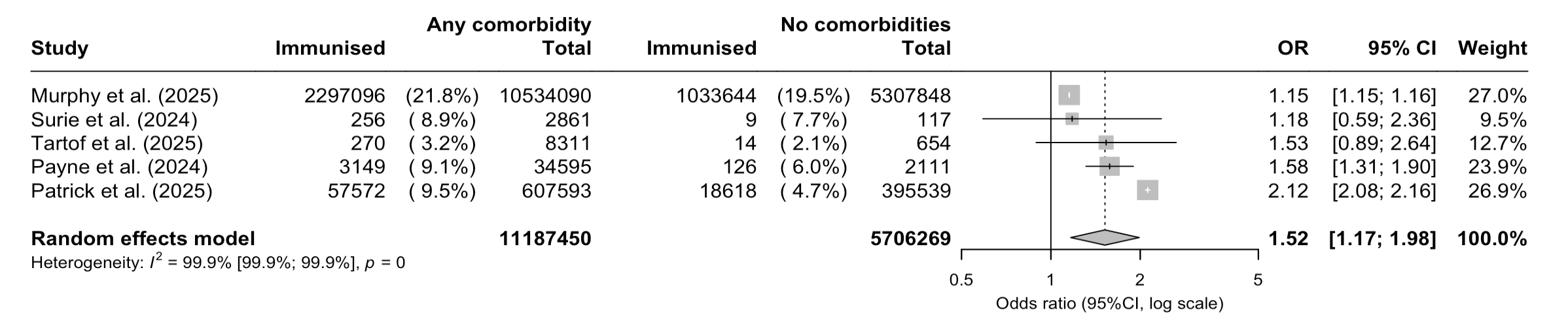


^a^ People with no comorbidities (ref.) compared to people with one or more comorbidities.

**Figure S7. Uptake of RSV vaccines among eligible older adults aged 60 years or older in the United stratified by presence of comorbidities^a^ (sensitivity analysis – ‘low risk-of-bias’ studies only)**


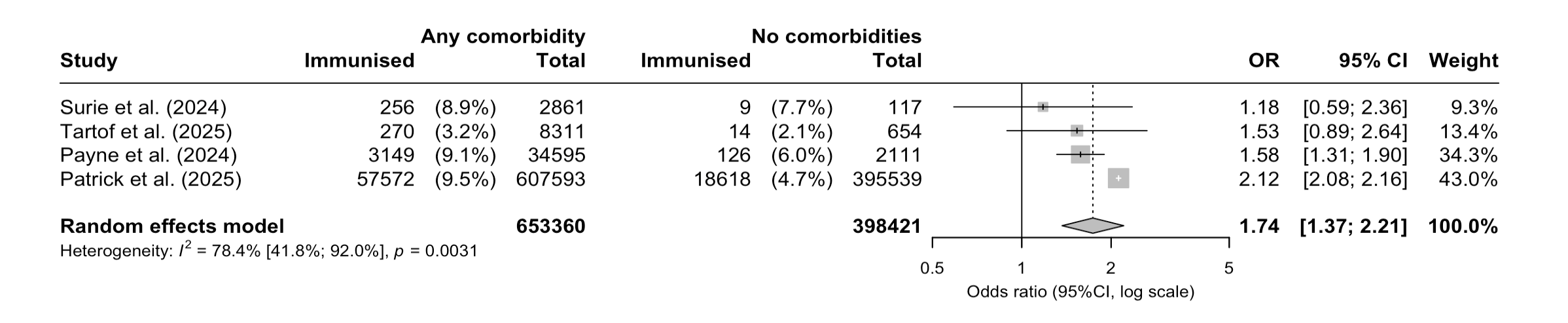


^a^ People with no comorbidities (ref.) compared to people with one or more comorbidities.

**Figure S8. Uptake of RSV vaccines among older adults aged 60 years or older in the United States stratified by immunocompetence^a,b^**


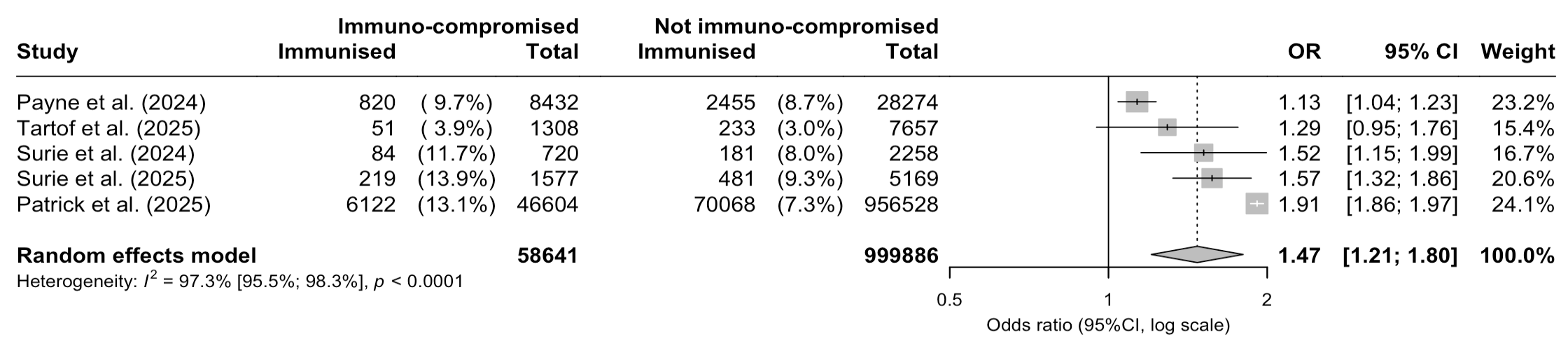


^a^ Immuno-compromised people compared to people without immuno-compromised status.

^b^ In the included studies, immunocompromised status was defined as having an active solid tumour or hematologic malignancy, solid organ transplant hematopoietic cell transplant, HIV infection, primary immunodeficiency, splenectomy, use of immunosuppressive medication in the past 30 days, or other conditions that cause moderate or severe immunosuppression.

**Figure S9. Uptake of RSV vaccines among older adults aged 60 years or older in the United States stratified by immunocompetence^a,b^ (sensitivity analysis – ‘low risk-of-bias’ studies only)**


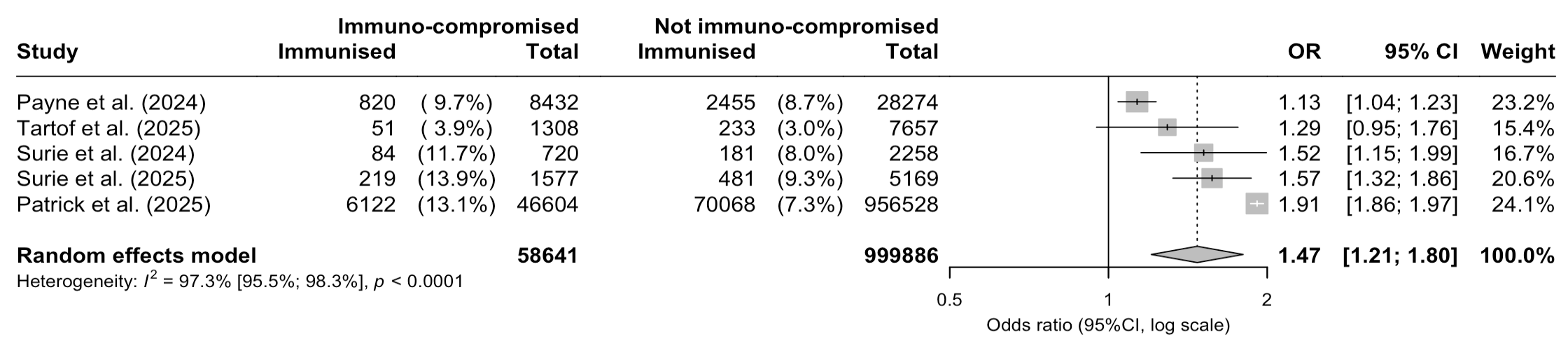


^a^ Immuno-compromised people compared to people without immuno-compromised status.

^b^ In the included studies, immunocompromised status was defined as having an active solid tumour or hematologic malignancy, solid organ transplant hematopoietic cell transplant, HIV infection, primary immunodeficiency, splenectomy, use of immunosuppressive medication in the past 30 days, or other conditions that cause moderate or severe immunosuppression.

**Figure S10. Uptake of RSV vaccines among eligible older adults aged 60 years or older with cardiovascular disease compared to those with no comorbidities in the United States**


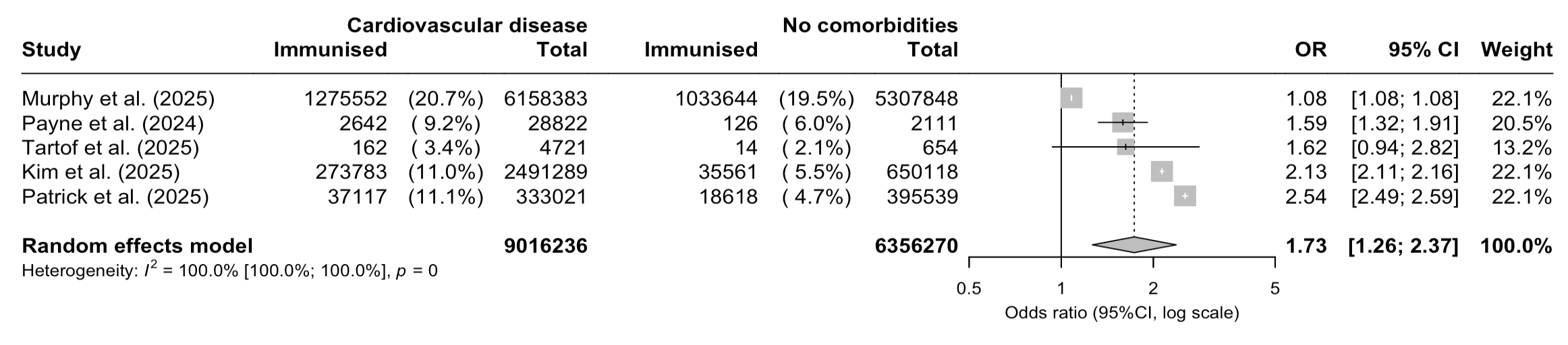


**Figure S10. Uptake of RSV vaccines among eligible older adults aged 60 years or older with cardiovascular disease compared to those with no comorbidities in the United States (sensitivity analysis – ‘low risk-of-bias’ studies only)**


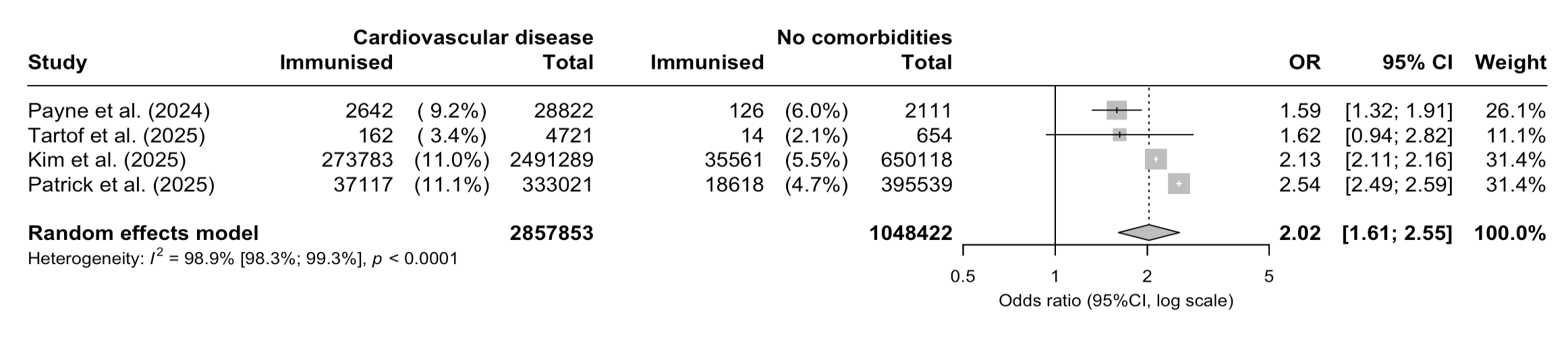


**Figure S11. Uptake of RSV vaccines among eligible older adults aged 60 years or older with metabolic or endocrinologic disease compared to those with no comorbidities in the United States**


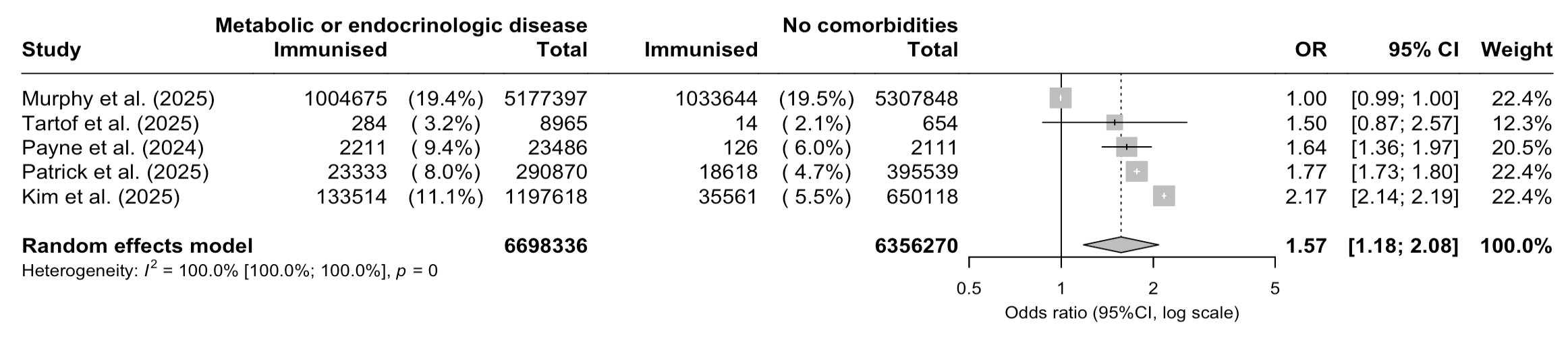


**Figure S12. Uptake of RSV vaccines among eligible older adults aged 60 years or older with metabolic or endocrinologic disease compared to those with no comorbidities in the United States (sensitivity analysis – ‘low risk-of-bias’ studies only)**


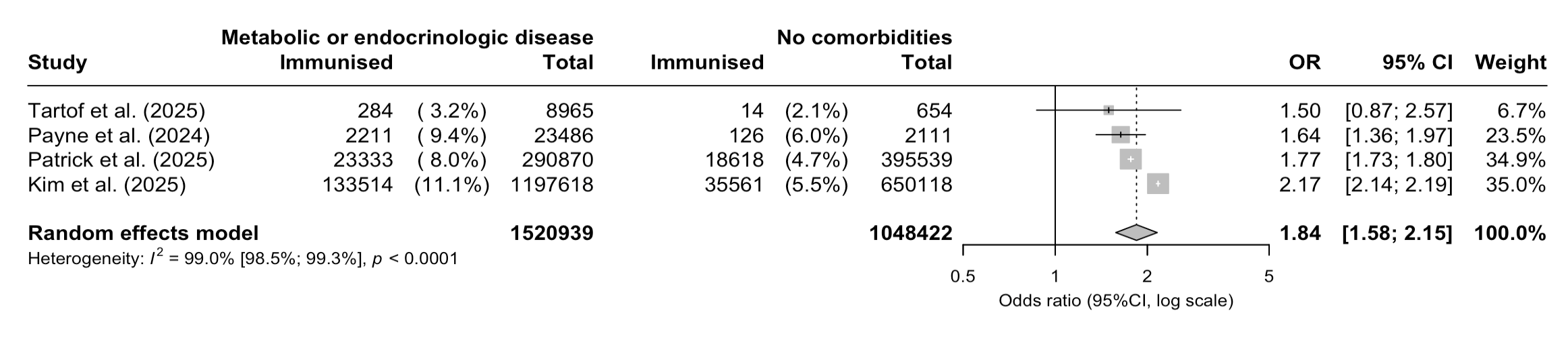


**Figure S13. Uptake of RSV vaccines among eligible older adults aged 60 years or older with kidney disease compared to those with no comorbidities in the United States**


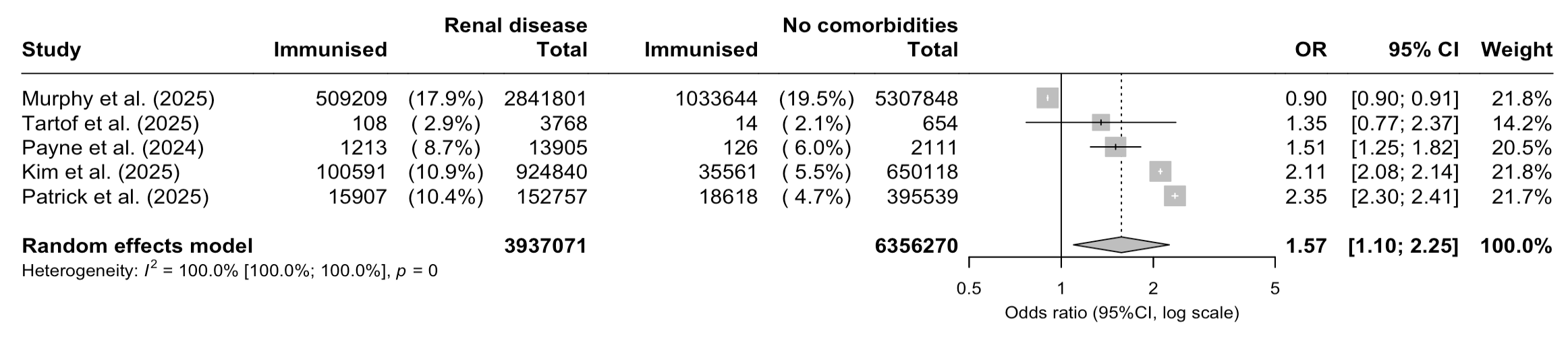


**Figure S14. Uptake of RSV vaccines among eligible older adults aged 60 years or older with kidney disease compared to those with no comorbidities in the United States (sensitivity analysis – ‘low risk-of-bias’ studies only)**


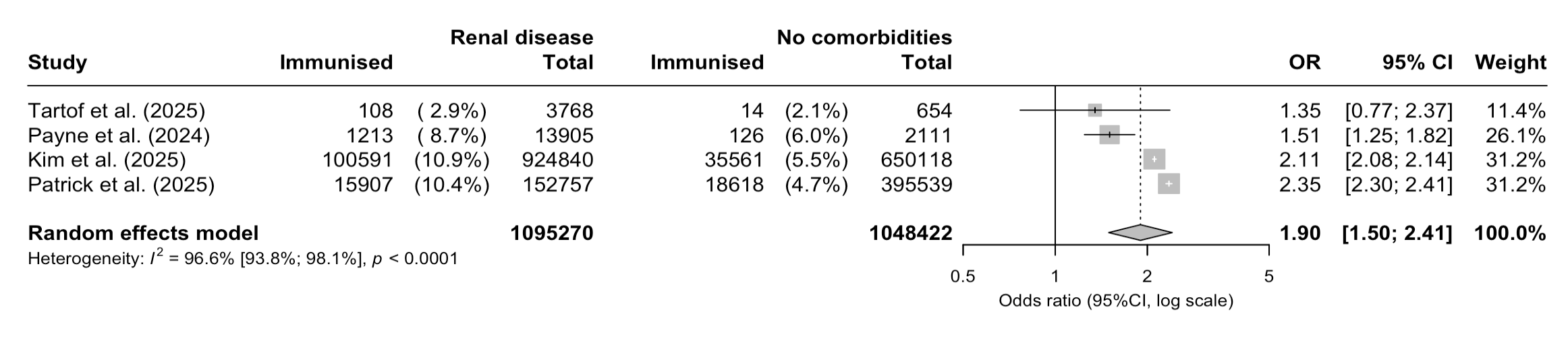


**Figure S15. Uptake of RSV vaccines among eligible older adults aged 60 years or older with lung disease compared to those with no comorbidities in the United States**


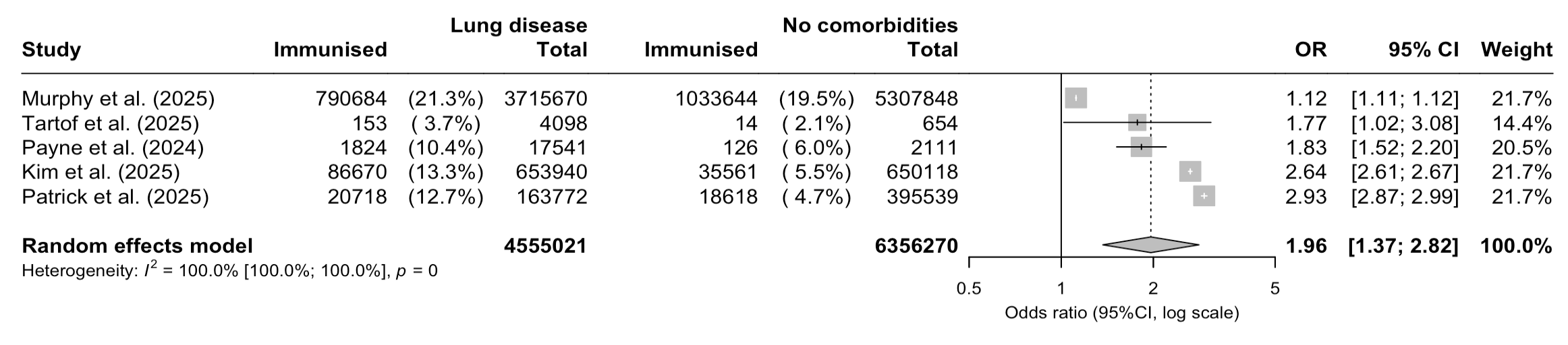


**Figure S16. Uptake of RSV vaccines among eligible older adults aged 60 years or older with lung disease compared to those with no comorbidities in the United States (sensitivity analysis – ‘low risk-of-bias’ studies only)**


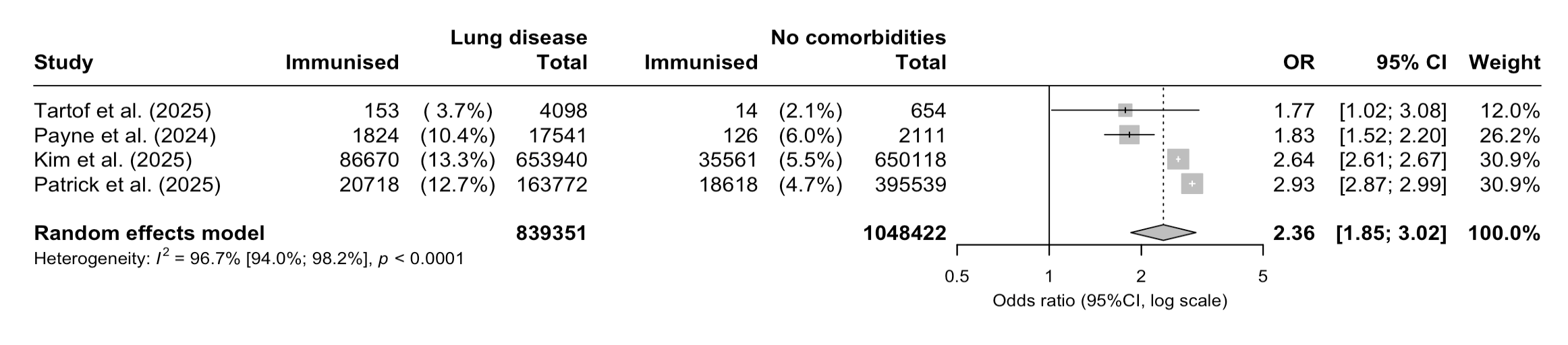


**Figure S17. Uptake of RSV vaccines among eligible older adults aged 60 years or older with one comorbidity compared to those with no comorbidities in the United States**


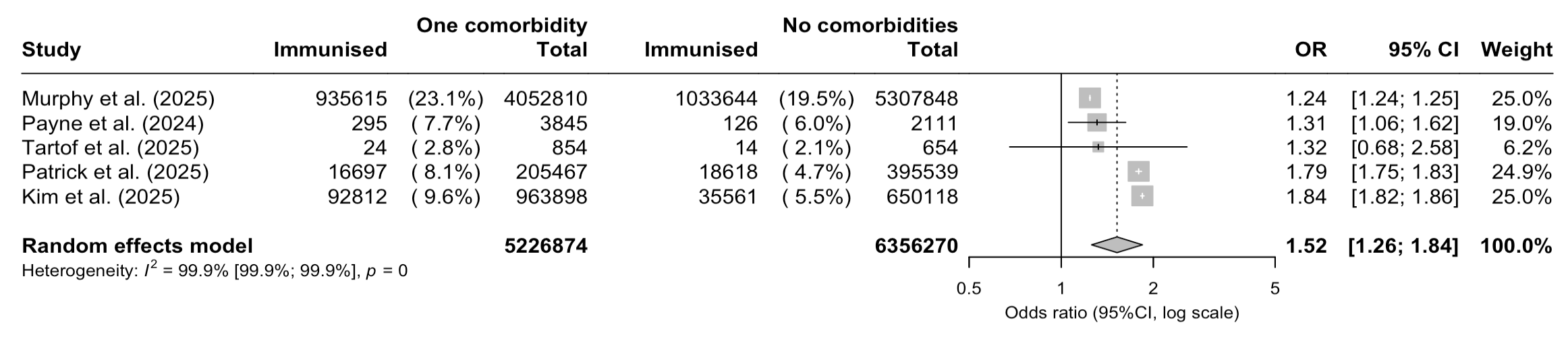


**Figure S18. Uptake of RSV vaccines among eligible older adults aged 60 years or older with one comorbidity compared to those with no comorbidities in the United States (sensitivity analysis – ‘low risk-of-bias’ studies only)**


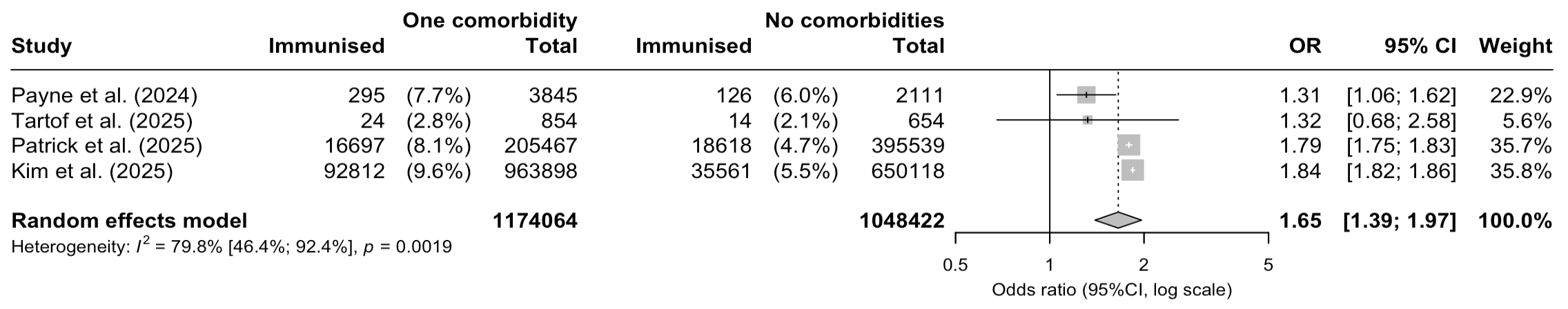


**Figure S19. Uptake of RSV vaccines among eligible older adults aged 60 years or older with two or more comorbidities compared to those with no comorbidities in the United States**


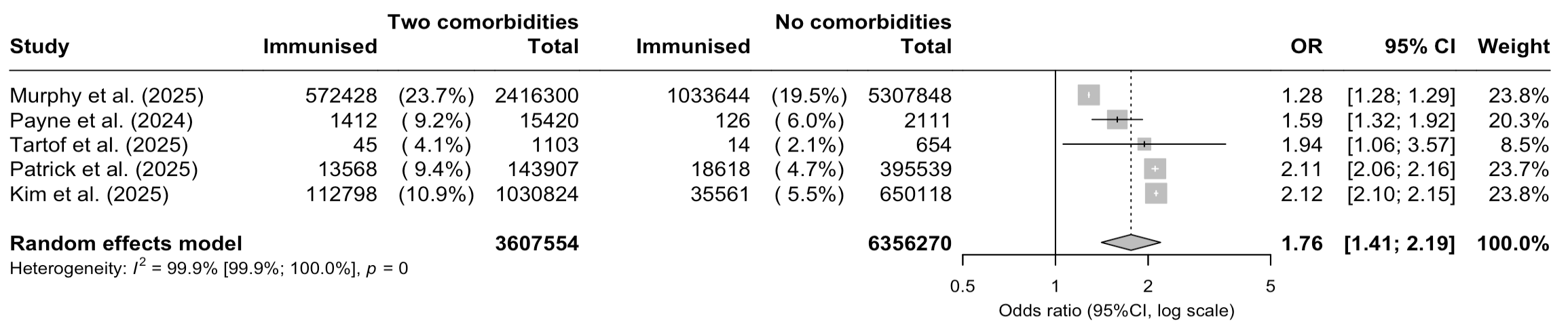


**Figure S20. Uptake of RSV vaccines among eligible older adults aged 60 years or older with two or more comorbidities compared to those with no comorbidities in the United States (sensitivity analysis – ‘low risk-of-bias’ studies only)**


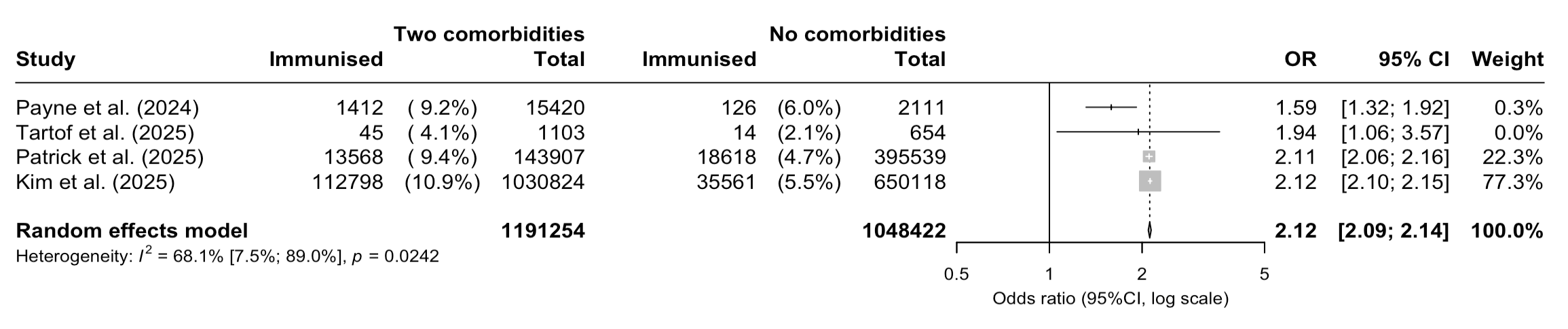


**Figure S21. Uptake of RSV vaccines among eligible older adults aged 60 years or older with two comorbidities compared to those with one comorbidity in the United States**


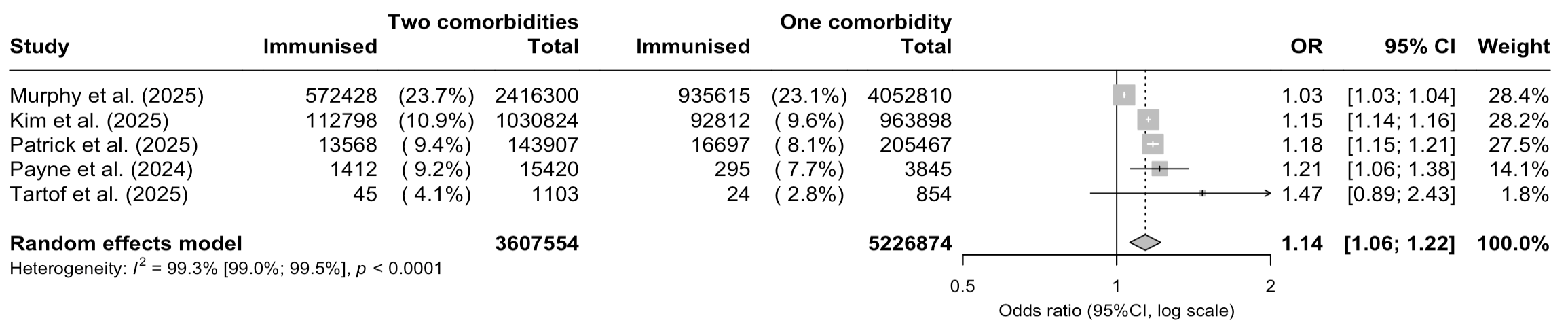


**Figure S22. Uptake of RSV vaccines among eligible older adults aged 60 years or older with two comorbidities compared to those with one comorbidity in the United States (sensitivity analysis – ‘low risk-of-bias’ studies only)**


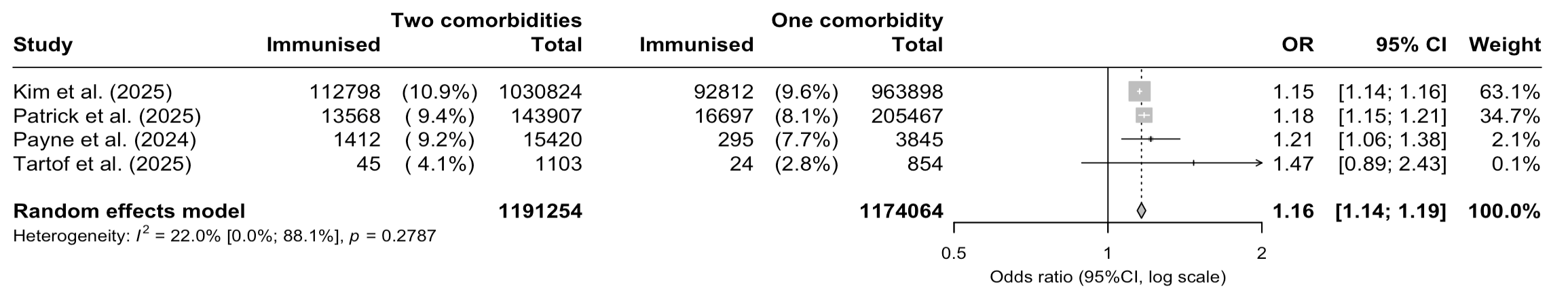


**Figure S23. Uptake of RSV vaccines among eligible older adults aged 60 years or older in the United States stratified by racial group^a^**


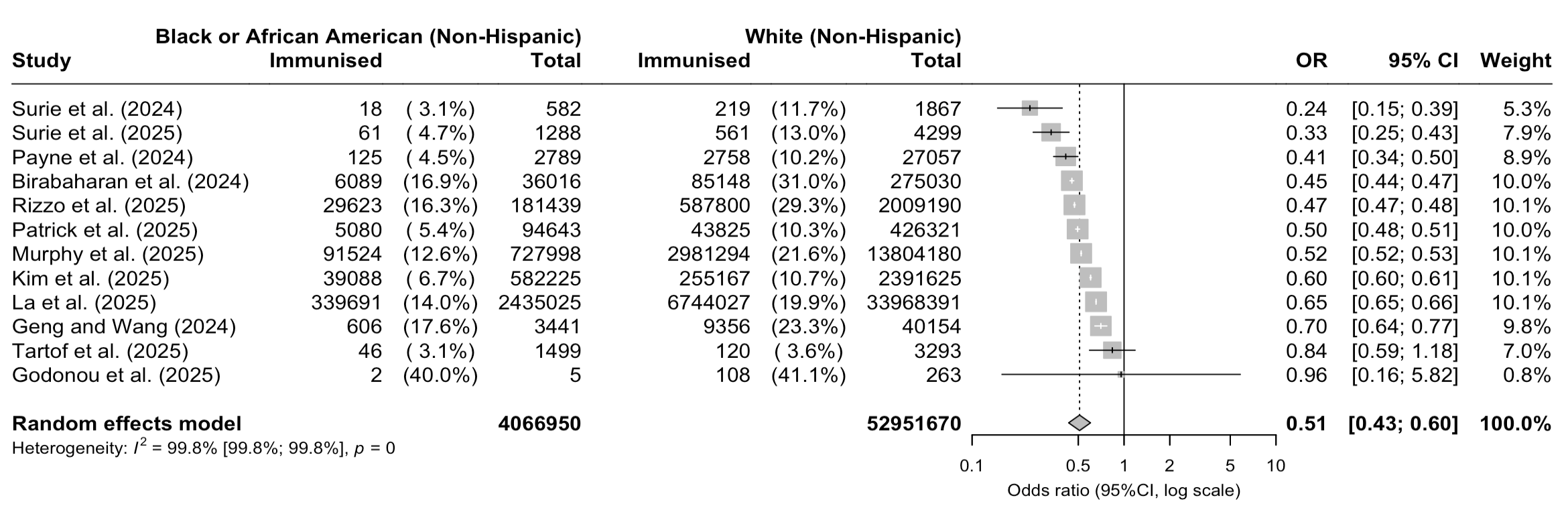


^a^ White (non-Hispanic) population (ref.) compared to Black or African American (non-Hispanic) population.

**Figure S24. Uptake of RSV vaccines among eligible older adults aged 60 years or older in the United States stratified by racial group^a^ (sensitivity analysis – ‘low risk-of-bias’ studies only)**


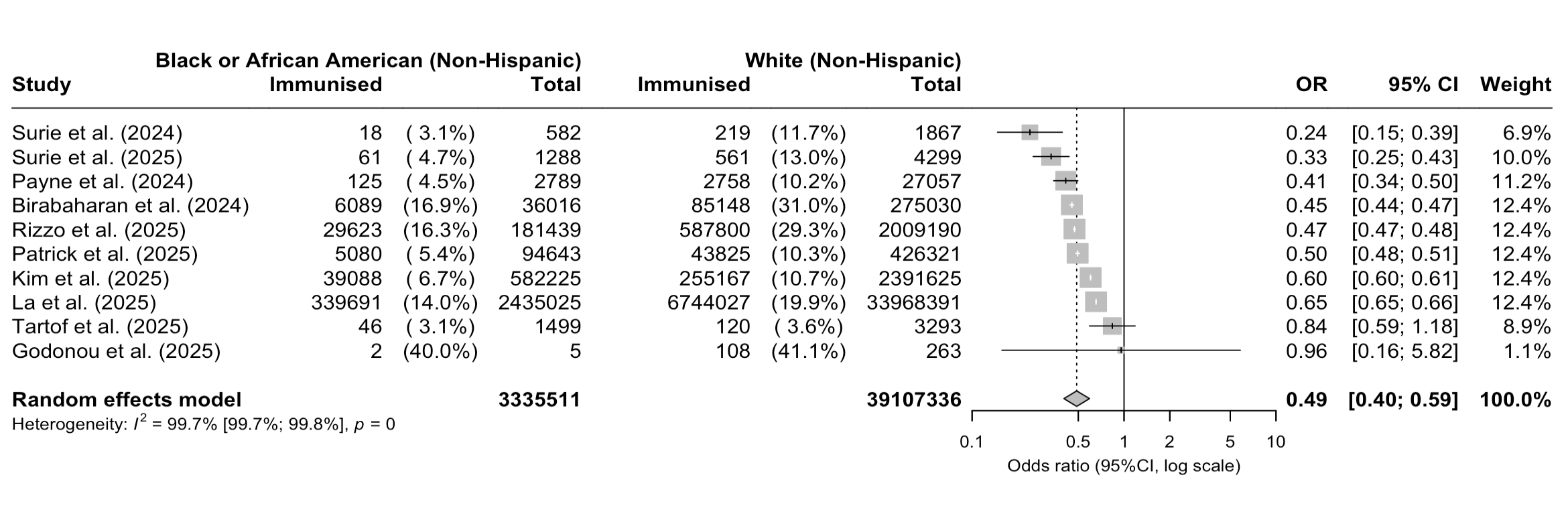


^a^ White (non-Hispanic) population (ref.) compared to Black or African American (non-Hispanic) population.

**Figure S25. Uptake of RSV vaccines among eligible older adults aged 60 years or older in the United States stratified by racial group^a^**


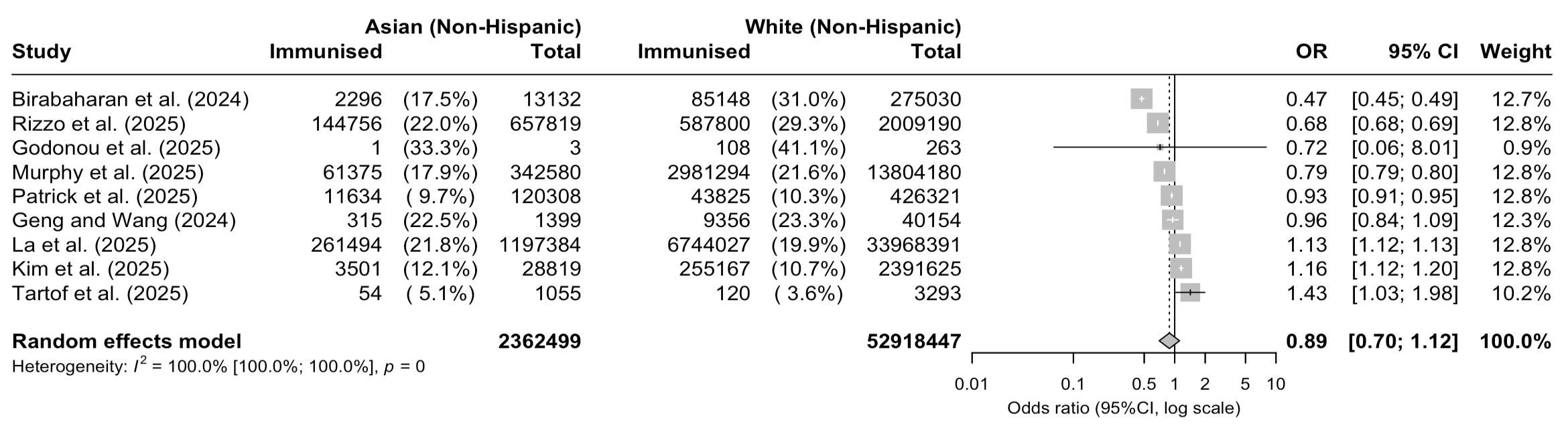


^a^ White (non-Hispanic) population (ref.) compared to Asian (non-Hispanic) population.

**Figure S26. Uptake of RSV vaccines among eligible older adults aged 60 years or older in the United States stratified by racial group^a^ (sensitivity analysis – ‘low risk-of-bias’ studies only)**


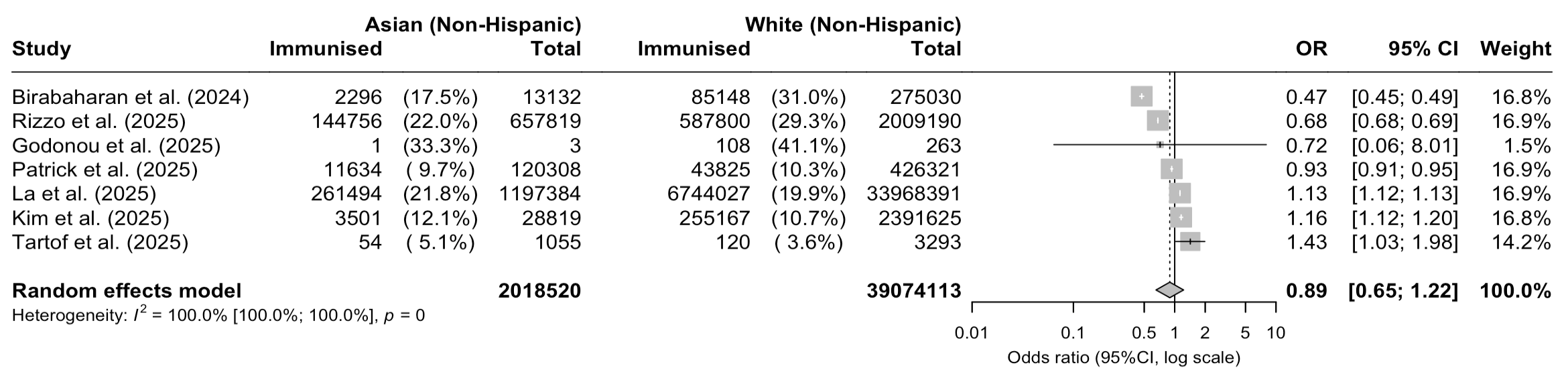


^a^ White (non-Hispanic) population (ref.) compared to Asian (non-Hispanic) population.

**Figure S27. Uptake of RSV vaccines among eligible older adults aged 60 years or older in the United States stratified by racial group^a^**


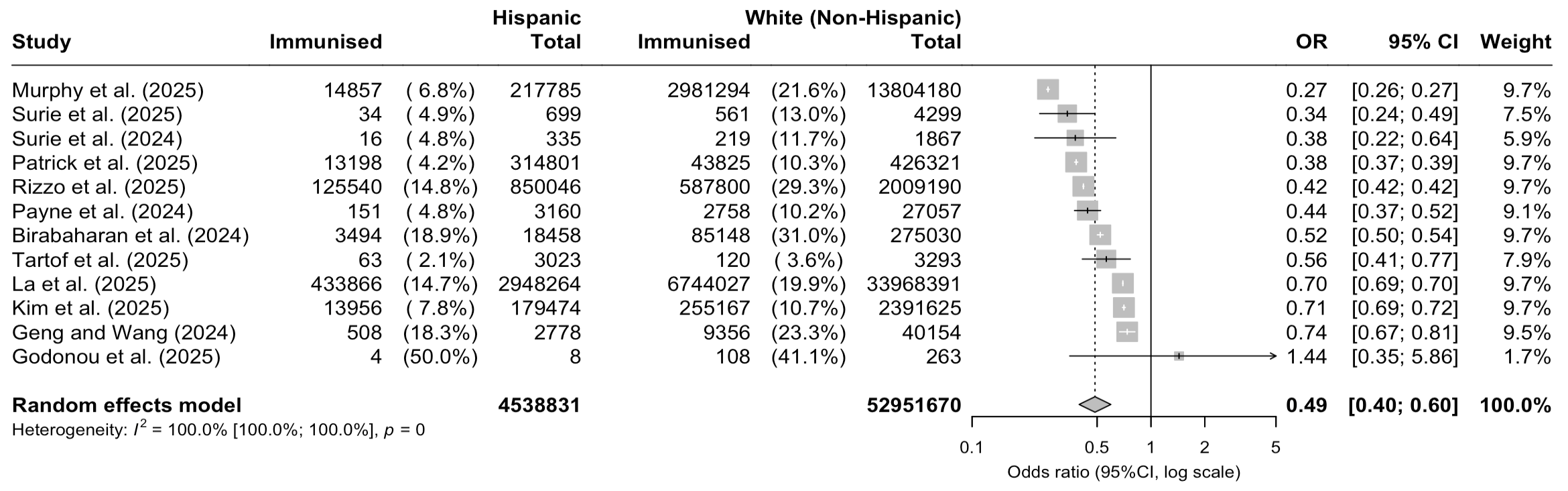


^a^ White (non-Hispanic) population (ref.) compared to Hispanic population.

**Figure S28. Uptake of RSV vaccines among eligible older adults aged 60 years or older in the United States stratified by racial group^a^ (sensitivity analysis – ‘low risk-of-bias’ studies only)**


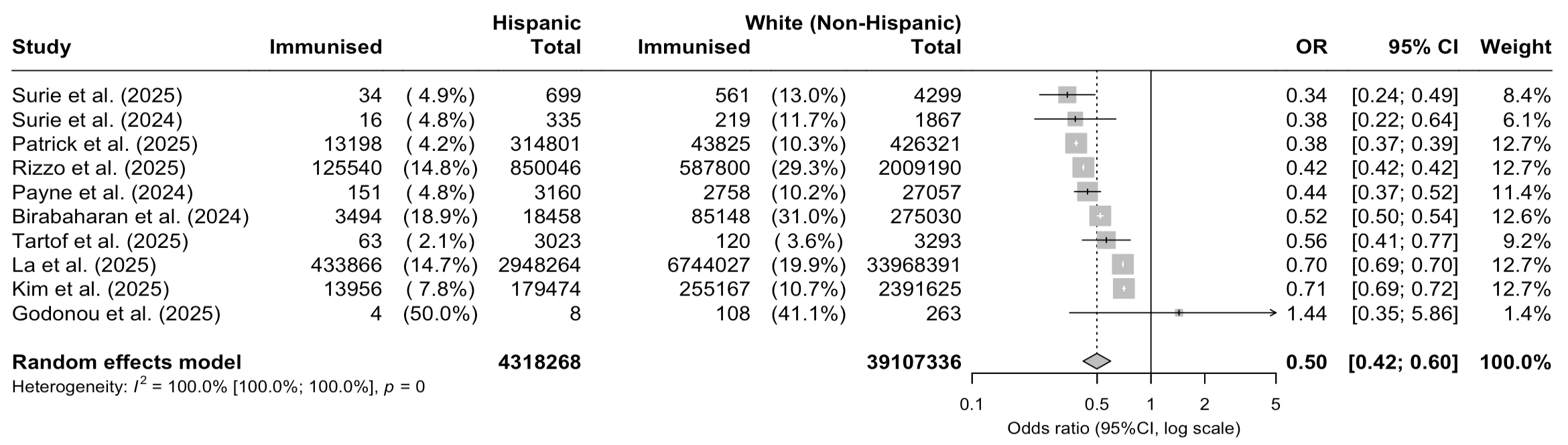


^a^ White (non-Hispanic) population (ref.) compared to Hispanic population.

**Figure S29. Uptake of RSV vaccines among eligible older adults aged 60 years or older in the United States stratified by racial group^a^**


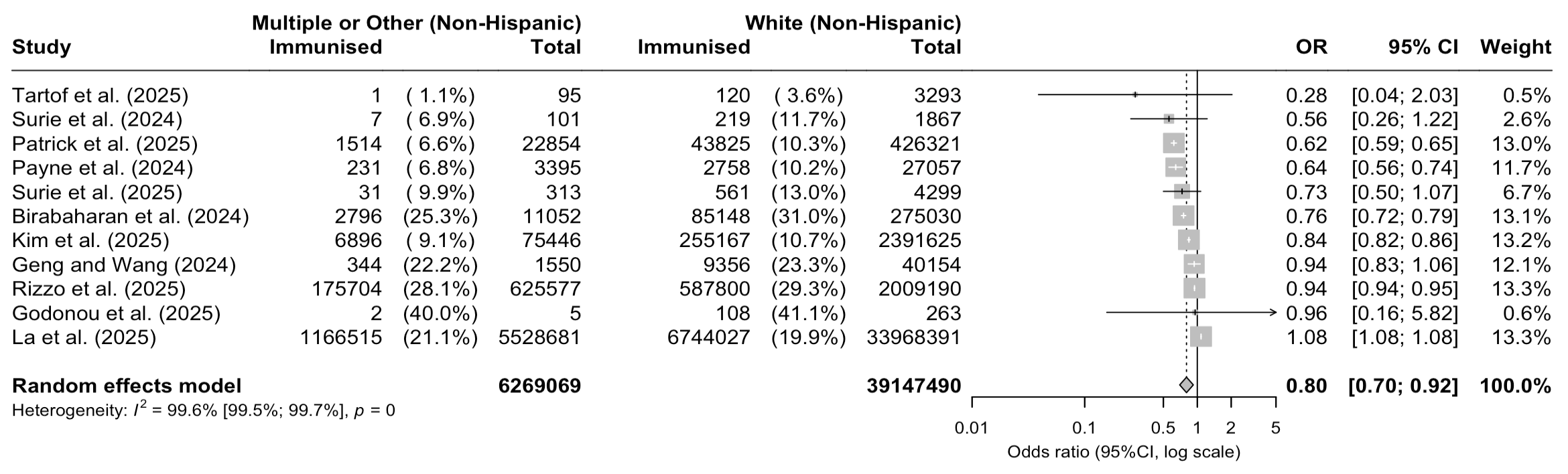


^a^ White (non-Hispanic) population (ref.) compared to Mixed or Other (non-Hispanic) population.

**Figure S30. Uptake of RSV vaccines among eligible older adults aged 60 years or older in the United States stratified by racial group^a^ (sensitivity analysis – ‘low risk-of-bias’ studies only)**


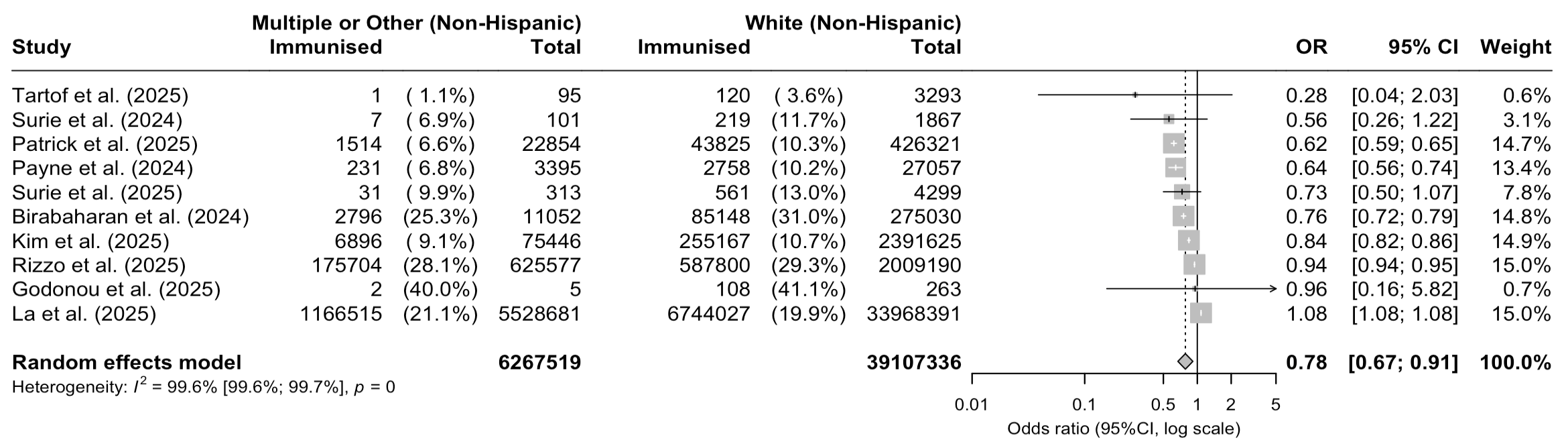


^a^ White (non-Hispanic) population (ref.) compared to Mixed or Other (non-Hispanic) population.

**Figure S31. Uptake of RSV vaccines among eligible older adults aged 60 years or older in the United States stratified by ethnic group^a^**


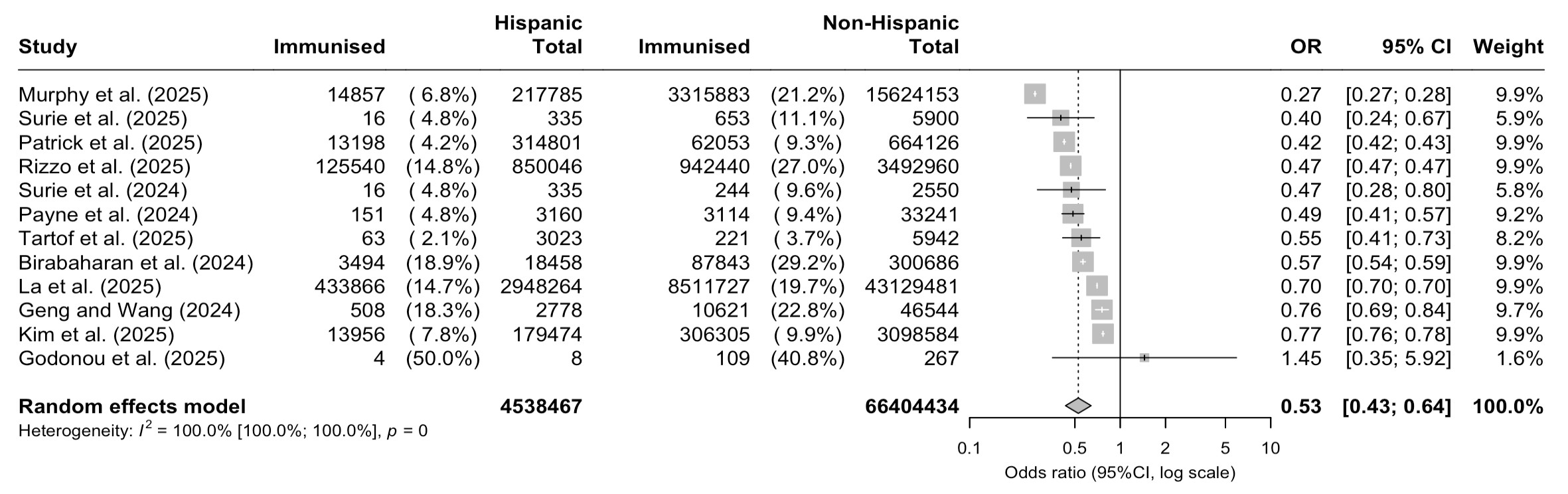


^a^ All non-Hispanic population (ref.) compared to Hispanic population.

**Figure S32. Uptake of RSV vaccines among eligible older adults aged 60 years or older in the United States stratified by ethnic group^a^ (sensitivity analysis – ‘low risk-of-bias’ studies only)**


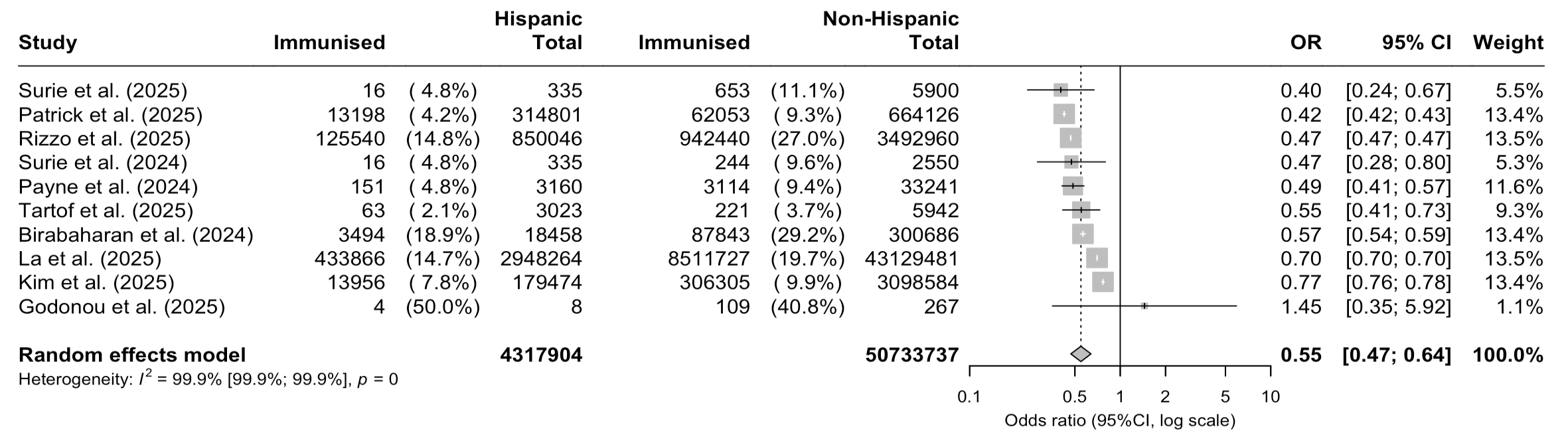


^a^ All non-Hispanic population (ref.) compared to Hispanic population.

# Supplement 7: Sensitivity analysis for RSV vaccine effectiveness in older adults

Sensitivity analyses were carried out using data from studies classified as ‘low risk of bias’ in the risk-of-bias quality assessment (Supplement 3). Sensitivity analyses were only done when at least three data points (studies) were available.

Figure S33. Sensitivity analysis of **RSV vaccine effectiveness (%) against RSV-associated hospital admissions in older adults (60 years or older).**


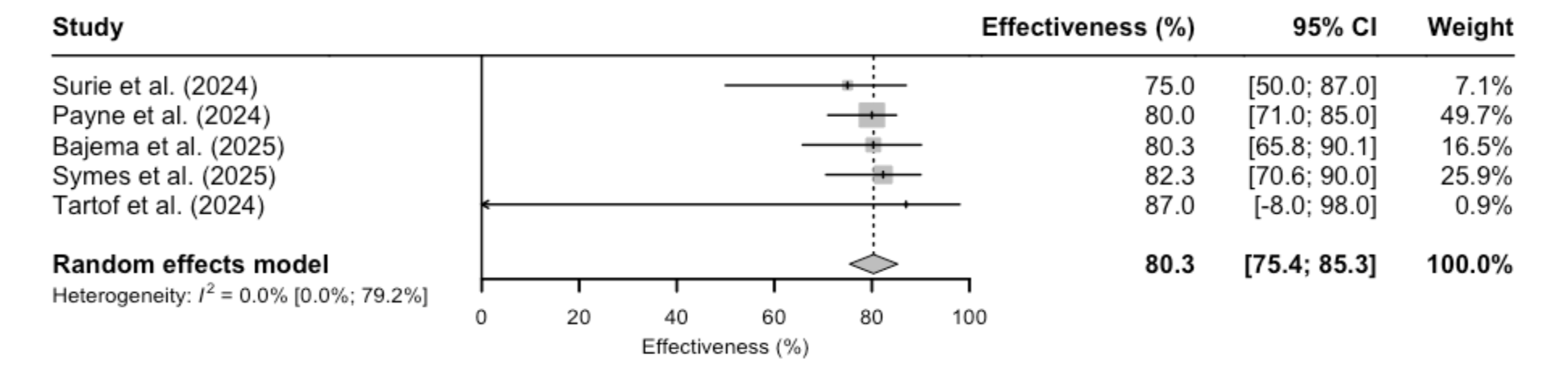


Figure S34. Sensitivity analysis of **RSV vaccine effectiveness (%) against RSV-associated urgent care and ED visits in older adults (60 years or older).**


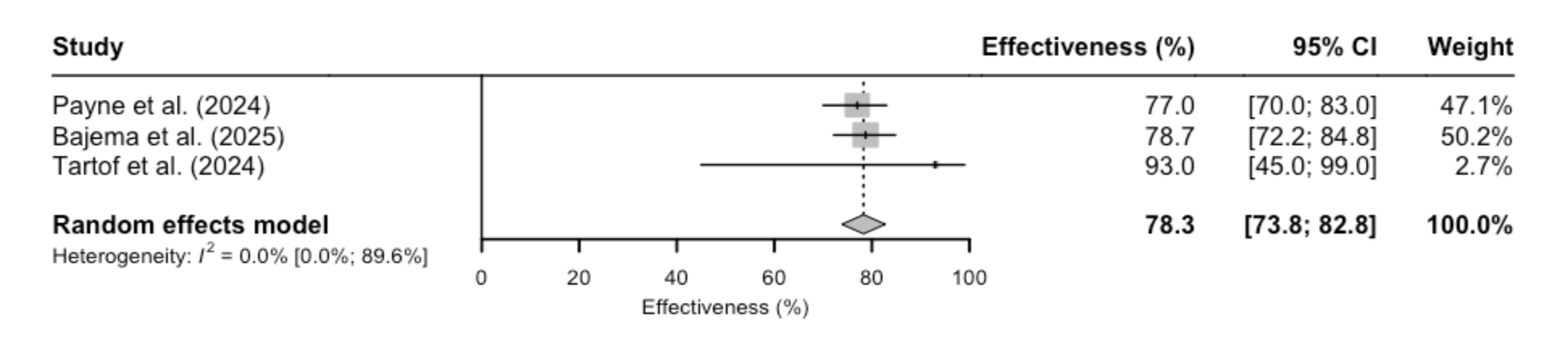


# Supplement 8: Meta-analysis of adverse event prevalence (%) in older adults aged 60 years and older after RSV vaccination

| **Adverse event** | **Cases** | **Total** | **No. of studies** | **Prevalence (95% CI)** | **I^2^** |
| --- | --- | --- | --- | --- | --- |
| **Any local reaction** | 6301 | 18631 | 3 | 42.0% (23.2-63.5) | 98.7% |
| Pain | 6494 | 24697 | 5 | 23.6% (10.5-44.9) | 99.6% |
| Swelling | 2248 | 24697 | 5 | 9.9% (6.7-14.3) | 94.1% |
| Erythema | 2217 | 24697 | 5 | 9.7% (7.4-12.5) | 93.0% |
| Pruritus | 1243 | 23778 | 4 | 5.3% (4.0-7.1) | 88.6% |
| Rash | 312 | 22684 | 4 | 1.4% (1.3-1.6) | 64.3% |
| **Any systemic reaction** | 5528 | 18631 | 3 | 31.7% (24.2-40.4) | 94.2% |
| Fatigue | 4365 | 24697 | 5 | 15.7% (10.5-22.9) | 99.0% |
| Headache | 3269 | 24697 | 5 | 11.9% (9.4-14.9) | 97.8% |
| Myalgia | 3367 | 23778 | 4 | 10.5% (5.7-18.3) | 99.4% |
| Arthralgia | 2103 | 24697 | 5 | 8.6% (7.0-10.5) | 92.2% |
| Fever | 1979 | 19550 | 4 | 8.0% (5.6-11.3) | 95.7% |
| Chills | 1908 | 23778 | 4 | 7.3% (5.1-10.3) | 97.5% |
| Nausea | 913 | 21765 | 3 | 4.2% (3.9-4.5) | 0.0% |
| Diarrhoea | 613 | 21765 | 3 | 2.8% (2.6-3.0) | 5.7% |

# Supplement 9: Overview of all reported safety signals

**Table 10.** The reported adverse events in the included studies.

| **Study** | **Population** | **Outcome definition** | **Vaccine product** | **Comparison group (if any)** | **Effect estimate** |
| --- | --- | --- | --- | --- | --- |
| Any adverse reaction | | | | | |
| Nguyen et. al. (2025) | Adults aged 60-69 years in Australia who received Arexvy RSVPreF protein vaccine within February 29, to September 27, 2024 | Any local, systemic, gastrointestinal or other adverse events within 3 days of vaccination. Local events were defined as local pain, itching, redness, swelling; systemic events were defined as fever, chills, headache, myalgia, arthralgia; gastrointestinal events include nausea, vomiting, diarrhoea, abdominal pain, other events include symptoms which were not included previously. | All RSVPreF vaccine doses (Arexvy, GSK) | NA | 741 (37%) of 2,013 participants with adverse reactions |
|  |  |  | Only RSVPreF vaccine dose |  | 622 (36%) of 1,739 participants with adverse reactions |
|  |  |  | RSVPreF vaccine dose received concomitantly |  | 119 (43%) of 274 participants with adverse reactions |
| Redjoul et al. (2025) | Adults >60 years more than 3 months post-allogeneic hematopoietic cell transplant | Any serious adverse reaction | RSVPreF (Abrysvo, Pfizer) | NA | No serious adverse events reported (out of total 92 participants) |
| Local adverse reactions | | | | | |
| Any local reaction | | | | | |
| Havlin et al. (2025) | Lung transplant recipients aged >60 years or older vaccinated between January 09 to March 11, 2024 | Injection site pain, swelling, erythema, or others within one-month post-vaccination | RSVPreF3 vaccine (Arexvy, GSK) | NA | 8 (26.7%) of 30 participants reported experiencing local reactions |
| Nguyen et. al. (2025) | Adults aged 60-69 years in Australia who received Arexvy RSVPreF protein vaccine within February 29, to September 27, 2024 | Any local adverse events within 3 days of vaccination. Local events were defined as local pain, itching, redness, and swelling | All RSVPreF vaccine doses (Arexvy, GSK) | NA | 605 (30%) of 2,013 participants with adverse reactions |
|  |  |  | Only RSVPreF vaccine dose |  | 504 (29%) of 1,739 participants with adverse reactions |
|  |  |  | RSVPreF vaccine dose received concomitantly |  | 101 (37%) of 274 participants with adverse reactions |
| Li et al. (2025) | Adults older than >60 years age who received RSV vaccine between May3, 2023 and December 27, 2024 | Defined as injection site erythema, swelling, warmth, pain, induration, pruritis, rash, reaction, local reaction, urticaria, vesicles, cellulitis, or odema | RSVPreF3+AS01 (Arexvy, GSK) or RSVpreF (Abrysvo, Pfizer) vaccine | NA | 1095 reports  EBGM: 2.77, EMGB05: 2.65 |
| Bruising | | | | | |
| Bao et al. (2025) | All eligible individuals (older adults and pregnant people) who received RSVPreF3 (between May 03, 2023, to March 28, 2025), RSVPreF (between May 31, 2023, to March 28, 2025), or mRNA-1345 (between May 31, 2024, to March 28, 2025) | Adverse event report of injection site bruising | RSVPreF3 | RSVPreF | **VAERS:** 129 reports  **Reporting odds ratio (ROR):** 7.67 (6.36-9.26) |
|  |  |  | RSVPreF | NA | **VAERS:** 19 reports |
|  |  |  | mRNA-1345 | NA | NA |
| Cellulitis | | | | | |
| Bao et al. (2025) | All eligible individuals (older adults and pregnant people) who received RSVPreF3 (between May 03, 2023, to March 28, 2025), RSVPreF (between May 31, 2023, to March 28, 2025), or mRNA-1345 (between May 31, 2024, to March 28, 2025) | Adverse event report of injection site cellulitis | RSVPreF3 | RSVPreF | **VAERS:** 12 reports  **Reporting odds ratio (ROR):** 2.03 (1.14-3.62) |
|  |  |  | RSVPreF | NA | NA |
|  |  |  | mRNA-1345 | NA | NA |
| Discharge | | | | | |
| Bao et al. (2025) | All eligible individuals (older adults and pregnant people) who received RSVPreF3 (between May 03, 2023, to March 28, 2025), RSVPreF (between May 31, 2023, to March 28, 2025), or mRNA-1345 (between May 31, 2024, to March 28, 2025) | Adverse event report of injection site discharge | RSVPreF3 | RSVPreF | **VAERS:** 6 reports  **Reporting odds ratio (ROR):** 3.21 (1.4-7.36) |
|  |  |  | RSVPreF | NA | **VAERS:** 5 reports |
|  |  |  | mRNA-1345 | NA | NA |
| Discolouration | | | | | |
| Bao et al. (2025) | All eligible individuals (older adults and pregnant people) who received RSVPreF3 (between May 03, 2023, to March 28, 2025), RSVPreF (between May 31, 2023, to March 28, 2025), or mRNA-1345 (between May 31, 2024, to March 28, 2025) | Adverse event report of injection site discolouration | RSVPreF3 | RSVPreF | **VAERS:** 33 reports  **Reporting odds ratio (ROR):** 8.18 (5.63-11.87) |
|  |  |  | RSVPreF | NA | **VAERS:** 10 reports |
|  |  |  | mRNA-1345 | NA | NA |
| Discomfort | | | | | |
| Bao et al. (2025) | All eligible individuals (older adults and pregnant people) who received RSVPreF3 (between May 03, 2023, to March 28, 2025), RSVPreF (between May 31, 2023, to March 28, 2025), or mRNA-1345 (between May 31, 2024, to March 28, 2025) | Adverse event report of injection site discomfort | RSVPreF3 | RSVPreF | **VAERS:** 7 reports  **Reporting odds ratio (ROR):** 2.44 (1.14-5.22) |
|  |  |  | RSVPreF | NA | NA |
|  |  |  | mRNA-1345 | NA | NA |
| Erythema | | | | | |
| Bao et al. (2025) | All eligible individuals (older adults and pregnant people) who received RSVPreF3 (between May 03, 2023, to March 28, 2025), RSVPreF (between May 31, 2023, to March 28, 2025), or mRNA-1345 (between May 31, 2024, to March 28, 2025) | Adverse event report of injection site erythema | RSVPreF3 | RSVPreF | **VAERS:** 553 reports  **Reporting odds ratio (ROR):** 4.62 (4.23-5.05) |
|  |  |  | RSVPreF | NA | **VAERS:** 146 reports |
|  |  |  | mRNA-1345 | NA | NA |
| Domnich et al. (2025) | Persons >60 years who received RSVPreF3 OA between February and  September 2024 | Local erythema within 7 days after vaccination | RSVPreF3 OA (Arexvy, GSK) | NA | **Mild:** 27 (56.3%) out of 48 adverse reaction reports  **Moderate:** 19 (39.6%) out of 48 adverse reaction reports  **Severe:** 2 (4.2%) out of 48 adverse reaction reports |
| Hause et al. (2024) | Persons >60 years who received an RSV vaccine starting October 20, 2023 | Redness at or near injection site within 7 days after vaccination | RSVPreF3+AS01 (Arexvy, GSK) or RSVpreF (Abrysvo, Pfizer) vaccine | NA | **V-safe surveillance system**: 1,344 (8.3%) out of 16,220 participants with adverse reactions  **VAERS:** 384 (13.2%) out of 2,919 reports |
|  |  |  | RSVPreF3+AS01 (Arexvy, GSK) |  | **V-safe surveillance system**: 671 (10.5%) out of 6,402 participants with adverse reactions  **VAERS:** 186 (9.2%) out of 2,026 reports |
|  |  |  | RSVpreF (Abrysvo, Pfizer) |  | **V-safe surveillance system**: 195 (5.0%) out of 3,882 participants with adverse reactions  **VAERS:** 57 (6.9%) out of 821 reports |
|  |  |  | Did not recall/do not know vaccine received |  | **V-safe surveillance system**: 478 (8.1%) out of 5,936 participants with adverse reactions  **VAERS:** 4 (5.6%) out of 72 reports |
| Levy et al. (2025) | Adult lung transplant recipients | Swelling or erythema within 7 days of vaccine receipt | RSVPreF3+AS01 (Arexvy, GSK) | NA | 4 (14.2) out of 28 participants experienced adverse event |
| Nguyen et. al. (2025) | Adults aged 60-69 years in Australia who received Arexvy RSVPreF protein vaccine within February 29, to September 27, 2024 | Local erythema within 3 days of vaccination | All RSVPreF vaccine doses (Arexvy, GSK) | NA | 204 (10%) of 2,013 participants |
|  |  |  | Only RSVPreF vaccine dose |  | 175 (10%) of 1,739 participants |
|  |  |  | RSVPreF vaccine dose received concomitantly |  | 29 (11%) of 274 participants |
| Hypoaesthesia | | | | | |
| Bao et al. (2025) | All eligible individuals (older adults and pregnant people) who received RSVPreF3 (between May 03, 2023, to March 28, 2025), RSVPreF (between May 31, 2023, to March 28, 2025), or mRNA-1345 (between May 31, 2024, to March 28, 2025) | Adverse event report of injection site hypoaesthesia | RSVPreF3 | RSVPreF | **VAERS:** 6 reports  **Reporting odds ratio (ROR):** 3.21 (1.4-7.36) |
|  |  |  | RSVPreF | NA | NA |
|  |  |  | mRNA-1345 | NA | NA |
| Induration | | | | | |
| Bao et al. (2025) | All eligible individuals (older adults and pregnant people) who received RSVPreF3 (between May 03, 2023, to March 28, 2025), RSVPreF (between May 31, 2023, to March 28, 2025), or mRNA-1345 (between May 31, 2024, to March 28, 2025) | Adverse event report of injection site induration | RSVPreF3 | RSVPreF | **VAERS:** 49 reports  **Reporting odds ratio (ROR):** 2.99 (2.24-3.99) |
|  |  |  | RSVPreF | NA | NA |
|  |  |  | mRNA-1345 | NA | NA |
| Inflammation | | | | | |
| Bao et al. (2025) | All eligible individuals (older adults and pregnant people) who received RSVPreF3 (between May 03, 2023, to March 28, 2025), RSVPreF (between May 31, 2023, to March 28, 2025), or mRNA-1345 (between May 31, 2024, to March 28, 2025) | Adverse event report of injection site inflammation | RSVPreF3 | RSVPreF | **VAERS:** 17 reports  **Reporting odds ratio (ROR):** 3 (1.83-4.9) |
|  |  |  | RSVPreF | NA | **VAERS:** 12 reports |
|  |  |  | mRNA-1345 | NA | NA |
| Irritation |  |  |  |  |  |
| Bao et al. (2025) | All eligible individuals (older adults and pregnant people) who received RSVPreF3 (between May 03, 2023, to March 28, 2025), RSVPreF (between May 31, 2023, to March 28, 2025), or mRNA-1345 (between May 31, 2024, to March 28, 2025) | Adverse event report of injection site irritation | RSVPreF3 | RSVPreF | **VAERS:** 10 reports  **Reporting odds ratio (ROR):** 7.51 (3.84-14.71) |
|  |  |  | RSVPreF | NA | NA |
|  |  |  | mRNA-1345 | NA | NA |
| Itching | | | | | |
| Hause et al. (2024) | Persons >60 years who received an RSV vaccine starting October 20, 2023 | Itching at or near injection site within 7 days after vaccination | RSVPreF3+AS01 (Arexvy, GSK) or RSVpreF (Abrysvo, Pfizer) vaccine | NA | **V-safe surveillance system**: 904 (5.6%) out of 16,220 participants with adverse reactions |
|  |  |  | RSVPreF3+AS01 (Arexvy, GSK) |  | **V-safe surveillance system**: 412 (6.4%) out of 6,402 participants with adverse reactions |
|  |  |  | RSVpreF (Abrysvo, Pfizer) |  | **V-safe surveillance system**: 162 (4.2%) out of 3,882 participants with adverse reactions |
|  |  |  | Did not recall/do not know vaccine received |  | **V-safe surveillance system**: 330 (5.6%) out of 5,936 participants with adverse reactions |
| Nguyen et. al. (2025) | Adults aged 60-69 years in Australia who received Arexvy RSVPreF protein vaccine within February 29, to September 27, 2024 | Local itching within 3 days of vaccination | All RSVPreF vaccine doses (Arexvy, GSK) | NA | 91 (4.5%) of 2,013 participants with adverse reactions |
|  |  |  | Only RSVPreF vaccine dose |  | 73 (4.2%) of 1,739 participants with adverse reactions |
|  |  |  | RSVPreF vaccine dose received concomitantly |  | 18 (6.6%) of 274 participants with adverse reactions |
| Mass | | | | | |
| Bao et al. (2025) | All eligible individuals (older adults and pregnant people) who received RSVPreF3 (between May 03, 2023, to March 28, 2025), RSVPreF (between May 31, 2023, to March 28, 2025), or mRNA-1345 (between May 31, 2024, to March 28, 2025) | Adverse event report of injection site mass | RSVPreF3 | RSVPreF | **VAERS:** 53 reports  **Reporting odds ratio (ROR):** 5.12 (3.85-6.81) |
|  |  |  | RSVPreF | NA | **VAERS:** 23 reports |
|  |  |  | mRNA-1345 | NA | NA |
| Mobility (reduced) | | | | | |
| Bao et al. (2025) | All eligible individuals (older adults and pregnant people) who received RSVPreF3 (between May 03, 2023, to March 28, 2025), RSVPreF (between May 31, 2023, to March 28, 2025), or mRNA-1345 (between May 31, 2024, to March 28, 2025) | Adverse event report of decreased mobility of injected limb | RSVPreF3 | RSVPreF | **VAERS:** 79 reports  **Reporting odds ratio (ROR):** 3.59 (2.85-4.52) |
|  |  |  | RSVPreF | NA | NA |
|  |  |  | mRNA-1345 | NA | NA |
| Nodule | | | | | |
| Bao et al. (2025) | All eligible individuals (older adults and pregnant people) who received RSVPreF3 (between May 03, 2023, to March 28, 2025), RSVPreF (between May 31, 2023, to March 28, 2025), or mRNA-1345 (between May 31, 2024, to March 28, 2025) | Adverse event report of injection site nodule | RSVPreF3 | RSVPreF | **VAERS:** 37 reports  **Reporting odds ratio (ROR):** 7.82 (5.5-11.1) |
|  |  |  | RSVPreF | NA | **VAERS:** 8 reports |
|  |  |  | mRNA-1345 | NA | NA |
| Pain | | | | | |
| Bao et al. (2025) | All eligible individuals (older adults and pregnant people) who received RSVPreF3 (between May 03, 2023, to March 28, 2025), RSVPreF (between May 31, 2023, to March 28, 2025), or mRNA-1345 (between May 31, 2024, to March 28, 2025) | Adverse event report of injection site pain | RSVPreF3 | RSVPreF | **VAERS:** 639 reports  **Reporting odds ratio (ROR):** 4.74 (4.36-5.15) |
|  |  |  | RSVPreF | NA | **VAERS:** 163 reports |
|  |  |  | mRNA-1345 | NA | NA |
| Domnich et al. (2025) | Persons >60 years who received RSVPreF3 OA between February and  September 2024 | Local pain within 7 days after vaccination | RSVPreF3 OA (Arexvy, GSK) | NA | **Mild:** 159 (75.7%) out of 210 adverse reaction reports  **Moderate:** 45 (21.4%) out of 210 adverse reaction reports  **Severe:** 6 (2.9%) out of 210 adverse reaction reports |
| Hall et al. (2025) | Adults >18 years who underwent allogenic haematopoietic cell transplant (alloHCT) or lung transplant (LT) and administered a single dose of RSVPreF3 | Grade 1 pain at injection site | RSVPreF3 (Arexvy, GSK) | NA | Allogeneic haematopoietic cell transplant patients: 31 (67.4%) out of 46 participants  Lung transplant patients: 23 (57.5%) out of 40 participants |
| Hause et al. (2024) | Persons >60 years who received an RSV vaccine starting October 20, 2023 | Pain at or near injection site within 7 days after vaccination | RSVPreF3+AS01 (Arexvy, GSK) or RSVpreF (Abrysvo, Pfizer) vaccine | NA | **V-safe surveillance system**: 5,026 (31%) out of 16,220 participants with adverse reactions  **VAERS:** 370 (12.7%) out of 2,919 reports  4 (1.4%) cases out of 281 serious reports |
|  |  |  | RSVPreF3+AS01 (Arexvy, GSK) |  | **V-safe surveillance system**: 2,641 (41.3%) out of 6,402 participants with adverse reactions  **VAERS:** 291 (14.4%) out of 2,026 reports  4 (2.4%) cases out of 167 serious reports |
|  |  |  | RSVpreF (Abrysvo, Pfizer) |  | **V-safe surveillance system**: 688 (17.7%) out of 3,882 participants with adverse reactions  **VAERS:** 72 (8.8%) out of 821 reports  0 cases out of 98 serious reports |
|  |  |  | Did not recall/do not know vaccine received |  | **V-safe surveillance system**: 1,697 (28.6%) out of 5,936 participants with adverse reactions  **VAERS:** 7 (9.7%) out of 72 reports  0 cases out of 16 serious reports |
| Levy et al. (2025) | Adult lung transplant recipients | Pain at injection site within 7 days of vaccine receipt | RSVPreF3+AS01 (Arexvy, GSK) | NA | 8 (28.6%) out of 28 participants experienced adverse event |
| Lotscher et al. (2025) | Adult patients more than 3 months post-allogeneic hematopoietic cell transplant | Report of injection site pain | RSVPreF3 OA | NA | 42 reports (out of 82 participants) |
| Nguyen et. al. (2025) | Adults aged 60-69 years in Australia who received Arexvy RSVPreF protein vaccine within February 29, to September 27, 2024 | Local pain within 3 days of vaccination | All RSVPreF vaccine doses (Arexvy, GSK) | NA | 518 (26%) of 2,013 participants with adverse reactions |
|  |  |  | Only RSVPreF vaccine dose |  | 429 (25%) of 1,739 participants with adverse reactions |
|  |  |  | RSVPreF vaccine dose received concomitantly |  | 89 (32%) of 274 participants with adverse reactions |
| Pruritus | | | | | |
| Bao et al. (2025) | All eligible individuals (older adults and pregnant people) who received RSVPreF3 (between May 03, 2023, to March 28, 2025), RSVPreF (between May 31, 2023, to March 28, 2025), or mRNA-1345 (between May 31, 2024, to March 28, 2025) | Adverse event report of injection site pruritus | RSVPreF3 | RSVPreF | **VAERS:** 214 reports  **Reporting odds ratio (ROR):** 5.65 (4.9-6.53) |
|  |  |  | RSVPreF | NA | **VAERS:** 57 reports |
|  |  |  | mRNA-1345 | NA | NA |
| Domnich et al. (2025) | Persons >60 years who received RSVPreF3 OA between February and  September 2024 | Pruritis within 7 days after vaccination | RSVPreF3 OA (Arexvy, GSK) | NA | **Mild:** 19 (76.0%) out of 25 adverse reaction reports  **Moderate:** 6 (24.0%) out of 25 adverse reaction reports  **Severe:** 0 out of 25 adverse reaction reports |
| Rash | | | | | |
| Bao et al. (2025) | All eligible individuals (older adults and pregnant people) who received RSVPreF3 (between May 03, 2023, to March 28, 2025), RSVPreF (between May 31, 2023, to March 28, 2025), or mRNA-1345 (between May 31, 2024, to March 28, 2025) | Adverse event report of injection site rash | RSVPreF3 | RSVPreF | **VAERS:** 77 reports  **Reporting odds ratio (ROR):** 3.63 (2.87-4.58) |
|  |  |  | RSVPreF | NA | **VAERS:** 24 reports |
|  |  |  | mRNA-1345 | NA | NA |
| Hause et al. (2024) | Persons >60 years who received an RSV vaccine starting October 20, 2023 | Rash at or near injection site within 7 days after vaccination | RSVPreF3+AS01 (Arexvy, GSK) or RSVpreF (Abrysvo, Pfizer) vaccine | NA | **V-safe surveillance system**: 225 (1.4%) out of 16,220 participants with adverse reactions |
|  |  |  | RSVPreF3+AS01 (Arexvy, GSK) |  | **V-safe surveillance system**: 101 (1.6%) out of 6,402 participants with adverse reactions |
|  |  |  | RSVpreF (Abrysvo, Pfizer) |  | **V-safe surveillance system**: 38 (1.0%) out of 3,882 participants with adverse reactions |
|  |  |  | Did not recall/do not know vaccine received |  | **V-safe surveillance system**: 86 (1.4%) out of 5,936 participants with adverse reactions |
| Scab | | | | | |
| Bao et al. (2025) | All eligible individuals (older adults and pregnant people) who received RSVPreF3 (between May 03, 2023, to March 28, 2025), RSVPreF (between May 31, 2023, to March 28, 2025), or mRNA-1345 (between May 31, 2024, to March 28, 2025) | Adverse event report of injection site scab | RSVPreF3 | RSVPreF | **VAERS:** 16 reports  **Reporting odds ratio (ROR):** 20.77 (11.43-37.74) |
|  |  |  | RSVPreF | NA | NA |
|  |  |  | mRNA-1345 | NA | NA |
| Swelling | | | | | |
| Bao et al. (2025) | All eligible individuals (older adults and pregnant people) who received RSVPreF3 (between May 03, 2023, to March 28, 2025), RSVPreF (between May 31, 2023, to March 28, 2025), or mRNA-1345 (between May 31, 2024, to March 28, 2025) | Adverse event report of injection site swelling | RSVPreF3 | RSVPreF | **VAERS:** 448 reports  **Reporting odds ratio (ROR):** 4.63 (4.19 -5.11) |
|  |  |  | RSVPreF | NA | **VAERS:** 114 reports |
|  |  |  | mRNA-1345 | NA | NA |
| Domnich et al. (2025) | Persons >60 years who received RSVPreF3 OA between February and  September 2024 | Local swelling within 7 days after vaccination. Defined as s <2 cm, 2–5 cm and >5 cm for grades mild, moderate and severe respectively | RSVPreF3 OA (Arexvy, GSK) | NA | **Mild:** 52 (80.0%) out of 65 adverse reaction reports  **Moderate:** 12 (18.5%) out of 65 adverse reaction reports  **Severe:** 1 (1.5%) out of 65 adverse reaction reports |
| Hause et al. (2024) | Persons >60 years who received an RSV vaccine starting October 20, 2023 | Swelling at injection site within 7 days after vaccination | RSVPreF3+AS01 (Arexvy, GSK) or RSVpreF (Abrysvo, Pfizer) vaccine | NA | **V-safe surveillance system**: 1,451 (8.9%) out of 16,220 participants with adverse reactions  **VAERS:** 376 (12.9%) out of 2,919 reports |
|  |  |  | RSVPreF3+AS01 (Arexvy, GSK) |  | **V-safe surveillance system**: 737 (11.5%) out of 6,402 participants with adverse reactions  **VAERS:** 187 (9.2%) out of 2,026 reports |
|  |  |  | RSVpreF (Abrysvo, Pfizer) |  | **V-safe surveillance system**: 217 (5.6%) out of 3,882 participants with adverse reactions  **VAERS:** 51 (6.2%) out of 821 reports |
|  |  |  | Did not recall/do not know vaccine received |  | **V-safe surveillance system**: 497 (8.4%) out of 5,936 participants with adverse reactions  **VAERS:** 2 (2.8%) out of 72 reports |
| Levy et al. (2025) | Adult lung transplant recipients | Swelling or erythema within 7 days of vaccine receipt | RSVPreF3+AS01 (Arexvy, GSK) | NA | 4 (14.2) out of 28 participants experienced adverse event |
| Nguyen et. al. (2025) | Adults aged 60-69 years in Australia who received Arexvy RSVPreF protein vaccine within February 29, to September 27, 2024 | Local swelling within 3 days of vaccination | All RSVPreF vaccine doses (Arexvy, GSK) | NA | 223 (11%) of 2,013 participants |
|  |  |  | Only RSVPreF vaccine dose |  | 180 (10%) of 1,739 participants |
|  |  |  | RSVPreF vaccine dose received concomitantly |  | 43 (16%) of 274 participants |
| Ulcer | | | | | |
| Bao et al. (2025) | All eligible individuals (older adults and pregnant people) who received RSVPreF3 (between May 03, 2023, to March 28, 2025), RSVPreF (between May 31, 2023, to March 28, 2025), or mRNA-1345 (between May 31, 2024, to March 28, 2025) | Adverse event report of injection site ulcer | RSVPreF3 | RSVPreF | **VAERS:** 6 reports  **Reporting odds ratio (ROR):** 21.41 (8.03-57.04) |
|  |  |  | RSVPreF | NA | NA |
|  |  |  | mRNA-1345 | NA | NA |
| Underarm swelling/tenderness | | | | | |
| Hause et al. (2024) | Persons >60 years who received an RSV vaccine starting October 20, 2023 | Swelling or tenderness near or in underarm region within 7 days after vaccination | RSVPreF3+AS01 (Arexvy, GSK) or RSVpreF (Abrysvo, Pfizer) vaccine | NA | **V-safe surveillance system**: 318 (2.0%) out of 16,220 participants with adverse reactions |
|  |  |  | RSVPreF3+AS01 (Arexvy, GSK) |  | **V-safe surveillance system**: 165 (2.6%) out of 6,402 participants with adverse reactions |
|  |  |  | RSVpreF (Abrysvo, Pfizer) |  | **V-safe surveillance system**: 69 (1.8%) out of 3,882 participants with adverse reactions |
|  |  |  | Did not recall/do not know vaccine received |  | **V-safe surveillance system**: 84 (1.4%) out of 5,936 participants with adverse reactions |
| Warmth | | | | | |
| Bao et al. (2025) | All eligible individuals (older adults and pregnant people) who received RSVPreF3 (between May 03, 2023, to March 28, 2025), RSVPreF (between May 31, 2023, to March 28, 2025), or mRNA-1345 (between May 31, 2024, to March 28, 2025) | Adverse event report of injection site warmth | RSVPreF3 | RSVPreF | **VAERS:** 188 reports  **Reporting odds ratio (ROR):** 4.08 (3.51-4.74) |
|  |  |  | RSVPreF | NA | **VAERS:** 66 reports |
|  |  |  | mRNA-1345 | NA | NA |
| Systemic adverse reactions | | | | | |
| Any systemic reaction | | | | | |
| Havlin et al. (2025) | Lung transplant recipients aged >60 years or older vaccinated between January 09 to March 11, 2024 | Myalgia, fatigue, fever <38 ^o^C, fever > 38 ^o^C, headache, arthralgia, nausea/vomiting, shortness of breath within one-month post-vaccination | RSVPreF3 vaccine (Arexvy, GSK) | NA | 5 (16.7%) of 30 participants reported experiencing local reactions |
| Nguyen et. al. (2025) | Adults aged 60-69 years in Australia who received Arexvy RSVPreF protein vaccine within February 29, to September 27, 2024 | Any systemic adverse event within 3 days of vaccination. Systemic events were defined as fever, chills, headache, myalgia, arthralgia | All RSVPreF vaccine doses (Arexvy, GSK) | NA | 527 (26%) of 2,013 participants reported a systemic adverse event |
|  |  |  | Only RSVPreF vaccine dose |  | 436 (25%) of 1,739 participants reported a systemic adverse event |
|  |  |  | RSVPreF vaccine dose received concomitantly |  | 91 (33%) of 274 participants reported a systemic adverse event |
| Abnormal limb (detected on x-ray) | | | | | |
| Bao et al. (2025) | All eligible individuals (older adults and pregnant people) who received RSVPreF3 (between May 03, 2023, to March 28, 2025), RSVPreF (between May 31, 2023, to March 28, 2025), or mRNA-1345 (between May 31, 2024, to March 28, 2025) | Adverse event report of abnormal limb detected on x-ray | RSVPreF3 | RSVPreF | **VAERS:** 5 reports  **Reporting odds ratio (ROR):** 2.85 (1.15-7.06) |
|  |  |  | RSVPreF | NA | **VAERS:** 6 reports |
|  |  |  | mRNA-1345 | NA | NA |
| Ascending flaccid paralysis | | | | | |
| Bao et al. (2025) | All eligible individuals (older adults and pregnant people) who received RSVPreF3 (between May 03, 2023, to March 28, 2025), RSVPreF (between May 31, 2023, to March 28, 2025), or mRNA-1345 (between May 31, 2024, to March 28, 2025) | Adverse event report of ascending flaccid paralysis | RSVPreF3 | RSVPreF | **VAERS:** 5 reports  **Reporting odds ratio (ROR):** 35.68 (10.89-116.91) |
|  |  |  | RSVPreF | NA | NA |
|  |  |  | mRNA-1345 | NA | NA |
| Li et al. (2025) | Adults older than >60 years age who received RSV vaccine between May3, 2023 and December 27, 2024 | Report of tenderness | RSVPreF3+AS01 (Arexvy, GSK) or RSVpreF (Abrysvo, Pfizer) vaccine | NA | 7 reports  EBGM: 8.42, EMGB05: 2.26 |
| Allergic reaction | | | | | |
| Hause et al. (2024) | Persons >60 years who received an RSV vaccine starting October 20, 2023 | Allergic reaction (including angioedema) within 7 days after vaccination | RSVPreF3+AS01 (Arexvy, GSK) or RSVpreF (Abrysvo, Pfizer) vaccine | NA | **VAERS:** 7 cases out of 281 serious reports |
|  |  |  | RSVPreF3+AS01 (Arexvy, GSK) |  | **VAERS:** 3 cases out of 167 serious reports |
|  |  |  | RSVpreF (Abrysvo, Pfizer) |  | **VAERS:** 4 cases out of 98 serious reports |
|  |  |  | Did not recall/do not know vaccine received |  | **VAERS:** 0 cases out of 16 serious reports |
| Anaphylaxis | | | | | |
| Hause et al. (2024) | Persons >60 years who received an RSV vaccine starting October 20, 2023 | Anaphylaxis within 7 days after vaccination | RSVPreF3+AS01 (Arexvy, GSK) or RSVpreF (Abrysvo, Pfizer) vaccine | NA | **VAERS:** 2 cases out of 281 serious reports |
|  |  |  | RSVPreF3+AS01 (Arexvy, GSK) |  | **VAERS:** 1 case out of 167 serious reports |
|  |  |  | RSVpreF (Abrysvo, Pfizer) |  | **VAERS:** 1 case out of 98 serious reports |
|  |  |  | Did not recall/do not know vaccine received |  | **VAERS:** 0 cases out of 16 serious reports |
| Arrhythmia | | | | | |
| Bao et al. (2025) | All eligible individuals (older adults and pregnant people) who received RSVPreF3 (between May 03, 2023, to March 28, 2025), RSVPreF (between May 31, 2023, to March 28, 2025), or mRNA-1345 (between May 31, 2024, to March 28, 2025) | Adverse event report of arrhythmia | RSVPreF3 | RSVPreF | **VAERS:** 13 reports  **Reporting odds ratio (ROR):** 1.8 (1.03-3.13) |
|  |  |  | RSVPreF | NA | **VAERS:** 13 reports |
|  |  |  | mRNA-1345 | NA | **VAERS:** 5 reports |
| Hause et al. (2024) | Persons >60 years who received an RSV vaccine starting October 20, 2023 | Arrythmia within 7 days after vaccination | RSVPreF3+AS01 (Arexvy, GSK) or RSVpreF (Abrysvo, Pfizer) vaccine | NA | **VAERS:** 6 (2.1%) cases out of 281 serious reports |
|  |  |  | RSVPreF3+AS01 (Arexvy, GSK) |  | **VAERS:** 4 (2.4%) cases out of 167 serious reports |
|  |  |  | RSVpreF (Abrysvo, Pfizer) |  | **VAERS:** 1 (1.0%) case out of 98 serious reports |
|  |  |  | Did not recall/do not know vaccine received |  | **VAERS:** 1 (6.3%) case out of 16 serious reports |
| Arthralgia | | | | | |
| Bao et al. (2025) | All eligible individuals (older adults and pregnant people) who received RSVPreF3 (between May 03, 2023, to March 28, 2025), RSVPreF (between May 31, 2023, to March 28, 2025), or mRNA-1345 (between May 31, 2024, to March 28, 2025) | Adverse event report of arthralgia | RSVPreF3 | RSVPreF | **VAERS:** 319 reports  **Reporting odds ratio (ROR):** 2.02 (1.81-2.27) |
|  |  |  | RSVPreF | NA | **VAERS:** 162 reports |
|  |  |  | mRNA-1345 | NA | NA |
| Domnich et al. (2025) | Persons >60 years who received RSVPreF3 OA between February and  September 2024 | Arthralgia within 7 days after vaccination | RSVPreF3 OA (Arexvy, GSK) | NA | **Mild:** 15 (55.6%) out of 27 adverse reaction reports  **Moderate:** 9 (33.3%) out of 27 adverse reaction reports  **Severe:** 3 (11.1%) out of 27 adverse reaction reports |
| Hause et al. (2024) | Persons >60 years who received an RSV vaccine starting October 20, 2023 | Joint pain within 7 days after vaccination | RSVPreF3+AS01 (Arexvy, GSK) or RSVpreF (Abrysvo, Pfizer) vaccine | NA | **V-safe surveillance system**: 1,488 (9.2%) out of 16,220 participants with adverse reactions  **VAERS:** 240 (8.2%) out of 2,919 reports |
|  |  |  | RSVPreF3+AS01 (Arexvy, GSK) |  | **V-safe surveillance system**: 756 (11.8%) out of 6,402 participants with adverse reactions  **VAERS:** 183 (9.0%) out of 2,026 reports |
|  |  |  | RSVpreF (Abrysvo, Pfizer) |  | **V-safe surveillance system**: 255 (6.6%) out of 3,882 participants with adverse reactions  **VAERS:** 85 (10.4%) out of 821 reports |
|  |  |  | Did not recall/do not know vaccine received |  | **V-safe surveillance system**: 477 (8.0%) out of 5,936 participants with adverse reactions  **VAERS:** 7 (9.7%) out of 72 reports |
| Nguyen et. al. (2025) | Adults aged 60-69 years in Australia who received Arexvy RSVPreF protein vaccine within February 29, to September 27, 2024 | Myalgia within 3 days of vaccination | All RSVPreF vaccine doses (Arexvy, GSK) | NA | 163 (8.1%) of 2,013 participants |
|  |  |  | Only RSVPreF vaccine dose |  | 139 (8.0%) of 1,739 participants |
|  |  |  | RSVPreF vaccine dose received concomitantly |  | 24 (8.8%) of 274 participants |
| Asthenia | | | | | |
| Bao et al. (2025) | All eligible individuals (older adults and pregnant people) who received RSVPreF3 (between May 03, 2023, to March 28, 2025), RSVPreF (between May 31, 2023, to March 28, 2025), or mRNA-1345 (between May 31, 2024, to March 28, 2025) | Adverse event report of asthenia | RSVPreF3 | RSVPreF | **VAERS:** 170 reports  **Reporting odds ratio (ROR):** 1.32 (1.13-1.54) |
|  |  |  | RSVPreF | NA | **VAERS:** 108 reports |
|  |  |  | mRNA-1345 | NA | NA |
| Atrial fibrillation | | | | | |
| Bao et al. (2025) | All eligible individuals (older adults and pregnant people) who received RSVPreF3 (between May 03, 2023, to March 28, 2025), RSVPreF (between May 31, 2023, to March 28, 2025), or mRNA-1345 (between May 31, 2024, to March 28, 2025) | Adverse event report of atrial fibrillation | RSVPreF3 | RSVPreF | **VAERS:** 57 reports  **Reporting odds ratio (ROR):** 1.77 (1.36-2.31) |
|  |  |  | RSVPreF | NA | **VAERS:** 28 reports |
|  |  |  | mRNA-1345 | NA | NA |
| Birabaharan et al. (2024) | Persons >60 years who received RSV vaccine between May 3, 2023, and May 3, 2024 | New-onset atrial fibrillation: occurence of atrial fibrillation in patients with no prior history within 42 days of vaccination | RSVPreF3+AS01 (Arexvy, GSK) or RSVpreF (Abrysvo, Pfizer) vaccine | Influenza vaccine | RR 1.06 (95% CI: 0.90-1.25) |
|  |  |  |  | Influenza or tetanus, diphtheria, and pertussis (Tdap) vaccine | RR 0.82 (95% CI: 0.66-1.02) |
|  |  | Recurrent atrial fibrillation: occurence of atrial fibrillation in patients with documented history of atrial fibrillation, within 42 days of vaccination |  | Influenza vaccine | RR 0.94 (95% CI: 0.91-0.97) |
|  |  |  |  | Influenza or tetanus, diphtheria, and pertussis (Tdap) vaccine | RR 0.89 (95% CI: 0.85-0.93) |
| Donahue et al. (2025) | Adults >60 years vaccinated with Abrysvo and Arexvy between (August 01, 2023 to September 28, 2024) | Report of atrial fibrillation within 1-21 days (risk interval) of vaccination | Arexvy (GSK) with same day administration of non-RSV vaccine | Day 43-63 (comparison interval) | 96 reports  RR (95% CI): 0.98 (0.72-1.34) |
|  |  |  | Arexvy (GSK) without same day administration of non-RSV vaccine |  | 222 reports  RR (95% CI): 0.90 (0.73-1.09) |
|  |  |  | Abrysvo (Pfizer) with same day administration of non-RSV vaccine |  | 9 reports  RR (95% CI): 0.57 (0.22-1.36) |
|  |  |  | Abrysvo (Pfizer) without same day administration of non-RSV vaccine |  | 26 reports  RR (95% CI): 0.63 (0.35-1.12) |
| Hause et al. (2024) | Persons >60 years who received an RSV vaccine starting October 20, 2023 | Atrial fibrillation within 7 days after vaccination | RSVPreF3+AS01 (Arexvy, GSK) or RSVpreF (Abrysvo, Pfizer) vaccine | NA | **VAERS:** 14 (5.0%) cases out of 281 serious reports |
|  |  |  | RSVPreF3+AS01 (Arexvy, GSK) |  | **VAERS:** 8 (4.8%) cases out of 167 serious reports |
|  |  |  | RSVpreF (Abrysvo, Pfizer) |  | **VAERS:** 3 (3.1%) cases out of 98 serious reports |
|  |  |  | Did not recall/do not know vaccine received |  | **VAERS:** 3 (18.8%) cases out of 16 serious reports |
| Bell’s palsy | | | | | |
| Donahue et al. (2025) | Adults >60 years vaccinated with Abrysvo and Arexvy between (August 01, 2023 to September 28, 2024) | Report of Bell’s palsy within 1-21 days (risk interval) of vaccination | Arexvy (GSK) with same day administration of non-RSV vaccine | Day 43-63 (comparison interval) | 5 reports  RR (95% CI): 0.57 (0.17-1.65) |
|  |  |  | Arexvy (GSK) without same day administration of non-RSV vaccine |  | 19 reports  RR (95% CI): 1.03 (0.47-2.27) |
|  |  |  | Abrysvo (Pfizer) with same day administration of non-RSV vaccine |  | 1 report  RR (95% CI): 0.69 (0.02-9.33) |
|  |  |  | Abrysvo (Pfizer) without same day administration of non-RSV vaccine |  | 4 reports  RR (95% CI): 0.77 (0.10-7.81) |
| Cellulitis | | | | | |
| Bao et al. (2025) | All eligible individuals (older adults and pregnant people) who received RSVPreF3 (between May 03, 2023, to March 28, 2025), RSVPreF (between May 31, 2023, to March 28, 2025), or mRNA-1345 (between May 31, 2024, to March 28, 2025) | Adverse event report of cellulitis | RSVPreF3 | RSVPreF | **VAERS:** 33 reports  **Reporting odds ratio (ROR):** 2.27 (1.6-3.23) |
|  |  |  | RSVPreF | NA | NA |
|  |  |  | mRNA-1345 | NA | NA |
| Cerebrospinal fluid (CSF) protein (increased) | | | | | |
| Bao et al. (2025) | All eligible individuals (older adults and pregnant people) who received RSVPreF3 (between May 03, 2023, to March 28, 2025), RSVPreF (between May 31, 2023, to March 28, 2025), or mRNA-1345 (between May 31, 2024, to March 28, 2025) | Adverse event report of increased CSF protein | RSVPreF3 | RSVPreF | **VAERS:** 7 reports  **Reporting odds ratio (ROR):** 3 (1.39-6.45) |
|  |  |  | RSVPreF | NA | **VAERS:** 10 reports |
|  |  |  | mRNA-1345 | NA | NA |
| Chills | | | | | |
| Bao et al. (2025) | All eligible individuals (older adults and pregnant people) who received RSVPreF3 (between May 03, 2023, to March 28, 2025), RSVPreF (between May 31, 2023, to March 28, 2025), or mRNA-1345 (between May 31, 2024, to March 28, 2025) | Adverse event report of chills | RSVPreF3 | RSVPreF | **VAERS:** 226 reports  **Reporting odds ratio (ROR):** 1.75 (1.53-2.01) |
|  |  |  | RSVPreF | NA | **VAERS:** 111 reports |
|  |  |  | mRNA-1345 | NA | NA |
| Domnich et al. (2025) | Persons >60 years who received RSVPreF3 OA between February and  September 2024 | Chills within 7 days after vaccination. Defined as <38 ◦C, 38–39 ◦C and >39 ◦C for grades mild, moderate and severe respectively | RSVPreF3 OA (Arexvy, GSK) | NA | **Mild:** 10 (62.5%) out of 16 adverse reaction reports  **Moderate:** 6 (37.5%) out of 16 adverse reaction reports  **Severe:** 0 out of 16 adverse reaction reports |
| Hause et al. (2024) | Persons >60 years who received an RSV vaccine starting October 20, 2023 | Chills within 7 days after vaccination | RSVPreF3+AS01 (Arexvy, GSK) or RSVpreF (Abrysvo, Pfizer) vaccine | NA | **V-safe surveillance system**: 1,491 (9.2%) out of 16,220 participants with adverse reactions |
|  |  |  | RSVPreF3+AS01 (Arexvy, GSK) |  | **V-safe surveillance system**: 772 (12.1%) out of 6,402 participants with adverse reactions |
|  |  |  | RSVpreF (Abrysvo, Pfizer) |  | **V-safe surveillance system**: 226 (5.8%) out of 3,882 participants with adverse reactions |
|  |  |  | Did not recall/do not know vaccine received |  | **V-safe surveillance system**: 493 (8.3%) out of 5,936 participants with adverse reactions |
| Nguyen et. al. (2025) | Adults aged 60-69 years in Australia who received Arexvy RSVPreF protein vaccine within February 29, to September 27, 2024 | Chills within 3 days of vaccination | All RSVPreF vaccine doses (Arexvy, GSK) | NA | 154 (7.7%) of 2,013 participants |
|  |  |  | Only RSVPreF vaccine dose |  | 131 (7.5%) of 1,739 participants |
|  |  |  | RSVPreF vaccine dose received concomitantly |  | 23 (8.4%) of 274 participants |
| Congestive heart failure | | | | | |
| Hause et al. (2024) | Persons >60 years who received an RSV vaccine starting October 20, 2023 | Congestive heart failure within 7 days after vaccination | RSVPreF3+AS01 (Arexvy, GSK) or RSVpreF (Abrysvo, Pfizer) vaccine | NA | **VAERS:** 4 (1.4%) cases out of 281 serious reports |
|  |  |  | RSVPreF3+AS01 (Arexvy, GSK) |  | **VAERS:** 2 (1.2%) cases out of 167 serious reports |
|  |  |  | RSVpreF (Abrysvo, Pfizer) |  | **VAERS:** 2 (2.0%) cases out of 98 serious reports |
|  |  |  | Did not recall/do not know vaccine received |  | **VAERS:** 0 cases out of 16 serious reports |
| Death | | | | | |
| Hause et al. (2024) | Persons >60 years who received an RSV vaccine starting October 20, 2023 | Death within 7 days after vaccination | RSVPreF3+AS01 (Arexvy, GSK) or RSVpreF (Abrysvo, Pfizer) vaccine | NA | **VAERS:** 35 (12.5%) cases out of 281 serious reports |
|  |  |  | RSVPreF3+AS01 (Arexvy, GSK) |  | **VAERS:** 22 (13.2%) cases out of 167 serious reports |
|  |  |  | RSVpreF (Abrysvo, Pfizer) |  | **VAERS:** 11 (11.2%) cases out of 98 serious reports |
|  |  |  | Did not recall/do not know vaccine received |  | **VAERS:** 2 (12.5%) cases out of 16 serious reports |
| Deep venous thrombosis | | | | | |
| Donahue et al. (2025) | Adults >60 years vaccinated with Abrysvo and Arexvy between (August 01, 2023 to September 28, 2024) | Report of deep venous thrombosis within 1-21 days (risk interval) of vaccination | Arexvy (GSK) with same day administration of non-RSV vaccine | Day 43-63 (comparison interval) | 18 reports  RR (95% CI): 0.70 (0.36-1.32) |
|  |  |  | Arexvy (GSK) without same day administration of non-RSV vaccine |  | 37 reports  RR (95% CI): 0.56 (0.35-0.88) |
|  |  |  | Abrysvo (Pfizer) with same day administration of non-RSV vaccine |  | 2 reports  RR (95% CI): 0.64 (0.07-4.83) |
|  |  |  | Abrysvo (Pfizer) without same day administration of non-RSV vaccine |  | 6 reports  RR (95% CI): 0.65 (0.22-1.76) |
| Diarrhea | | | | | |
| Bao et al. (2025) | All eligible individuals (older adults and pregnant people) who received RSVPreF3 (between May 03, 2023, to March 28, 2025), RSVPreF (between May 31, 2023, to March 28, 2025), or mRNA-1345 (between May 31, 2024, to March 28, 2025) | Adverse event report of diarrhea | RSVPreF3 | RSVPreF | **VAERS:** 144 reports  **Reporting odds ratio (ROR):** 1.68 (1.42-1.99) |
|  |  |  | RSVPreF | NA | **VAERS:** 88 reports |
|  |  |  | mRNA-1345 | NA | **VAERS:** 3 reports |
| Domnich et al. (2025) | Persons >60 years who received RSVPreF3 OA between February and  September 2024 | Diarrhea within 7 days after vaccination | RSVPreF3 OA (Arexvy, GSK) | NA | **Mild:** 6 (66.7%) out of 9 adverse reaction reports  **Moderate:** 3 (33.3%) out of 9 adverse reaction reports  **Severe:** 0 out of 9 adverse reaction reports |
| Hause et al. (2024) | Persons >60 years who received an RSV vaccine starting October 20, 2023 | Diarrhea within 7 days after vaccination | RSVPreF3+AS01 (Arexvy, GSK) or RSVpreF (Abrysvo, Pfizer) vaccine | NA | **V-safe surveillance system**: 453 (2.8%) out of 16,220 participants with adverse reactions |
|  |  |  | RSVPreF3+AS01 (Arexvy, GSK) |  | **V-safe surveillance system**: 201 (3.1%) out of 6,402 participants with adverse reactions |
|  |  |  | RSVpreF (Abrysvo, Pfizer) |  | **V-safe surveillance system**: 80 (2.1%) out of 3,882 participants with adverse reactions |
|  |  |  | Did not recall/do not know vaccine received |  | **V-safe surveillance system**: 172 (2.9%) out of 5,936 participants with adverse reactions |
| Discomfort | | | | | |
| Bao et al. (2025) | All eligible individuals (older adults and pregnant people) who received RSVPreF3 (between May 03, 2023, to March 28, 2025), RSVPreF (between May 31, 2023, to March 28, 2025), or mRNA-1345 (between May 31, 2024, to March 28, 2025) | Adverse event report of discomfort | RSVPreF3 | RSVPreF | **VAERS:** 26 reports  **Reporting odds ratio (ROR):** 1.53 (1.03-2.26) |
|  |  |  | RSVPreF | NA | NA |
|  |  |  | mRNA-1345 | NA | NA |
| Dyspnea or cough | | | | | |
| Hause et al. (2024) | Persons >60 years who received an RSV vaccine starting October 20, 2023 | Dyspnea within 7 days after vaccination | RSVPreF3+AS01 (Arexvy, GSK) or RSVpreF (Abrysvo, Pfizer) vaccine | NA | **VAERS:** 5 (1.8%) cases out of 281 serious reports |
|  |  |  | RSVPreF3+AS01 (Arexvy, GSK) |  | **VAERS:** 3 (1.8%) cases out of 167 serious reports |
|  |  |  | RSVpreF (Abrysvo, Pfizer) |  | **VAERS:** 2 (2.0%) cases out of 98 serious reports |
|  |  |  | Did not recall/do not know vaccine received |  | **VAERS:** 0 cases out of 16 serious reports |
| Ecchymosis | | | | | |
| Bao et al. (2025) | All eligible individuals (older adults and pregnant people) who received RSVPreF3 (between May 03, 2023, to March 28, 2025), RSVPreF (between May 31, 2023, to March 28, 2025), or mRNA-1345 (between May 31, 2024, to March 28, 2025) | Adverse event report of ecchymosis | RSVPreF3 | RSVPreF | **VAERS:** 6 reports  **Reporting odds ratio (ROR):** 2.62 (1.15-5.98) |
|  |  |  | RSVPreF | NA | NA |
|  |  |  | mRNA-1345 | NA | NA |
| Encephalitis or aseptic meningitis | | | | | |
| Donahue et al. (2025) | Adults >60 years vaccinated with Abrysvo and Arexvy between (August 01, 2023 to September 28, 2024) | Report of encephalitis within 1-21 days (risk interval) of vaccination | Arexvy (GSK) with same day administration of non-RSV vaccine | Day 43-63 (comparison interval) | 0 reports  RR (95% CI): 0.00 (0.00-4.21) |
|  |  |  | Arexvy (GSK) without same day administration of non-RSV vaccine |  | 2 reports |
|  |  |  | Abrysvo (Pfizer) with same day administration of non-RSV vaccine |  | 1 report |
|  |  |  | Abrysvo (Pfizer) without same day administration of non-RSV vaccine |  | NA |
| Hause et al. (2024) | Persons >60 years who received an RSV vaccine starting October 20, 2023 | Encephalitis within 7 days after vaccination | RSVPreF3+AS01 (Arexvy, GSK) or RSVpreF (Abrysvo, Pfizer) vaccine | NA | **VAERS:** 11 (3.9%) cases out of 281 serious reports |
|  |  |  | RSVPreF3+AS01 (Arexvy, GSK) |  | **VAERS:** 5 (3.0%) cases out of 167 serious reports |
|  |  |  | RSVpreF (Abrysvo, Pfizer) |  | **VAERS:** 5 (5.1%) cases out of 98 serious reports |
|  |  |  | Did not recall/do not know vaccine received |  | **VAERS:** 1 (6.3%) case out of 16 serious reports |
| Erythema (systemic) | | | | | |
| Bao et al. (2025) | All eligible individuals (older adults and pregnant people) who received RSVPreF3 (between May 03, 2023, to March 28, 2025), RSVPreF (between May 31, 2023, to March 28, 2025), or mRNA-1345 (between May 31, 2024, to March 28, 2025) | Adverse event report of systemic erythema | RSVPreF3 | RSVPreF | **VAERS:** 261 reports  **Reporting odds ratio (ROR):** 2.81 (2.48-3.19) |
|  |  |  | RSVPreF | NA | **VAERS:** 93 reports |
|  |  |  | mRNA-1345 | NA | NA |
| Hause et al. (2024) | Persons >60 years who received an RSV vaccine starting October 20, 2023 | Systemic erythema within 7 days after vaccination | RSVPreF3+AS01 (Arexvy, GSK) or RSVpreF (Abrysvo, Pfizer) vaccine | NA | **VAERS:** 384 (13.2%) out of 2,919 reports |
|  |  |  | RSVPreF3+AS01 (Arexvy, GSK) |  | **VAERS:** 186 (9.2%) out of 2,026 reports |
|  |  |  | RSVpreF (Abrysvo, Pfizer) |  | **VAERS:** 57 (6.9%) out of 821 reports |
|  |  |  | Did not recall/do not know vaccine received |  | **VAERS:** 4 (5.6%) out of 72 reports |
| Fatigue/malaise/tiredness | | | | | |
| Bao et al. (2025) | All eligible individuals (older adults and pregnant people) who received RSVPreF3 (between May 03, 2023, to March 28, 2025), RSVPreF (between May 31, 2023, to March 28, 2025), or mRNA-1345 (between May 31, 2024, to March 28, 2025) | Adverse event report of fatigue | RSVPreF3 | RSVPreF | **VAERS:** 428 reports  **Reporting odds ratio (ROR):** 1.53 (1.38-1.68) |
|  |  |  | RSVPreF | NA | **VAERS:** 221 reports |
|  |  |  | mRNA-1345 | NA | NA |
| Domnich et al. (2025) | Persons >60 years who received RSVPreF3 OA between February and  September 2024 | Malaise within 7 days after vaccination. Defined as <38 ◦C, 38–39 ◦C and >39 ◦C for grades mild, moderate and severe respectively | RSVPreF3 OA (Arexvy, GSK) | NA | **Mild:** 28 (57.1%) out of 49 adverse reaction reports  **Moderate:** 18 (36.7%) out of 49 adverse reaction reports  **Severe:** 3 (6.1%) out of 49 adverse reaction reports |
| Hause et al. (2024) | Persons >60 years who received an RSV vaccine starting October 20, 2023 | Report of fatigue/tiredness within 7 days after vaccination | RSVPreF3+AS01 (Arexvy, GSK) or RSVpreF (Abrysvo, Pfizer) vaccine | NA | **V-safe surveillance system**: 3,327 (20.5%) out of 16,220 participants with adverse reactions  **VAERS:** 355 (12.2%) out of 2,919 reports |
|  |  |  | RSVPreF3+AS01 (Arexvy, GSK) |  | **V-safe surveillance system**: 1,640 (25.6%) out of 6,402 participants with adverse reactions  **VAERS:** 235 (11.6%) out of 2,026 reports |
|  |  |  | RSVpreF (Abrysvo, Pfizer) |  | **V-safe surveillance system**: 515 (13.5%) out of 3,882 participants with adverse reactions  **VAERS:** 102 (12.4%) out of 821 reports |
|  |  |  | Did not recall/do not know vaccine received |  | **V-safe surveillance system**: 1,172 (19.7%) out of 5,936 participants with adverse reactions  **VAERS:** 18 (25.0%) out of 72 reports |
| Lotscher et al. (2025) | Adult patients more than 3 months post-allogeneic hematopoietic cell transplant | Report of fatigue | RSVPreF3 OA | NA | 26 reports (out of 82 participants) |
| Nguyen et. al. (2025) | Adults aged 60-69 years in Australia who received Arexvy RSVPreF protein vaccine within February 29, to September 27, 2024 | Fatigue within 3 days of vaccination | All RSVPreF vaccine doses (Arexvy, GSK) | NA | 416 (21%) of 2,013 participants |
|  |  |  | Only RSVPreF vaccine dose |  | 348 (20%) of 1,739 participants |
|  |  |  | RSVPreF vaccine dose received concomitantly |  | 68 (25%) of 274 participants |
| Fever/pyrexia | | | | | |
| Bao et al. (2025) | All eligible individuals (older adults and pregnant people) who received RSVPreF3 (between May 03, 2023, to March 28, 2025), RSVPreF (between May 31, 2023, to March 28, 2025), or mRNA-1345 (between May 31, 2024, to March 28, 2025) | Adverse event report of pyrexia | RSVPreF3 | RSVPreF | **VAERS:** 370 reports  **Reporting odds ratio (ROR):** 1.16 (1.04-1.28) |
|  |  |  | RSVPreF | NA | NA |
|  |  |  | mRNA-1345 | NA | NA |
| Domnich et al. (2025) | Persons >60 years who received RSVPreF3 OA between February and  September 2024 | Fever within 7 days after vaccination. Defined as <38 ◦C, 38–39 ◦C and >39 ◦C for grades mild, moderate and severe respectively | RSVPreF3 OA (Arexvy, GSK) | NA | **Mild:** 33 (97.1%) out of 34 adverse reaction reports  **Moderate:** 1 (2.9%) out of 34 adverse reaction reports  **Severe:** 0 out of 34 adverse reaction reports |
| Hause et al. (2024) | Persons >60 years who received an RSV vaccine starting October 20, 2023 | Self-reported fever within 7 days after vaccination | RSVPreF3+AS01 (Arexvy, GSK) or RSVpreF (Abrysvo, Pfizer) vaccine | NA | **V-safe surveillance system**: 1,765 (10.9%) out of 16,220 participants with adverse reactions  **VAERS:** 275 (9.4%) out of 2,919 reports |
|  |  |  | RSVPreF3+AS01 (Arexvy, GSK) |  | **V-safe surveillance system**: 836 (13.1%) out of 6,402 participants with adverse reactions  **VAERS:** 261 (12.9%) out of 2,026 reports |
|  |  |  | RSVpreF (Abrysvo, Pfizer) |  | **V-safe surveillance system**: 293 (7.5%) out of 3,882 participants with adverse reactions  **VAERS:** 66 (8.0%) out of 821 reports |
|  |  |  | Did not recall/do not know vaccine received |  | **V-safe surveillance system**: 636 (10.7%) out of 5,936 participants with adverse reactions  **VAERS:** 2 (2.8%) out of 72 reports |
| Nguyen et. al. (2025) | Adults aged 60-69 years in Australia who received Arexvy RSVPreF protein vaccine within February 29, to September 27, 2024 | Fever within 3 days of vaccination | All RSVPreF vaccine doses (Arexvy, GSK) | NA | 98 (4.9%) of 2,013 participants |
|  |  |  | Only RSVPreF vaccine dose |  | 81 (4.7%) of 1,739 participants |
|  |  |  | RSVPreF vaccine dose received concomitantly |  | 17 (6.2%) of 274 participants |
| Gastrointestinal event | | | | | |
| Nguyen et. al. (2025) | Adults aged 60-69 years in Australia who received Arexvy RSVPreF protein vaccine within February 29, to September 27, 2024 | Any gastrointestinal adverse event within 3 days of vaccination. Gastrointestinal events include nausea, vomiting, diarrhoea, abdominal pain | All RSVPreF vaccine doses (Arexvy, GSK) | NA | 104 (5.2%) of 2,013 participants |
|  |  |  | Only RSVPreF vaccine dose |  | 86 (4.9%) of 1,739 participants |
|  |  |  | RSVPreF vaccine dose received concomitantly |  | 18 (6.6%) of 274 participants |
| Guillain-Barré syndrome (GBS) | | | | | |
| Bao et al. (2025) | All eligible individuals (older adults and pregnant people) who received RSVPreF3 (between May 03, 2023, to March 28, 2025), RSVPreF (between May 31, 2023, to March 28, 2025), or mRNA-1345 (between May 31, 2024, to March 28, 2025) | Adverse event report of Guillain-Barré syndrome | RSVPreF3 | RSVPreF | **VAERS:** 46 reports  **Reporting odds ratio (ROR):** 2.06 (1.54-2.78) |
|  |  |  | RSVPreF | NA | **VAERS:** 58 reports |
|  |  |  | mRNA-1345 | NA | NA |
| Donahue et al. (2025) | Adults >60 years vaccinated with Abrysvo and Arexvy between (August 01, 2023 to September 28, 2024) | Report of GBS within 1-21 days (risk interval) of vaccination | Arexvy (GSK) with same day administration of non-RSV vaccine | Day 43-63 (comparison interval) | NA |
|  |  |  | Arexvy (GSK) without same day administration of non-RSV vaccine |  | 3 reports  RR (95% CI): 3.58 (0.29-113.17) |
|  |  |  | Abrysvo (Pfizer) with same day administration of non-RSV vaccine |  | NA |
|  |  |  | Abrysvo (Pfizer) without same day administration of non-RSV vaccine |  | 1 report |
| Fry et al. (2025) | Persons ≥60 years who received RSV vaccine between 1 July 2023 and 30 June 2024 | Final diagnosis of GBS (ICD-10 code G61.0) for an inpatient visit within 90 days after vaccination | RSVPreF3+AS01 (Arexvy, GSK) or RSVpreF (Abrysvo, Pfizer) vaccine | Days 1 to 42 after vaccination (risk period) and days 43 to 90 (control period) | IRR 2.1 (95% CI: 1.5-2.9) |
|  |  |  | RSVPreF3+AS01 (Arexvy, GSK) |  | IRR 1.5 (95% CI: 0.9-2.2) |
|  |  |  | RSVpreF (Abrysvo, Pfizer) |  | IRR 2.4 (95% CI: 1.5-4.0) |
| Hause et al. (2024) | Persons >60 years who received an RSV vaccine starting October 20, 2023 | GBS within 7 days after vaccination | RSVPreF3+AS01 (Arexvy, GSK) or RSVpreF (Abrysvo, Pfizer) vaccine | NA | **VAERS:** 18 (10.8%) cases out of 167 serious reports |
|  |  |  | RSVPreF3+AS01 (Arexvy, GSK) |  | **VAERS:** 19 (19.4%) cases out of 98 serious reports |
|  |  |  | RSVpreF (Abrysvo, Pfizer) |  | **VAERS:** 0 cases out of 16 serious reports |
|  |  |  | Did not recall/do not know vaccine received |  | **VAERS:** 37 (13.2%) cases out of 281 serious reports |
| Lloyd et al. (2025) | Medicare FFS and Part D beneficiaries who received RSV vaccine between May 3, 2023 (RSVPreF3+AS01) or May 31, 2023 (RSVPreF3) to January 28, 2024 | Hospital inpatient claim for incident GBS (ICD-10 code G61.0) within 90 days after vaccination | RSVPreF3+AS01 (Arexvy, GSK) | Days 1 to 42 after vaccination (risk period) and days 43 to 90 (control period) | **Seasonality and Farrington adjustment:** IRR 2.90 (95% CI: 1.62-5.21)  **Farrington adjustment:** IRR 2.93 (95% CI: 1.63-5.26)  **Seasonality adjustment:** IRR 2.75 (95% CI: 1.54-4.93)  **Non-seasonality and non-Farrington adjustment:** IRR 2.78 (95% CI: 1.55-4.98) |
|  |  |  | RSVpreF (Abrysvo, Pfizer) vaccine |  | **Seasonality and Farrington adjustment:** IRR 3.02 (95% CI: 1.50-6.08)  **Farrington adjustment:** IRR 2.91 (95% CI: 1.45-5.86)  **Seasonality adjustment:** IRR 2.95 (95% CI: 1.47-5.94)  **Non-seasonality and non-Farrington adjustment:** IRR 2.85 (95% CI: 1.41-5.72) |
|  |  | Chart-confirmed cases for GBS, classified as Brighton Levels 1, 2, or 3 within 90 days after vaccination | RSVPreF3+AS01 (Arexvy, GSK) |  | **Seasonality and Farrington adjustment:** IRR 2.38 (95% CI: 1.07-5.31)  **Farrington adjustment:** IRR 2.36 (95% CI: 1.06-5.25)  **Seasonality adjustment:** IRR 2.30 (95% CI: 1.03-5.12)  **Non-seasonality and non-Farrington adjustment:** IRR 2.28 (95% CI: 1.02-5.07) |
|  |  |  | RSVpreF (Abrysvo, Pfizer) vaccine |  | **Seasonality and Farrington adjustment:** IRR 2.02 (95% CI: 0.88-4.61)  **Farrington adjustment:** IRR 1.91 (95% CI: 0.84-4.37)  **Seasonality adjustment:** IRR 2.01 (95% CI: 0.88-4.60)  **Non-seasonality and non-Farrington adjustment:** IRR 1.90 (95% CI: 0.83-4.35) |
|  |  | PPV-based imputation on unreturned cases | RSVPreF3+AS01 (Arexvy, GSK) |  | **Seasonality and Farrington adjustment:** IRR 2.61 (95% CI: 1.25-5.45)  **Farrington adjustment:** IRR 2.64 (95% CI: 1.26-5.50)  **Seasonality adjustment:** IRR 2.54 (95% CI: 1.22-5.30)  **Non-seasonality and non-Farrington adjustment:** IRR 2.56 (95% CI: 1.23-5.35) |
|  |  |  | RSVpreF (Abrysvo, Pfizer) vaccine |  | **Seasonality and Farrington adjustment:** IRR 2.12 (95% CI: 0.97-4.64)  **Farrington adjustment:** IRR 2.03 (95% CI: 0.93-4.45)  **Seasonality adjustment:** IRR 2.07 (95% CI: 0.94-4.53)  **Non-seasonality and non-Farrington adjustment:** IRR 1.99 (95% CI: 0.91-4.35) |
| Headache | | | | | |
| Bao et al. (2025) | All eligible individuals (older adults and pregnant people) who received RSVPreF3 (between May 03, 2023, to March 28, 2025), RSVPreF (between May 31, 2023, to March 28, 2025), or mRNA-1345 (between May 31, 2024, to March 28, 2025) | Adverse event report of headache | RSVPreF3 | RSVPreF | **VAERS:** 409 reports  **Reporting odds ratio (ROR):** 1.5 (1.36-1.66) |
|  |  |  | RSVPreF | NA | **VAERS:** 231 reports |
|  |  |  | mRNA-1345 | NA | **VAERS:** 5 reports |
| Domnich et al. (2025) | Persons >60 years who received RSVPreF3 OA between February and  September 2024 | Headache within 7 days after vaccination | RSVPreF3 OA (Arexvy, GSK) | NA | **Mild:** 21 (63.6%) out of 33 adverse reaction reports  **Moderate:** 9 (27.3%) out of 33 adverse reaction reports  **Severe:** 3 (9.1%) out of 33 adverse reaction reports |
| Hause et al. (2024) | Persons >60 years who received an RSV vaccine starting October 20, 2023 | Headache within 7 days after vaccination | RSVPreF3+AS01 (Arexvy, GSK) or RSVpreF (Abrysvo, Pfizer) vaccine | NA | **V-safe surveillance system**: 2,460 (15.2%) out of 16,220 participants with adverse reactions  **VAERS:** 376 (12.9%) out of 2,919 reports |
|  |  |  | RSVPreF3+AS01 (Arexvy, GSK) |  | **V-safe surveillance system**: 1,227 (19.2%) out of 6,402 participants with adverse reactions  **VAERS:** 261 (12.9%) out of 2,026 reports |
|  |  |  | RSVpreF (Abrysvo, Pfizer) |  | **V-safe surveillance system**: 413 (10.6%) out of 3,882 participants with adverse reactions  **VAERS:** 105 (12.8%) out of 821 reports |
|  |  |  | Did not recall/do not know vaccine received |  | **V-safe surveillance system**: 820 (13.8%) out of 5,936 participants with adverse reactions  **VAERS:** 10 (13.9%) out of 72 reports |
| Nguyen et. al. (2025) | Adults aged 60-69 years in Australia who received Arexvy RSVPreF protein vaccine within February 29, to September 27, 2024 | Headache within 3 days of vaccination | All RSVPreF vaccine doses (Arexvy, GSK) | NA | 237 (12%) of 2,013 participants |
|  |  |  | Only RSVPreF vaccine dose |  | 198 (11%) of 1,739 participants |
|  |  |  | RSVPreF vaccine dose received concomitantly |  | 39 (14%) of 274 participants |
| Hyperglycemia | | | | | |
| Bao et al. (2025) | All eligible individuals (older adults and pregnant people) who received RSVPreF3 (between May 03, 2023, to March 28, 2025), RSVPreF (between May 31, 2023, to March 28, 2025), or mRNA-1345 (between May 31, 2024, to March 28, 2025) | Adverse event report of increased blood glucose | RSVPreF3 | RSVPreF | **VAERS:** 14 reports  **Reporting odds ratio (ROR):** 2.18 (1.27-3.73) |
|  |  |  | RSVPreF | NA | NA |
|  |  |  | mRNA-1345 | NA | NA |
| Immune thrombocytopenia/idiopathic thrombocytopenic purpura (ITP) | | | | | |
| Donahue et al. (2025) | Adults >60 years vaccinated with Abrysvo and Arexvy between (August 01, 2023 to September 28, 2024) | Report of ITP within 1-21 days (risk interval) of vaccination | Arexvy (GSK) with same day administration of non-RSV vaccine | Day 43-63 (comparison interval) | 3 reports  RR (95% CI): 1.57 (0.18-14.97) |
|  |  |  | Arexvy (GSK) without same day administration of non-RSV vaccine |  | 20 reports  RR (95% CI): 1.75 (0.78-4.09) |
|  |  |  | Abrysvo (Pfizer) with same day administration of non-RSV vaccine |  | 0 reports  RR (95% CI): 0.00 (0.00-79.45) |
|  |  |  | Abrysvo (Pfizer) without same day administration of non-RSV vaccine |  | 2 reports  RR (95% CI): 1.16 (0.11-11.78) |
| Fry et al. (2025) | Persons ≥60 years who received RSV vaccine between 1 July 2023 and 30 June 2024 | Encounter diagnosis of idiopathic thrombocytopenic purpura (ICD-10 code D69.3) within 90 days after vaccination | RSVPreF3+AS01 (Arexvy, GSK) or RSVpreF (Abrysvo, Pfizer) vaccine | Incidence rates were compared between days 1 to 42 after vaccination (risk  period) and days 43 to 90 (control period) | IRR 1.0 (95% CI: 0.9-1.2) |
|  |  |  | RSVPreF3+AS01 (Arexvy, GSK) |  | IRR 1.1 (95% CI: 8.7-1.3) |
|  |  |  | RSVpreF (Abrysvo, Pfizer) |  | IRR 0.9 (95% CI: 0.7-1.2) |
| Hause et al. (2024) | Persons >60 years who received an RSV vaccine starting October 20, 2023 | Immune thrombocytopenia within 7 days after vaccination | RSVPreF3+AS01 (Arexvy, GSK) or RSVpreF (Abrysvo, Pfizer) vaccine | NA | **VAERS:** 11 (3.9%) cases out of 281 serious reports |
|  |  |  | RSVPreF3+AS01 (Arexvy, GSK) |  | **VAERS:** 5 (3.0%) cases out of 167 serious reports |
|  |  |  | RSVpreF (Abrysvo, Pfizer) |  | **VAERS:** 6 (6.1%) cases out of 98 serious reports |
|  |  |  | Did not recall/do not know vaccine received |  | **VAERS:** 0 cases out of 16 serious reports |
| Induration | | | | | |
| Bao et al. (2025) | All eligible individuals (older adults and pregnant people) who received RSVPreF3 (between May 03, 2023, to March 28, 2025), RSVPreF (between May 31, 2023, to March 28, 2025), or mRNA-1345 (between May 31, 2024, to March 28, 2025) | Adverse event report of induration | RSVPreF3 | RSVPreF | **VAERS:** 14 reports  **Reporting odds ratio (ROR):** 1.81 (1.06-3.08) |
|  |  |  | RSVPreF | NA | NA |
|  |  |  | mRNA-1345 | NA | NA |
| Lethargy | | | | | |
| Bao et al. (2025) | All eligible individuals (older adults and pregnant people) who received RSVPreF3 (between May 03, 2023, to March 28, 2025), RSVPreF (between May 31, 2023, to March 28, 2025), or mRNA-1345 (between May 31, 2024, to March 28, 2025) | Adverse event report of lethargy | RSVPreF3 | RSVPreF | **VAERS:** 72 reports  **Reporting odds ratio (ROR):** 1.45 (1.15-1.84) |
|  |  |  | RSVPreF | NA | NA |
|  |  |  | mRNA-1345 | NA | NA |
| Lymphadenopathy | | | | | |
| Domnich et al. (2025) | Persons >60 years who received RSVPreF3 OA between February and  September 2024 | Lymphadenopathy within 7 days after vaccination | RSVPreF3 OA (Arexvy, GSK) | NA | 2 events reported. Intensity was not reported |
| Mobility (reduced) | | | | | |
| Bao et al. (2025) | All eligible individuals (older adults and pregnant people) who received RSVPreF3 (between May 03, 2023, to March 28, 2025), RSVPreF (between May 31, 2023, to March 28, 2025), or mRNA-1345 (between May 31, 2024, to March 28, 2025) | Adverse event report of decreased mobility | RSVPreF3 | RSVPreF | **VAERS:** 116 reports  **Reporting odds ratio (ROR):** 2.23 (1.85-2.69) |
|  |  |  | RSVPreF | NA | **VAERS:** 66 reports |
|  |  |  | mRNA-1345 | NA | NA |
| Myalgia | | | | | |
| Bao et al. (2025) | All eligible individuals (older adults and pregnant people) who received RSVPreF3 (between May 03, 2023, to March 28, 2025), RSVPreF (between May 31, 2023, to March 28, 2025), or mRNA-1345 (between May 31, 2024, to March 28, 2025) | Adverse event report of myalgia | RSVPreF3 | RSVPreF | **VAERS:** 225 reports  **Reporting odds ratio (ROR):** 1.76 (1.54-2.01) |
|  |  |  | RSVPreF | NA | **VAERS:** 113 reports |
|  |  |  | mRNA-1345 | NA | NA |
| Domnich et al. (2025) | Persons >60 years who received RSVPreF3 OA between February and  September 2024 | Myalgia within 7 days after vaccination | RSVPreF3 OA (Arexvy, GSK) | NA | **Mild:** 17 (58.6%) out of 29 adverse reaction reports  **Moderate:** 8 (27.6%) out of 29 adverse reaction reports  **Severe:** 4 (13.8%) out of 29 adverse reaction reports |
| Hause et al. (2024) | Persons >60 years who received an RSV vaccine starting October 20, 2023 | Muscle or body ache within 7 days after vaccination | RSVPreF3+AS01 (Arexvy, GSK) or RSVpreF (Abrysvo, Pfizer) vaccine | NA | **V-safe surveillance system**: 2,843 (17.5%) out of 16,220 participants with adverse reactions |
|  |  |  | RSVPreF3+AS01 (Arexvy, GSK) |  | **V-safe surveillance system**: 1,407 (22.0%) out of 6,402 participants with adverse reactions |
|  |  |  | RSVpreF (Abrysvo, Pfizer) |  | **V-safe surveillance system**: 484 (14.1%) out of 3,882 participants with adverse reactions |
|  |  |  | Did not recall/do not know vaccine received |  | **V-safe surveillance system**: 952 (16.0%) out of 5,936 participants with adverse reactions |
| Lotscher et al. (2025) | Adult patients more than 3 months post-allogeneic hematopoietic cell transplant | Report of myalgia | RSVPreF3 OA | NA | 23 reports (out of 82 participants) |
| Nguyen et. al. (2025) | Adults aged 60-69 years in Australia who received Arexvy RSVPreF protein vaccine within February 29, to September 27, 2024 | Myalgia within 3 days of vaccination | All RSVPreF vaccine doses (Arexvy, GSK) | NA | 252 (13%) of 2,013 participants |
|  |  |  | Only RSVPreF vaccine dose |  | 199 (11%) of 1,739 participants |
|  |  |  | RSVPreF vaccine dose received concomitantly |  | 53 (19%) of 274 participants |
| Myocardial infarction | | | | | |
| Donahue et al. (2025) | Adults >60 years vaccinated with Abrysvo and Arexvy between (August 01, 2023 to September 28, 2024) | Report of acute myocardial infarction within 1-21 days (risk interval) of vaccination | Arexvy (GSK) with same day administration of non-RSV vaccine | Day 43-63 (comparison interval) | 49 reports  RR (95% CI): 1.19 (0.76-1.87) |
|  |  |  | Arexvy (GSK) without same day administration of non-RSV vaccine |  | 106 reports  RR (95% CI): 0.99 (0.74-1.34) |
|  |  |  | Abrysvo (Pfizer) with same day administration of non-RSV vaccine |  | 10 reports  RR (95% CI): 1.13 (0.39-3.33) |
|  |  |  | Abrysvo (Pfizer) without same day administration of non-RSV vaccine |  | 17 reports  RR (95% CI): 1.11 (0.51-2.36) |
| Hause et al. (2024) | Persons >60 years who received an RSV vaccine starting October 20, 2023 | Myocardial infarction within 7 days after vaccination | RSVPreF3+AS01 (Arexvy, GSK) or RSVpreF (Abrysvo, Pfizer) vaccine | NA | **VAERS:** 4 (1.4%) cases out of 281 serious reports |
|  |  |  | RSVPreF3+AS01 (Arexvy, GSK) |  | **VAERS:** 3 (1.8%) cases out of 167 serious reports |
|  |  |  | RSVpreF (Abrysvo, Pfizer) |  | **VAERS:** 1 (1.0%) case out of 98 serious reports |
|  |  |  | Did not recall/do not know vaccine received |  | **VAERS:** 0 cases out of 16 serious reports |
| Nausea | | | | | |
| Bao et al. (2025) | All eligible individuals (older adults and pregnant people) who received RSVPreF3 (between May 03, 2023, to March 28, 2025), RSVPreF (between May 31, 2023, to March 28, 2025), or mRNA-1345 (between May 31, 2024, to March 28, 2025) | Adverse event report of nausea | RSVPreF3 | RSVPreF | **VAERS:** 210 reports  **Reporting odds ratio (ROR):** 1.24 (1.08-1.43) |
|  |  |  | RSVPreF | NA | **VAERS:** 122 reports |
|  |  |  | mRNA-1345 | NA | **VAERS:** 4 reports |
| Domnich et al. (2025) | Persons >60 years who received RSVPreF3 OA between February and  September 2024 | Nausea within 7 days after vaccination | RSVPreF3 OA (Arexvy, GSK) | NA | **Mild:** 5 (50.0%) out of 10 adverse reaction reports  **Moderate:** 5 (50.0%) out of 10 adverse reaction reports  **Severe:** 0 out of 10 adverse reaction reports |
| Hause et al. (2024) | Persons >60 years who received an RSV vaccine starting October 20, 2023 | Nausea within 7 days after vaccination | RSVPreF3+AS01 (Arexvy, GSK) or RSVpreF (Abrysvo, Pfizer) vaccine | NA | **V-safe surveillance system**: 689 (4.2%) out of 16,220 participants with adverse reactions |
|  |  |  | RSVPreF3+AS01 (Arexvy, GSK) |  | **V-safe surveillance system**: 317 (5.0%) out of 6,402 participants with adverse reactions |
|  |  |  | RSVpreF (Abrysvo, Pfizer) |  | **V-safe surveillance system**: 123 (3.2%) out of 3,882 participants with adverse reactions |
|  |  |  | Did not recall/do not know vaccine received |  | **V-safe surveillance system**: 249 (4.2%) out of 5,936 participants with adverse reactions |
| Nodule | | | | | |
| Bao et al. (2025) | All eligible individuals (older adults and pregnant people) who received RSVPreF3 (between May 03, 2023, to March 28, 2025), RSVPreF (between May 31, 2023, to March 28, 2025), or mRNA-1345 (between May 31, 2024, to March 28, 2025) | Adverse event report of nodule formation | RSVPreF3 | RSVPreF | **VAERS:** 15 reports  **Reporting odds ratio (ROR):** 4.83 (2.83-8.24) |
|  |  |  | RSVPreF | NA | NA |
|  |  |  | mRNA-1345 | NA | NA |
| Li et al. (2025) | Adults older than >60 years age who received RSV vaccine between May3, 2023 and December 27, 2024 | Report of tenderness | RSVPreF3+AS01 (Arexvy, GSK) or RSVpreF (Abrysvo, Pfizer) vaccine | NA | 17 reports  EBGM: 3.41, EMGB05: 2.11 |
| Pain | | | | | |
| Bao et al. (2025) | All eligible individuals (older adults and pregnant people) who received RSVPreF3 (between May 03, 2023, to March 28, 2025), RSVPreF (between May 31, 2023, to March 28, 2025), or mRNA-1345 (between May 31, 2024, to March 28, 2025) | Adverse event report of pain | RSVPreF3 | RSVPreF | **VAERS:** 466 reports  **Reporting odds ratio (ROR):** 2.19 (1.99-2.4) |
|  |  |  | RSVPreF | NA | **VAERS:** 170 reports |
|  |  |  | mRNA-1345 | NA | **VAERS:** 5 reports |
| Hause et al. (2024) | Persons >60 years who received an RSV vaccine starting October 20, 2023 | Systemic pain within 7 days after vaccination | RSVPreF3+AS01 (Arexvy, GSK) or RSVpreF (Abrysvo, Pfizer) vaccine | NA | **VAERS:** 373 (12.8%) out of 2,919 reports |
|  |  |  | RSVPreF3+AS01 (Arexvy, GSK) |  | **VAERS:** 276 (13.6%) out of 2,026 reports |
|  |  |  | RSVpreF (Abrysvo, Pfizer) |  | **VAERS:** 85 (10.4%) out of 821 reports |
|  |  |  | Did not recall/do not know vaccine received |  | **VAERS:** 12 (16.7%) out of 72 reports |
| Pain in extremity | | | | | |
| Bao et al. (2025) | All eligible individuals (older adults and pregnant people) who received RSVPreF3 (between May 03, 2023, to March 28, 2025), RSVPreF (between May 31, 2023, to March 28, 2025), or mRNA-1345 (between May 31, 2024, to March 28, 2025) | Adverse event report of pain in extremity | RSVPreF3 | RSVPreF | **VAERS:** 458 reports  **Reporting odds ratio (ROR):** 2.44 (2.22-2.68) |
|  |  |  | RSVPreF | NA | **VAERS:** 182 reports |
|  |  |  | mRNA-1345 | NA | NA |
| Hause et al. (2024) | Persons >60 years who received an RSV vaccine starting October 20, 2023 | Pain in extremity within 7 days after vaccination | RSVPreF3+AS01 (Arexvy, GSK) or RSVpreF (Abrysvo, Pfizer) vaccine | NA | **VAERS:** 384 (13.2%) out of 2,919 reports |
|  |  |  | RSVPreF3+AS01 (Arexvy, GSK) |  | **VAERS:** 282 (13.9%) out of 2,026 reports |
|  |  |  | RSVpreF (Abrysvo, Pfizer) |  | **VAERS:** 94 (11.4%) out of 821 reports |
|  |  |  | Did not recall/do not know vaccine received |  | **VAERS:** 8 (11.1%) out of 72 reports |
| Pericarditis | | | | | |
| Donahue et al. (2025) | Adults >60 years vaccinated with Abrysvo and Arexvy between (August 01, 2023 to September 28, 2024) | Report of pericarditis within 1-21 days (risk interval) of vaccination | Arexvy (GSK) with same day administration of non-RSV vaccine | Day 43-63 (comparison interval) | 1 report  RR (95% CI): 0.22 (0.01-3.36) |
|  |  |  | Arexvy (GSK) without same day administration of non-RSV vaccine |  | 2 reports  RR (95% CI): 0.26 (0.01-3.17) |
|  |  |  | Abrysvo (Pfizer) with same day administration of non-RSV vaccine |  | 1 report  RR (95% CI): NA |
|  |  |  | Abrysvo (Pfizer) without same day administration of non-RSV vaccine |  | NA |
| Pneumonia | | | | | |
| Hause et al. (2024) | Persons >60 years who received an RSV vaccine starting October 20, 2023 | Pneumonia within 7 days after vaccination | RSVPreF3+AS01 (Arexvy, GSK) or RSVpreF (Abrysvo, Pfizer) vaccine | NA | **VAERS:** 9 (3.2%) cases out of 281 serious reports |
|  |  |  | RSVPreF3+AS01 (Arexvy, GSK) |  | **VAERS:** 5 (3.0%) cases out of 167 serious reports |
|  |  |  | RSVpreF (Abrysvo, Pfizer) |  | **VAERS:** 3 (3.1%) cases out of 98 serious reports |
|  |  |  | Did not recall/do not know vaccine received |  | **VAERS:** 1 (6.3%) case out of 16 serious reports |
| Pruritus (generalised) | | | | | |
| Bao et al. (2025) | All eligible individuals (older adults and pregnant people) who received RSVPreF3 (between May 03, 2023, to March 28, 2025), RSVPreF (between May 31, 2023, to March 28, 2025), or mRNA-1345 (between May 31, 2024, to March 28, 2025) | Adverse event report of generalised pruritus | RSVPreF3 | RSVPreF | **VAERS:** 158 reports  **Reporting odds ratio (ROR):** 1.75 (1.5-2.06) |
|  |  |  | RSVPreF | NA | **VAERS:** 90 reports |
|  |  |  | mRNA-1345 | NA | NA |
| Domnich et al. (2025) | Persons >60 years who received RSVPreF3 OA between February and  September 2024 | Generalised pruritus within 7 days after vaccination | RSVPreF3 OA (Arexvy, GSK) | NA | **Mild:** 3 (60.0%) out of 5 adverse reaction reports  **Moderate:** 2 (40.0%) out of 5 adverse reaction reports  **Severe:** 0 out of 5 adverse reaction reports |
| Pulmonary embolism | | | | | |
| Donahue et al. (2025) | Adults >60 years vaccinated with Abrysvo and Arexvy between (August 01, 2023 to September 28, 2024) | Report of pulmonary embolism within 1-21 days (risk interval) of vaccination | Arexvy (GSK) with same day administration of non-RSV vaccine | Day 43-63 (comparison interval) | 9 reports  RR (95% CI): 0.54 (0.21-1.29) |
|  |  |  | Arexvy (GSK) without same day administration of non-RSV vaccine |  | 39 reports  RR (95% CI): 1.23 (0.75-2.03) |
|  |  |  | Abrysvo (Pfizer) with same day administration of non-RSV vaccine |  | 0 reports  RR (95% CI): 0.00 (0.00-1.87) |
|  |  |  | Abrysvo (Pfizer) without same day administration of non-RSV vaccine |  | 6 reports  RR (95% CI): 1.32 (0.29-6.14) |
| Rash | | | | | |
| Bao et al. (2025) | All eligible individuals (older adults and pregnant people) who received RSVPreF3 (between May 03, 2023, to March 28, 2025), RSVPreF (between May 31, 2023, to March 28, 2025), or mRNA-1345 (between May 31, 2024, to March 28, 2025) | Adverse event report of rash | RSVPreF3 | RSVPreF | **VAERS:** 207 reports  **Reporting odds ratio (ROR):** 1.49 (1.3-1.71) |
|  |  |  | RSVPreF | NA | NA |
|  |  |  | mRNA-1345 | NA | **VAERS:** 4 reports |
| Domnich et al. (2025) | Persons >60 years who received RSVPreF3 OA between February and  September 2024 | Rash within 7 days after vaccination | RSVPreF3 OA (Arexvy, GSK) | NA | **Mild:** 1 (20.0%) out of 5 adverse reaction reports  **Moderate:** 4 (80.0%) out of 5 adverse reaction reports  **Severe:** 0 out of 5 adverse reaction reports |
| Hause et al. (2024) | Persons >60 years who received an RSV vaccine starting October 20, 2023 | Rash at site other than injection site within 7 days after vaccination | RSVPreF3+AS01 (Arexvy, GSK) or RSVpreF (Abrysvo, Pfizer) vaccine | NA | **V-safe surveillance system**: 82 (0.5%) out of 16,220 participants with adverse reactions  **VAERS:** 4 (1.4%) cases out of 281 serious reports |
|  |  |  | RSVPreF3+AS01 (Arexvy, GSK) |  | **V-safe surveillance system**: 32 (0.5%) out of 6,402 participants with adverse reactions  **VAERS:** 1 (0.6%) case out of 167 serious reports |
|  |  |  | RSVpreF (Abrysvo, Pfizer) |  | **V-safe surveillance system**: 20 (0.5%) out of 3,882 participants with adverse reactions  **VAERS:** 2 (2.0%) cases out of 98 serious reports |
|  |  |  | Did not recall/do not know vaccine received |  | **V-safe surveillance system**: 30 (0.5%) out of 5,936 participants with adverse reactions  **VAERS:** 1 (6.3%) case out of 16 serious reports |
| RSV infection | | | | | |
| Hause et al. (2024) | Persons >60 years who received an RSV vaccine starting October 20, 2023 | RSV infection within 7 days after vaccination | RSVPreF3+AS01 (Arexvy, GSK) or RSVpreF (Abrysvo, Pfizer) vaccine | NA | **VAERS:** 5 (1.8%) cases out of 281 serious reports |
|  |  |  | RSVPreF3+AS01 (Arexvy, GSK) |  | **VAERS:** 3 (1.8%) cases out of 167 serious reports |
|  |  |  | RSVpreF (Abrysvo, Pfizer) |  | **VAERS:** 2 (2.0%) cases out of 98 serious reports |
|  |  |  | Did not recall/do not know vaccine received |  | **VAERS:** 0 cases out of 16 serious reports |
| Sepsis, bacteremia, or both | | | | | |
| Hause et al. (2024) | Persons >60 years who received an RSV vaccine starting October 20, 2023 | Sepsis, bacteremia, or both within 7 days after vaccination | RSVPreF3+AS01 (Arexvy, GSK) or RSVpreF (Abrysvo, Pfizer) vaccine | NA | **VAERS:** 11 (3.9%) cases out of 281 serious reports |
|  |  |  | RSVPreF3+AS01 (Arexvy, GSK) |  | **VAERS:** 6 (3.6%) cases out of 167 serious reports |
|  |  |  | RSVpreF (Abrysvo, Pfizer) |  | **VAERS:** 5 (5.1%) cases out of 98 serious reports |
|  |  |  | Did not recall/do not know vaccine received |  | **VAERS:** 0 cases out of 16 serious reports |
| Shoulder pain | | | | | |
| Hause et al. (2024) | Persons >60 years who received an RSV vaccine starting October 20, 2023 | Shoulder pain within 7 days after vaccination | RSVPreF3+AS01 (Arexvy, GSK) or RSVpreF (Abrysvo, Pfizer) vaccine | NA | **VAERS:** 11 (3.9%) cases out of 281 serious reports |
|  |  |  | RSVPreF3+AS01 (Arexvy, GSK) |  | **VAERS:** 7 (4.2%) cases out of 167 serious reports |
|  |  |  | RSVpreF (Abrysvo, Pfizer) |  | **VAERS:** 1 (1.0%) case out of 98 serious reports |
|  |  |  | Did not recall/do not know vaccine received |  | **VAERS:** 3 (18.8%) cases out of 16 serious reports |
| Skin swelling | | | | | |
| Bao et al. (2025) | All eligible individuals (older adults and pregnant people) who received RSVPreF3 (between May 03, 2023, to March 28, 2025), RSVPreF (between May 31, 2023, to March 28, 2025), or mRNA-1345 (between May 31, 2024, to March 28, 2025) | Adverse event report of peripheral swelling | RSVPreF3 | RSVPreF | **VAERS:** 9 reports  **Reporting odds ratio (ROR):** 4.48 (2.25-8.9) |
|  |  |  | RSVPreF | NA | **VAERS:** 42 reports |
|  |  |  | mRNA-1345 | NA | NA |
| Li et al. (2025) | Adults older than >60 years age who received RSV vaccine between May3, 2023 and December 27, 2024 | Report of tenderness | RSVPreF3+AS01 (Arexvy, GSK) or RSVpreF (Abrysvo, Pfizer) vaccine | NA | 10 reports  EBGM: 4.51, EMGB05: 2.29 |
| Sleep disorder | | | | | |
| Bao et al. (2025) | All eligible individuals (older adults and pregnant people) who received RSVPreF3 (between May 03, 2023, to March 28, 2025), RSVPreF (between May 31, 2023, to March 28, 2025), or mRNA-1345 (between May 31, 2024, to March 28, 2025) | Adverse event report of sleep disorder | RSVPreF3 | RSVPreF | **VAERS:** 71 reports  **Reporting odds ratio (ROR):** 1.64 (1.29-2.08) |
|  |  |  | RSVPreF | NA | **VAERS:** 38 reports |
|  |  |  | mRNA-1345 | NA | NA |
| Stroke or transient ischemic attack | | | | | |
| Donahue et al. (2025) | Adults >60 years vaccinated with Abrysvo and Arexvy between (August 01, 2023 to September 28, 2024) | Report of stroke within 1-21 days (risk interval) of vaccination | Arexvy (GSK) with same day administration of non-RSV vaccine | Day 43-63 (comparison interval) | 54 reports  RR (95% CI): 1.27 (0.81-2.01) |
|  |  |  | Arexvy (GSK) without same day administration of non-RSV vaccine |  | 104 reports  RR (95% CI): 0.91 (0.68-1.22) |
|  |  |  | Abrysvo (Pfizer) with same day administration of non-RSV vaccine |  | 7 reports  RR (95% CI): 2.27 (0.46-13.83) |
|  |  |  | Abrysvo (Pfizer) without same day administration of non-RSV vaccine |  | 19 reports  RR (95% CI): 0.72 (0.31-1.66) |
| Hause et al. (2024) | Persons >60 years who received an RSV vaccine starting October 20, 2023 | Stroke or transient ischemic attack within 7 days after vaccination | RSVPreF3+AS01 (Arexvy, GSK) or RSVpreF (Abrysvo, Pfizer) vaccine | NA | **VAERS:** 24 (8.5%) cases out of 281 serious reports |
|  |  |  | RSVPreF3+AS01 (Arexvy, GSK) |  | **VAERS:** 13 (7.8%) cases out of 167 serious reports |
|  |  |  | RSVpreF (Abrysvo, Pfizer) |  | **VAERS:** 10 (10.2%) cases out of 98 serious reports |
|  |  |  | Did not recall/do not know vaccine received |  | **VAERS:** 1 (6.3%) case out of 16 serious reports |
| Syncope | | | | | |
| Hause et al. (2024) | Persons >60 years who received an RSV vaccine starting October 20, 2023 | Syncope within 7 days after vaccination | RSVPreF3+AS01 (Arexvy, GSK) or RSVpreF (Abrysvo, Pfizer) vaccine | NA | **VAERS:** 7 (2.5%) cases out of 281 serious reports |
|  |  |  | RSVPreF3+AS01 (Arexvy, GSK) |  | **VAERS:** 6 (3.6%) cases out of 167 serious reports |
|  |  |  | RSVpreF (Abrysvo, Pfizer) |  | **VAERS:** 1 (1.0%) case out of 98 serious reports |
|  |  |  | Did not recall/do not know vaccine received |  | **VAERS:** 0 cases out of 16 serious reports |
| Tenderness | | | | | |
| Bao et al. (2025) | All eligible individuals (older adults and pregnant people) who received RSVPreF3 (between May 03, 2023, to March 28, 2025), RSVPreF (between May 31, 2023, to March 28, 2025), or mRNA-1345 (between May 31, 2024, to March 28, 2025) | Adverse event report of tenderness | RSVPreF3 | RSVPreF | **VAERS:** 53 reports  **Reporting odds ratio (ROR):** 3.6 (2.72-4.76) |
|  |  |  | RSVPreF | NA | **VAERS:** 24 reports |
|  |  |  | mRNA-1345 | NA | NA |
| Li et al. (2025) | Adults older than >60 years age who received RSV vaccine between May3, 2023 and December 27, 2024 | Report of tenderness | RSVPreF3+AS01 (Arexvy, GSK) or RSVpreF (Abrysvo, Pfizer) vaccine | NA | 65 reports  EBGM: 2.96, EMGB05: 2.33 |
| Thromboembolic event | | | | | |
| Hause et al. (2024) | Persons >60 years who received an RSV vaccine starting October 20, 2023 | Thromboembolic event within 7 days after vaccination | RSVPreF3+AS01 (Arexvy, GSK) or RSVpreF (Abrysvo, Pfizer) vaccine | NA | **VAERS:** 13 (4.6%) cases out of 281 serious reports |
|  |  |  | RSVPreF3+AS01 (Arexvy, GSK) |  | **VAERS:** 7 (4.2%) cases out of 167 serious reports |
|  |  |  | RSVpreF (Abrysvo, Pfizer) |  | **VAERS:** 4 (4.1%) cases out of 98 serious reports |
|  |  |  | Did not recall/do not know vaccine received |  | **VAERS:** 2 (12.5%) cases out of 16 serious reports |
| Transverse myelitis | | | | | |
| Donahue et al. (2025) | Adults >60 years vaccinated with Abrysvo and Arexvy between (August 01, 2023 to September 28, 2024) | Report of transverse myelitis within 1-21 days (risk interval) of vaccination | Arexvy (GSK) with same day administration of non-RSV vaccine | Day 43-63 (comparison interval) | NA |
|  |  |  | Arexvy (GSK) without same day administration of non-RSV vaccine |  | NA |
|  |  |  | Abrysvo (Pfizer) with same day administration of non-RSV vaccine |  | 1 report  RR (95% CI): NA |
|  |  |  | Abrysvo (Pfizer) without same day administration of non-RSV vaccine |  | NA |
| Hause et al. (2024) | Persons >60 years who received an RSV vaccine starting October 20, 2023 | Transverse myelitis within 7 days after vaccination | RSVPreF3+AS01 (Arexvy, GSK) or RSVpreF (Abrysvo, Pfizer) vaccine | NA | **VAERS:** 3 (1.1%) cases out of 281 serious reports |
|  |  |  | RSVPreF3+AS01 (Arexvy, GSK) |  | **VAERS:** 2 (1.2%) cases out of 167 serious reports |
|  |  |  | RSVpreF (Abrysvo, Pfizer) |  | **VAERS:** 1 (1.0%) case out of 98 serious reports |
|  |  |  | Did not recall/do not know vaccine received |  | **VAERS:** 0 cases out of 16 serious reports |
| Vomiting | | | | | |
| Domnich et al. (2025) | Persons >60 years who received RSVPreF3 OA between February and  September 2024 | Vomiting within 7 days after vaccination | RSVPreF3 OA (Arexvy, GSK) | NA | **Mild:** 1 (50.0%) out of 2 adverse reaction reports  **Moderate:** 1 (50.0%) out of 2 adverse reaction reports  **Severe:** 0 out of 2 adverse reaction reports |
| Hause et al. (2024) | Persons >60 years who received an RSV vaccine starting October 20, 2023 | Vomiting within 7 days after vaccination | RSVPreF3+AS01 (Arexvy, GSK) or RSVpreF (Abrysvo, Pfizer) vaccine | NA | **V-safe surveillance system**: 74 (0.5%) out of 16,220 participants with adverse reactions |
|  |  |  | RSVPreF3+AS01 (Arexvy, GSK) |  | **V-safe surveillance system**: 25 (0.4%) out of 6,402 participants with adverse reactions |
|  |  |  | RSVpreF (Abrysvo, Pfizer) |  | **V-safe surveillance system**: 13 (0.3%) out of 3,882 participants with adverse reactions |
|  |  |  | Did not recall/do not know vaccine received |  | **V-safe surveillance system**: 36 (0.6%) out of 5,936 participants with adverse reactions |
| Other | | | | | |
| Hause et al. (2024) | Persons >60 years who received an RSV vaccine starting October 20, 2023 | Other symptoms within 7 days after vaccination. These included sore throat, dizziness, runny nose, cough, dizziness upon standing, congestion. | RSVPreF3+AS01 (Arexvy, GSK) or RSVpreF (Abrysvo, Pfizer) vaccine | NA | **V-safe surveillance system**: 514 (3.2%) out of 16,220 participants with adverse reactions |
|  |  |  | RSVPreF3+AS01 (Arexvy, GSK) |  | **V-safe surveillance system**: 248 (3.9%) out of 6,402 participants with adverse reactions |
|  |  |  | RSVpreF (Abrysvo, Pfizer) |  | **V-safe surveillance system**: 98 (2.5%) out of 3,882 participants with adverse reactions |
|  |  |  | Did not recall/do not know vaccine received |  | **V-safe surveillance system**: 168 (2.8%) out of 5,936 participants with adverse reactions |
| Nguyen et. al. (2025) | Adults aged 60-69 years in Australia who received Arexvy RSVPreF protein vaccine within February 29, to September 27, 2024 | Other events include symptoms which were not included previously, within 3 days of vaccination | All RSVPreF vaccine doses (Arexvy, GSK) | NA | 65 (3.2%) of 2,013 participants |
|  |  |  | Only RSVPreF vaccine dose |  | 57 (3.3%) of 1,739 participants |
|  |  |  | RSVPreF vaccine dose received concomitantly |  | 8 (2.9%) of 274 participants |

Notes:

***Abbreviations****: CI: Confidence interval, EBGM: empirical Bayesian geometric mean, EBGM05: lower bound of the 90 % confidence interval surrounding the empirical Bayesian geometric mean, GBS: Guillain-Barré Syndrome, IRR: incidence rate ratio, PPV: positive predictive value, RR: risk ratio, RSV: respiratory syncytial virus, VAERS: Vaccine Adverse Event Reporting System*

# Publication bias assessment

(1) Uptake


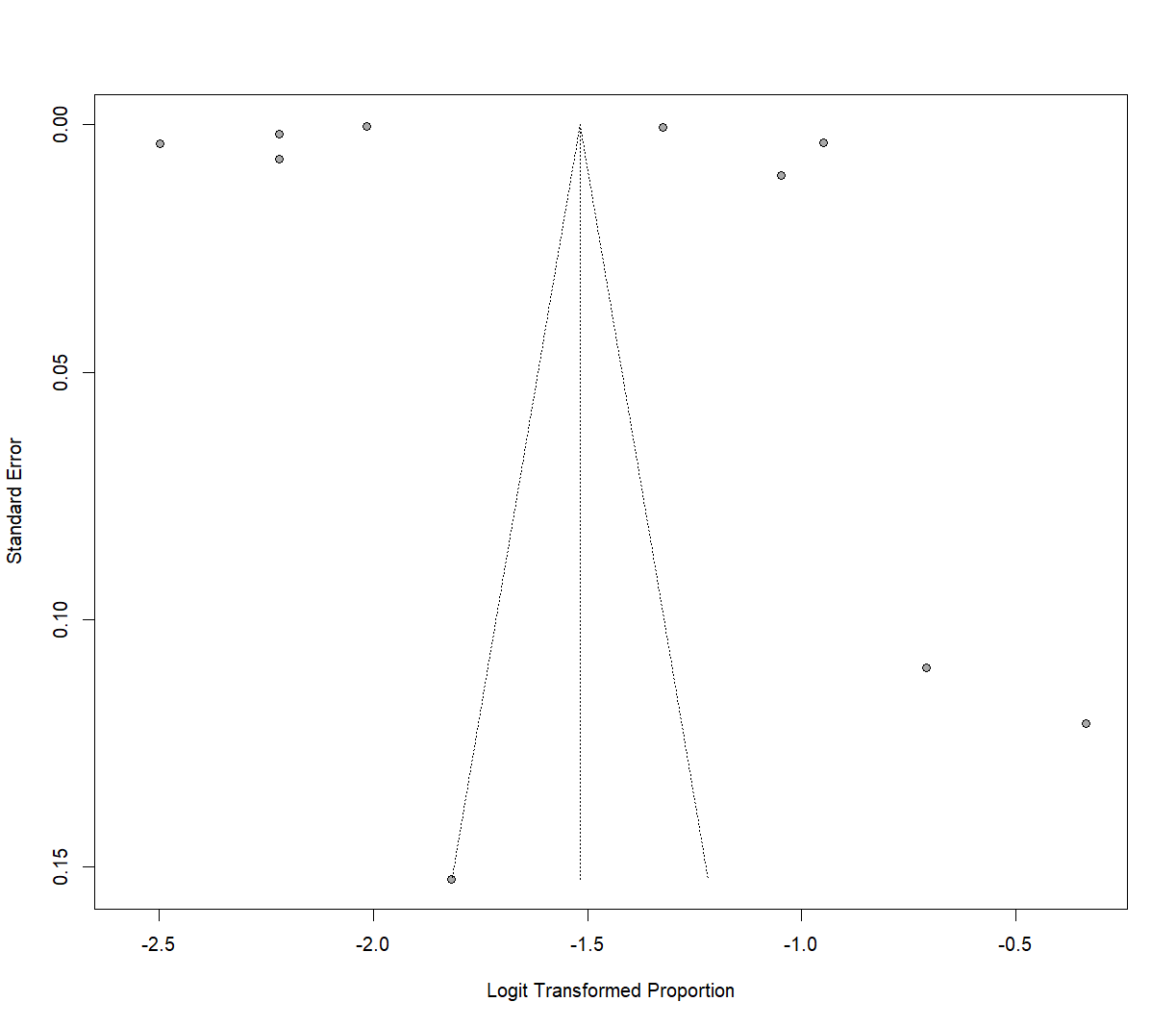


Egger’s test: 0.750

# References

1. Abraham C, Hatoun J, Correa ET, Rabbani N, Vernacchio L. Disparities in the Availability and Acceptance of Nirsevimab in Massachusetts. Pediatrics. 2024;154(1).

2. Aguera M, Soler-Garcia A, Alejandre C, Moussalam-Merino S, Sala-Castellvi P, Pons G, et al. Nirsevimab immunization's real-world effectiveness in preventing severe bronchiolitis: A test-negative case-control study. Pediatr Allergy Immunol. 2024;35(6):e14175.

3. Paediatrics SSO. Supplementum 286: Abstracts of the of the annual meeting of the Swiss Society of Paediatrics (May 22/23, 2025). Swiss medical weekly. 2025;155:4676.

4. Ahmed MM, Wang Z, Joerger T, Michel J, Li Y, Gerber JS. Disparities in Nirsevimab Uptake Across a Pediatric Primary Care Network. Pediatrics. 2025;156(2).

5. Alami A, Perez-Lloret S, Mattison DR. Safety surveillance of respiratory syncytial virus (RSV) vaccine among pregnant individuals: a real-world pharmacovigilance study using the Vaccine Adverse Event Reporting System. BMJ Open. 2025;15(4):e087850.

6. Alejandre C, Penela-Sanchez D, Alsina J, Aguera M, Soler A, Moussalam S, et al. Impact of universal immunization program with monoclonal antibody nirsevimab on reducing the burden of serious bronchiolitis that need pediatric intensive care. Eur J Pediatr. 2024;183(9):3897-904.

7. Andina Martinez D, Claret Teruel G, Gijon Mediavilla M, Camara Otegui A, Banos Lopez L, de Miguel Lavisier B, et al. Nirsevimab and Acute Bronchiolitis Episodes in Pediatric Emergency Departments. Pediatrics. 2024;154(4).

8. Andina Martinez D, Claret Teruel G, Gijon Mediavilla M, Fernandez Mozo E, Camara Otegui A, Banos Lopez L, et al. Nirsevimab and Acute Bronchiolitis Admissions in Infants Under One Year of Age. Pediatr Pulmonol. 2025;60(8):e71249.

9. Ares-Gomez S, Mallah N, Santiago-Perez MI, Pardo-Seco J, Perez-Martinez O, Otero-Barros MT, et al. Effectiveness and impact of universal prophylaxis with nirsevimab in infants against hospitalisation for respiratory syncytial virus in Galicia, Spain: initial results of a population-based longitudinal study. Lancet Infect Dis. 2024;24(8):817-28.

10. Arico MO, Accomando F, Trotta D, Marozzi G, Mariani A, Rossini C, et al. Uneven Implementation of Nirsevimab Prophylaxis Resulted in Non-Uniform Reductions in RSV-Related Hospitalizations in Italy. Infect Dis Rep. 2025;17(5).

11. Assad Z, Romain AS, Aupiais C, Shum M, Schrimpf C, Lorrot M, et al. Nirsevimab and Hospitalization for RSV Bronchiolitis. N Engl J Med. 2024;391(2):144-54.

12. Attaianese F, Trapani S, Agostiniani R, Ambrosino N, Bertolucci G, Biasci P, et al. Effectiveness of a targeted infant RSV immunization strategy (2024-2025): A multicenter matched case-control study in a high-surveillance setting. J Infect. 2025;91(3):106600.

13. Barbas Del Buey JF, Inigo Martinez J, Gutierrez Rodriguez MA, Alonso Garcia M, Sanchez-Gomez A, Lasheras Carbajo MD, et al. The effectiveness of nirsevimab in reducing the burden of disease due to respiratory syncytial virus (RSV) infection over time in the Madrid region (Spain): a prospective population-based cohort study. Front Public Health. 2024;12:1441786.

14. Bermudez-Barrezueta L, Matias Del Pozo V, Marugan-Miguelsanz JM, Lopez EI, Uribe-Reina P, Romero Del Hombrebueno Y, et al. Universal administration of nirsevimab in infants: an analysis of hospitalisations and paediatric intensive care unit admissions for RSV-associated lower respiratory tract infections. Eur J Pediatr. 2025;184(6):345.

15. Blauvelt CA, Zeme M, Natarajan A, Epstein A, Roh ME, Morales A, et al. Respiratory Syncytial Virus Vaccine and Nirsevimab Uptake Among Pregnant People and Their Neonates. JAMA Netw Open. 2025;8(2):e2460735.

16. Bloomfield LE, Pingault NV, Foong RE, French S, Morgan JA, Wadia U, et al. Nirsevimab immunisation of infants and respiratory syncytial virus (RSV)-associated hospitalisations, Western Australia, 2024: a population-based analysis. Med J Aust. 2025;222(11):568-70.

17. Bonnel M, Perrella B, Vaux S, Brunet ML, Jarreau PH, Parat S, et al. Adherence to the nirsevimab immunization campaign: analysis of sociodemographic and medico-economic influences-single-centre prospective cohort study in France. Eur J Pediatr. 2025;184(12):736.

18. Boundy EO, Fast H, Jatlaoui TC, Razzaghi H, Harris L, Nguyen K, et al. Respiratory Syncytial Virus Immunization Coverage Among Infants Through Receipt of Nirsevimab Monoclonal Antibody or Maternal Vaccination - United States, October 2023-March 2024. MMWR Morb Mortal Wkly Rep. 2025;74(31):484-9.

19. Boutin S, Bertrand M, Cohen JF, Zureik M, Chalumeau M, Jabagi MJ. Sociodemographic Characteristics of Infants Receiving Nirsevimab. JAMA Netw Open. 2025;8(4):e254341.

20. Bracaloni S, Esposito E, Scarpaci M, Cosci T, Casini B, Chiovelli F, et al. RSV Disease Burden in Older Adults: An Italian Multiregion Pilot Study of Acute Respiratory Infections in Primary Care Setting, Winter Season 2022-2023. Influenza Other Respir Viruses. 2024;18(12):e70049.

21. Cantais A, Annino N, Thuiller C, Tripodi L, Cesana P, Seigle-Ferrand E, et al. First RSV epidemic with nirsevimab. Older children than previous epidemics, even when hospitalized. J Med Virol. 2024;96(2):e29483.

22. Carazo S, Ouakki M, Skowronski DM, Paquette M, Brousseau N, Talbot D, et al. Nirsevimab effectiveness, number needed to immunize and impact on severe RSV outcomes in preterm, high-risk and healthy-term infants, Quebec, Canada. 2025.

23. Carbajal R, Boelle PY, Pham A, Chazette Y, Schellenberger M, Weil C, et al. Real-world effectiveness of nirsevimab immunisation against bronchiolitis in infants: a case-control study in Paris, France. Lancet Child Adolesc Health. 2024;8(10):730-9.

24. Carcione D, Spencer P, Pettigrew G, Leeb A, Drake-Brockman C, Ford T, Effler P. Active Post-Marketing Safety Surveillance of Nirsevimab Administered to Children in Western Australia, April-July 2024. Pediatr Infect Dis J. 2025.

25. Chauvel C, Horvat C, Javouhey E, Gillet Y, Hassenboehler J, Chakra CNA, et al. Changes in Respiratory Syncytial Virus-Associated Hospitalisations Epidemiology After Nirsevimab Introduction in Lyon, France. Influenza Other Respir Viruses. 2024;18(12):e70054.

26. Cocchi E, Bloise S, Lorefice A, Zannoni S, Pellegrini B, Morlupo FS, et al. Multicentre study on nirsevimab: Bayesian analysis reveals persisting risk for preterm infants. BMJ Paediatr Open. 2025;9(1).

27. Coma E, Martinez-Marcos M, Hermosilla E, Mendioroz J, Rene A, Fina F, et al. Effectiveness of nirsevimab immunoprophylaxis against respiratory syncytial virus-related outcomes in hospital and primary care settings: a retrospective cohort study in infants in Catalonia (Spain). Arch Dis Child. 2024;109(9):736-41.

28. Coma E, Martinez-Marcos M, Hermosilla E, Mendioroz J, Rene A, Fina F, et al. Impact of nirsevimab immunoprophylaxis on respiratory syncytial virus-related outcomes in hospital and primary care after two consecutive seasons: a population-based retrospective cohort study in infants in their second year of life in Catalonia, Spain. Eur J Pediatr. 2025;184(10):616.

29. Coma E, Martinez-Marcos M, Hermosilla E, Mendioroz J, Reñé A, Fina Avilés F, et al. Effectiveness of nirsevimab against RSV-related outcomes: findings of the 2024–2025 campaign in Catalonia align with previous analysis. Archives of Disease in Childhood. 2025;110(12):1024-5.

30. Consolati A, Farinelli M, Serravalle P, Rollandin C, Apprato L, Esposito S, Bongiorno S. Safety and Efficacy of Nirsevimab in a Universal Prevention Program of Respiratory Syncytial Virus Bronchiolitis in Newborns and Infants in the First Year of Life in the Valle d'Aosta Region, Italy, in the 2023-2024 Epidemic Season. Vaccines (Basel). 2024;12(5).

31. Copi T, Kogler MJ, Simonovic Z, Kuder L, Bonca B. 38th Congress of the Fetus as a Patient Society Ljubljana, Slovenia, 14th-16th May 2025. Journal of Perinatal Medicine. 2025;53(s1):s1-s125.

32. Costantino M, Marongiu MB, Corbo MG, Della Corte AM, Frascogna AR, Plantulli A, et al. Efficacy and Safety of Anti-Respiratory Syncytial Virus Monoclonal Antibody Nirsevimab in Neonates: A Real-World Monocentric Study. Vaccines (Basel). 2025;13(8).

33. Creus-Costa A, Andres C, Vila J, Gonzalez-Sanchez A, Lopez N, Ortigosa-Gomez S, et al. Decline in pediatric respiratory syncytial virus hospitalizations following nirsevimab implementation: A multicenter, three-season observational study. Int J Infect Dis. 2025;161:108170.

34. Dahly DL, O’Brien K, Domegan L, O’Leary M, Kelly E, Hanrahan M, et al. Surveillance-based estimation of the impact of introducing a pathfinder programme for nirsevimab immunisation in Ireland on infant hospitalisations due to respiratory syncytial virus in 2024/2025. 2025.

35. Ocana de Sentuary C, Testard C, Lagrée M, Leroy M, Gasnier L, Enes-Dias A, et al. Acceptance and safety of the RSV-preventive treatment of newborns with nirsevimab in the maternity department: a prospective longitudinal cohort study in France. eClinicalMedicine. 2025;79.

36. Scientific Abstracts from The Pediatric Pharmacy Association Annual Meeting April 2025. The Journal of Pediatric Pharmacology and Therapeutics. 2025;30(4):545-60.

37. DeSilva MB, Vazquez-Benitez G, Seburg EM, Henderson MSG, Ehresmann K, Zibley LJ, Palmsten K. Pregnant persons perceptions and uptake of prenatal RSV vaccine - Minnesota, 2023-2024. Vaccine. 2025;54:126958.

38. Dong HV, Pithia N, Chiang C, Halbrook M, Barr M, Yin S, et al. Factors Associated With Newborn Nirsevimab Administration in Los Angeles County During the 2024 Respiratory Syncytial Virus Season. J Pediatric Infect Dis Soc. 2025;14(11).

39. Ernst C, Bejko D, Gaasch L, Hannelas E, Kahn I, Pierron C, et al. Impact of nirsevimab prophylaxis on paediatric respiratory syncytial virus (RSV)-related hospitalisations during the initial 2023/24 season in Luxembourg. Euro Surveill. 2024;29(4).

40. Espeleta-Fox A, Garcia-Salido A, Vallespin-Casas A, Leoz-Gordillo I, Unzueta-Roch JL, De Lama Caro-Paton G, et al. Impact of nirsevimab on admission to a Spanish pediatric intensive care unit because of RSV bronchiolitis: Unicentric observational study from 2017 to 2024. Pediatr Pulmonol. 2024;59(12):3783-6.

41. Estrella-Porter P, Blanco-Calvo C, Lameiras-Azevedo AS, Juaneda J, Fernandez-Martinez S, Gomez-Pajares F, et al. Effectiveness of nirsevimab introduction against respiratory syncytial virus in the Valencian Community: A preliminary assessment. Vaccine. 2024;42(22):126030.

42. Estrella-Porter P, Correcher-Martinez E, Orrico-Sanchez A, Carreras JJ. Post-Marketing Surveillance of Nirsevimab: Safety Profile and Adverse Event Analysis from Spain's 2023-2024 RSV Immunisation Campaign. Vaccines (Basel). 2025;13(6).

43. Ezpeleta G, Navascues A, Viguria N, Herranz-Aguirre M, Juan Belloc SE, Gimeno Ballester J, et al. Effectiveness of Nirsevimab Immunoprophylaxis Administered at Birth to Prevent Infant Hospitalisation for Respiratory Syncytial Virus Infection: A Population-Based Cohort Study. Vaccines (Basel). 2024;12(4).

44. Fafi I, Levy C, Birgy A, Bechet S, Werner A, Batard C, et al. Impact of RSV immunization on the rate of pediatric acute otitis media: a time-series analysis. Clin Infect Dis. 2025.

45. Feitosa DC, Vieira SE. Challenges in the prophylaxis of severe respiratory syncytial virus infections. J Pediatr (Rio J). 2025;101(5):101405.

46. Fortunato F, Prato R, Acquafredda A, Campanozzi A, Carri VD, Francavilla R, et al. Real-World Effectiveness of Nirsevimab in Preventing RSV Hospitalizations: Evidence of Protection in Southern Italian Infants, 2024-2025. J Med Virol. 2025;97(11):e70662.

47. Furgier A, Brehin C, Levy C, Basmaci R, Launay E, Jung C, et al. Effectiveness of nirsevimab against hospitalisation for RSV-bronchiolitis during high RSV-B circulation in the second year of nationwide implementation in France: a test-negative case-control study. Lancet Reg Health Eur. 2025;58:101443.

48. Fusco E, Loiodice M, Romero A, L'Assainato S, Banfi A, Chiaffoni GL, et al. Effectiveness of Nirsevimab in Preventing Respiratory Syncytial Virus-Related Burden: A Test-Negative Case-Control Study in Infants with Bronchiolitis in Lombardy Region, Italy. Pediatr Infect Dis J. 2025.

49. Gabet A, Bertrand M, Jabagi MJ, Olie V, Zureik M. Social and Regional Inequalities in Maternal Respiratory Syncytial Virus Vaccination in France. JAMA Netw Open. 2025;8(9):e2533530.

50. Garcia-Garcia ML, Alonso-Lopez P, Alcolea S, Arroyas M, Pozo F, Casas I, et al. Impact of Nirsevimab on RSV and Non-RSV Severe Respiratory Infections in Hospitalized Infants. Influenza Other Respir Viruses. 2025;19(5):e70105.

51. Garcia Acevedo M, Sanchez Codez MI, Peromingo Matute E, Galan Sanchez F, Delgado Martin B, Quiroga de Castro A, et al. Impact of universal nirsevimab prophylaxis on clinical and ultrasound presentations of lower respiratory tract infections in hospitalized pediatric patients. Eur J Pediatr. 2025;184(10):621.

52. Gentile A, Juarez MDV, Lucion MF, Gregorio G, Lopez O, Fernandez T, et al. Maternal Immunization With RSVpreF Vaccine: Effectiveness in Preventing Respiratory Syncytial Virus-associated Hospitalizations in Infants Under 6 Months in Argentina: Multicenter Case-control Study. Pediatr Infect Dis J. 2025;44(10):988-94.

53. Gentile A, Juarez MDV, Lucion MF, Ensinck G, Lopez O, Melonari P, et al. Respiratory Syncytial Virus Epidemiology in Argentina: From COVID-19 Pandemic to the Maternal Immunization Strategy. Pediatr Infect Dis J. 2025;44(2S):S23-S6.

54. Gonzalez-Bertolin I, Alcolea S, Alonso P, Arroyas M, Fernandez Castiella I, Echavarren I, et al. Second-season Impact of Nirsevimab: Clinical Outcomes of RSV Disease in Patients Immunized During Their First Season. Pediatr Infect Dis J. 2025;44(10):1009-11.

55. Grahic-Mujcinovic O, Smajlovic E, Tabakovic S, Alic A. PALIVIZUMAB IN THE PROPHYLAXIS OF RESPIRATORY SYNCYTIAL VIRUS INFECTIONS. 2024.

56. Gregori-Garcia E, Gasco-Laborda JC, Lluch-Bacas L, Perez-Olaso O, Gomez-Alfaro I, Bellido-Blasco JB. Impact of the passive immunization campaign with nirsevimab in 2023-24 in Castellon. Final results. Enferm Infecc Microbiol Clin (Engl Ed). 2025;43(4):215-8.

57. Guerrero-Del-Cueto F, Lobato-Lopez S, Lozano-Duran D, Sanchez-Duran B, Ramirez-Martin L, Esteban-San-Narciso B, et al. Assessing the Impact of Nirsevimab Immunization on RSV Bronchiolitis Hospital Admissions and Their Severity: A Case-Control Study and Comparison With Pre- and Post-COVID-19 Seasons in a Tertiary Pediatric Hospital. Pediatr Pulmonol. 2025;60(7):e71059.

58. Hammitt LL, Espinoza JS, Keck JW, Hartman RM, Alvarez-Colon G, Burrage A, et al. Nirsevimab is Effective Against Respiratory Syncytial Virus-Associated Hospitalization Among American Indian and Alaska Native Children in Their First and Second RSV Seasons in Alaska and the Southwest United States, 2023-2024. Pediatr Infect Dis J. 2025;44(12):e464-e7.

59. Helwig J, Pham J, Nguyen M, Briars L. Scientific Abstracts from The Pediatric Pharmacy Association Annual Meeting April 2025. The Journal of Pediatric Pharmacology and Therapeutics. 2025;30(4):545-60.

60. Höck M, Borena W, Brunner J, Wechselberger K, Scheiring J, Ralser E, et al. Acceptance and impact of Nirsevimab and the RSVpreF vaccine following implementation in Austria. Frontiers in Public Health. 2025;Volume 13 - 2025.

61. Homo RL, Groberg A, Donahue M, Halverson D, Wooten A, Ponnapakkam A. High Uptake of Respiratory Syncitial Virus Prevention for Neonates in a Military Treatment Facility. J Pediatr. 2024;273:114144.

62. Homo RL, Smith S, Donahue ML, Groberg A, Wooten A, Ponnapakkam A. Demographic Characteristics Associated With Uptake of Neonatal Respiratory Syncytial Virus Prophylaxis. Mil Med. 2025.

63. Hsiao A, Hansen J, Fireman B, Timbol J, Zerbo O, Mari K, et al. Effectiveness of Nirsevimab Against RSV and RSV-Related Events in Infants. Pediatrics. 2025;156(2).

64. Hsieh TYJ, Wei JC, Collier AR. Investigation of maternal outcomes following respiratory syncytial virus vaccination in the third trimester: insights from a real-world United States electronic health records database. Am J Obstet Gynecol. 2025;233(5):e181-e90.

65. Irving SA, Crane B, Weintraub ES, Patel SA, Razzaghi H, Daley MF, et al. Infant Respiratory Syncytial Virus Immunization Coverage in the Vaccine Safety Datalink: 2023-2024. Pediatrics. 2025;155(6).

66. Izquierdo G, Villena R, Cabrera C, Albornoz J, Hueichao N, Guerra C, Torres JP. Safety of timely immunization with nirsevimab in hospitalized preterm infants. Vaccine. 2025;63:127591.

67. Jabagi MJ, Cohen J, Bertrand M, Chalumeau M, Zureik M. Nirsevimab Effectiveness at Preventing RSV-Related Hospitalization in Infants. NEJM Evid. 2025;4(3):EVIDoa2400275.

68. Jesus Perez Martin J, de la Cruz Gomez Moreno M, Sanchez Manresa S, Del Pilar Ros Abellan M, Zornoza-Moreno M. Respiratory syncytial virus immunization with nirsevimab: Acceptance and satisfaction assessment in infants and risk groups in the region of Murcia (Spain). Hum Vaccin Immunother. 2025;21(1):2471700.

69. Jeziorski E, Ouziel A, Cotillon M, Bridonneau C, Bizot E, Basse C, et al. Impact of Nirsevimab on Respiratory Syncytial Virus Bronchiolitis in Hospitalized Infants: A Real-World Study. Pediatr Infect Dis J. 2025;44(4):e124-e6.

70. Jimeno Ruiz S, Pelaez A, Labourt A, Acuna FM, Linares L, Llana Martin I, et al. Evaluating the Effectiveness of Nirsevimab in Reducing Pediatric RSV Hospitalizations in Spain. Vaccines (Basel). 2024;12(10).

71. Joseph NT, Swamy GK. Maternal Immunization and the Implementation Gap-Strengthening Respiratory Syncytial Virus Infrastructure and Preparing for the Future. JAMA Netw Open. 2025;8(2):e2460743.

72. Kalya K, Fontijn J, Bueler-Dill M, Kellenberger D, Ochsenbein-Kolble N, Vonzun L. Societe Suisse de Gynecologie et d'Obstetrique (SGGG) Congress - Abstracts. Gynecol Obstet Invest. 2025;90(Suppl 1):1-107.

73. Kemp M, Capriola A, Schauer S. RSV immunization uptake among infants and pregnant persons - Wisconsin, October 1, 2023-March 31, 2024. Vaccine. 2025;47:126674.

74. Lantigua-Martinez M, Goldberger C, Vertichio R, Kim J, Heo HJ, Roman AS. Respiratory Syncytial Virus Vaccination in Pregnancy and Social Determinants of Health. Am J Perinatol. 2025.

75. Lassoued Y, Levy C, Werner A, Assad Z, Bechet S, Frandji B, et al. Effectiveness of nirsevimab against RSV-bronchiolitis in paediatric ambulatory care: a test-negative case-control study. Lancet Reg Health Eur. 2024;44:101007.

76. Lastrucci V, Pacifici M, Alderotti G, Puglia M, Berti E, Barbati F, et al. The impact of nirsevimab prophylaxis on RSV hospitalizations: a real-world cost-benefit analysis in Tuscany, Italy. Front Public Health. 2025;13:1604331.

77. Lefferts B, Bressler S, Keck JW, Desnoyers C, Hodges E, January G, et al. Nirsevimab Effectiveness Against Medically Attended Respiratory Syncytial Virus Illness and Hospitalization Among Alaska Native Children - Yukon-Kuskokwim Delta Region, Alaska, October 2023-June 2024. MMWR Morb Mortal Wkly Rep. 2024;73(45):1015-21.

78. Lenglart L, Levy C, Basmaci R, Levieux K, Kramer R, De Mari P, et al. Nirsevimab Treatment of RSV Bronchiolitis in Pediatric Emergency Departments. JAMA Netw Open. 2025;8(10):e2540720.

79. Lenglart L, Levy C, Basmaci R, Levieux K, Kramer R, Mari K, et al. Nirsevimab effectiveness on paediatric emergency visits for RSV bronchiolitis: a test-negative design study. Eur J Pediatr. 2025;184(2):171.

80. Levy C, Werner A, Rybak A, Bechet S, Batard C, Hassid F, et al. Early Impact of Nirsevimab on Ambulatory All-Cause Bronchiolitis: A Prospective Multicentric Surveillance Study in France. J Pediatric Infect Dis Soc. 2024;13(7):371-3.

81. Lipsett BJ, Fogel BN, Shedlock KE, Paul IM, Schaefer EW, Gardner RE, et al. Sociodemographic Factors, Intent-Uptake Disparities, and Nirsevimab Availability in Infant RSV Immunoprophylaxis. Pediatr Rep. 2025;17(5).

82. Litman EA, Hsieh TYJ, Modest AM, Clarke K, Dzinoreva M, Perrinez V, et al. Maternal RSVpreF and Infant Nirsevimab Immunizations Uptake During Respiratory Syncytial Virus Season. JAMA Netw Open. 2025;8(2):e2460729.

83. Lopez-Lacort M, Munoz-Quiles C, Mira-Iglesias A, Lopez-Labrador FX, Mengual-Chulia B, Fernandez-Garcia C, et al. Early estimates of nirsevimab immunoprophylaxis effectiveness against hospital admission for respiratory syncytial virus lower respiratory tract infections in infants, Spain, October 2023 to January 2024. Euro Surveill. 2024;29(6).

84. López-Lacort M, Muñoz-Quiles C, Mira-Iglesias A, Xavier López-Labrador F, Garcés-Sánchez M, Escribano-López B, et al. Nirsevimab Effectiveness Against Severe Respiratory Syncytial Virus Infection in the Primary Care Setting. Pediatrics. 2024;155(1).

85. Ma KS, Tsai SY, El Saleeby CM, Kotton CN, Mansbach JM. Nirsevimab decreased the subsequent risk of respiratory syncytial virus infection and wheezing in the 2023-2024 RSV season. Pediatr Res. 2025;98(2):388-90.

86. Mallah N, Pardo-Seco J, Perez-Martinez O, Duran-Parrondo C, Martinon-Torres F, group N-Gs. Full 2023-24 season results of universal prophylaxis with nirsevimab in Galicia, Spain: the NIRSE-GAL study. Lancet Infect Dis. 2025;25(2):e62-e3.

87. Manzanares A, Pardo-Seco J, Rivero-Calle I, Dacosta-Urbieta A, Mallah N, Santiago-Perez MI, et al. Respiratory syncytial virus-related lower respiratory tract infection hospitalizations in infants receiving nirsevimab in Galicia (Spain): the NIRSE-GAL study. Eur J Pediatr. 2025;184(5):321.

88. Manzoni P, Ricco M, Nobili C, Tzialla C, Barera G, Del Barba P, et al. Sustained clinical and epidemiological impact of Respiratory Syncytial Virus (RSV) in young infants exposed to universal immunization with Nirsevimab at birth: An Italian multicenter, retrospective, cohort study 2024/25. J Infect. 2025;91(5):106624.

89. Marouk A, Verrat B, Pontais I, Cojocaru D, Chappuy H, Craiu I, et al. Effectiveness of nirsevimab in reducing hospitalizations in emergency departments due to bronchiolitis among infants under 3 months: a retrospective study. Eur J Pediatr. 2025;184(3):229.

90. Martinon-Torres F, Miras-Carballal S, Duran-Parrondo C. Early lessons from the implementation of universal respiratory syncytial virus prophylaxis in infants with long-acting monoclonal antibodies, Galicia, Spain, September and October 2023. Euro Surveill. 2023;28(49).

91. Mazagatos C, Mendioroz J, Rumayor MB, Gallardo Garcia V, Alvarez Rio V, Cebollada Gracia AD, et al. Estimated Impact of Nirsevimab on the Incidence of Respiratory Syncytial Virus Infections Requiring Hospital Admission in Children < 1 Year, Weeks 40, 2023, to 8, 2024, Spain. Influenza Other Respir Viruses. 2024;18(5):e13294.

92. McLachlan I, Robertson C, Morrison KE, McQueenie R, Hameed SS, Gibbons C, et al. Vaccine effectiveness of the maternal RSVpre-F vaccine against severe disease in infants in Scotland, UK: national population-based case-control and cohort analyses. 2025.

93. Mendez-Echevarria A, Soler-Simon JA, Simon Carro E, Flores Perez P, Jimenez Garcia R, Sanchez-Rico Lucas-Torres B, et al. Early Nirsevimab Administration During Hospitalization for RSV Bronchiolitis may Reduce Disease Severity in Non-immunized infants: A Descriptive Study. Arch Bronconeumol. 2025;61(12):787-90.

94. Molina Gutierrez MA, de Miguel Lavisier B, Ruiz Dominguez JA, Garcia de Oteyza M, Velasco Molina VM, Gutierrez Arroyo A, de Ceano-Vivas M. Impact of nirsevimab immunization on RSV infections attended in the pediatric emergency department: First results in a tertiary hospital in Madrid. Enferm Infecc Microbiol Clin (Engl Ed). 2024;42(7):367-72.

95. Moline HL, Tannis A, Toepfer AP, Williams JV, Boom JA, Englund JA, et al. Early Estimate of Nirsevimab Effectiveness for Prevention of Respiratory Syncytial Virus-Associated Hospitalization Among Infants Entering Their First Respiratory Syncytial Virus Season - New Vaccine Surveillance Network, October 2023-February 2024. MMWR Morb Mortal Wkly Rep. 2024;73(9):209-14.

96. Moline HL, Toepfer AP, Tannis A, Weinberg GA, Staat MA, Halasa NB, et al. Respiratory Syncytial Virus Disease Burden and Nirsevimab Effectiveness in Young Children From 2023-2024. JAMA Pediatrics. 2025;179(2):179-87.

97. Moreno-Perez D, Korobova A, Croche-Santander FB, Cordon-Martinez A, Diaz-Morales O, Martinez-Campos L, et al. Nirsevimab Prophylaxis for Reduction of Respiratory Syncytial Virus Complications in Hospitalised Infants: The Multi-Centre Study During the 2023-2024 Season in Andalusia, Spain (NIRSEGRAND). Vaccines (Basel). 2025;13(2).

98. Moro PL, Gallego R, Scheffey A, Fleming-Dutra KE, Hall E, Zhang B, et al. Administration of the GSK Respiratory Syncytial Virus Vaccine to Pregnant Persons in Error. Obstet Gynecol. 2024;143(5):704-6.

99. Moro PL, Getahun A, Romanson B, Marquez P, Tepper NK, Olson CK, et al. Safety monitoring of Pfizer's Respiratory Syncytial Virus Vaccine in pregnant women in the Vaccine Adverse Event Reporting System (VAERS), 2023-2024, United States. Vaccine. 2025;62:127497.

100. Nguyen D, Lee H, Pavia AT, Nelson RE, Samore M, Chaiyakunapruk N. Optimizing Timing for Respiratory Syncytial Virus Prevention Interventions for Infants. JAMA Netw Open. 2025;8(7):e2522779.

101. Nieddu F, Vignoli M, Ferraro E, Boscia S, Astorino V, Pelosi C, et al. Public health impact of nirsevimab and reduction of RSV hospitalisation in all infants: early real-world data from Tuscany (Italy) in the 2024-25 RSV season. Eur J Pediatr. 2025;184(11):728.

102. Nunez O, Olmedo C, Moreno-Perez D, Lorusso N, Fernandez Martinez S, Pastor Villalba PE, et al. Effectiveness of catch-up and at-birth nirsevimab immunisation against RSV hospital admission in the first year of life: a population-based case-control study, Spain, 2023/24 season. Euro Surveill. 2025;30(5).

103. Paireau J, Durand C, Raimbault S, Cazaubon J, Mortamet G, Viriot D, et al. Nirsevimab Effectiveness Against Cases of Respiratory Syncytial Virus Bronchiolitis Hospitalised in Paediatric Intensive Care Units in France, September 2023-January 2024. Influenza Other Respir Viruses. 2024;18(6):e13311.

104. Viñeta Paramo M, Kiely M, Valiquette L, Muller MP, McGeer A, Isenor JE, et al. Post-Licensure Safety of Nirsevimab from the Canadian National Vaccine Safety (CANVAS) Network. medRxiv. 2025:2025.08.14.25333604.

105. Paris L, Domegan L, O’Leary M, Hanrahan M, McKenna A, Kelly E, et al. Protecting infants from respiratory syncytial virus (RSV) in Ireland: Impact of a national nirsevimab immunisation programme, 2024/2025. 2025.

106. Pastor-Barriuso R, Nunez O, Monge S, Nirsevimab Effectiveness Study C. Infants needed to immunise with nirsevimab to prevent one RSV hospitalisation, Spain, 2023/24 season. Euro Surveill. 2025;30(6).

107. Patton ME, Moline HL, Whitaker M, Tannis A, Pham H, Toepfer AP, et al. Interim Evaluation of Respiratory Syncytial Virus Hospitalization Rates Among Infants and Young Children After Introduction of Respiratory Syncytial Virus Prevention Products - United States, October 2024-February 2025. MMWR Morb Mortal Wkly Rep. 2025;74(16):273-81.

108. Payne AB, Battan-Wraith S, Rowley EAK, Stockwell MS, Tartof SY, Dascomb K, et al. Effectiveness of nirsevimab among infants in their first RSV season in the United States, October 2023-March 2024: a test-negative design analysis. Lancet Reg Health Am. 2025;49:101196.

109. Pelletier JH, Rush SZ, Robinette E, Maholtz DE, Bigham MT, Forbes ML, et al. Nirsevimab Administration and RSV Hospitalization in the 2024-2025 Season. JAMA Netw Open. 2025;8(9):e2533535.

110. Perez Marc G, Vizzotti C, Fell DB, Di Nunzio L, Olszevicki S, Mankiewicz SW, et al. Real-world effectiveness of RSVpreF vaccination during pregnancy against RSV-associated lower respiratory tract disease leading to hospitalisation in infants during the 2024 RSV season in Argentina (BERNI study): a multicentre, retrospective, test-negativ. 2025.

111. Perez Martin JJ, Zornoza Moreno M. Implementation of the first respiratory syncytial (RSV) immunization campaign with nirsevimab in an autonomous community in Spain. Hum Vaccin Immunother. 2024;20(1):2365804.

112. Perramon-Malavez A, de Rioja VL, Coma E, Hermosilla E, Fina F, Martinez-Marcos M, et al. Introduction of nirsevimab in Catalonia, Spain: description of the incidence of bronchiolitis and respiratory syncytial virus in the 2023/2024 season. Eur J Pediatr. 2024;183(12):5181-9.

113. Perramon-Malavez A, Buonsenso D, Morello R, Coma E, Foster S, Leonard P, et al. Real-world impact of nirsevimab immunisation against respiratory disease on emergency department attendances and admissions among infants: a multinational retrospective analysis. Lancet Reg Health Eur. 2025;55:101334.

114. Perramon-Malavez A, Hermosilla E, Coma E, Fina F, Rene A, Martinez-Marcos M, et al. Effectiveness of Nirsevimab Immunoprophylaxis Against Respiratory Syncytial Virus-related Outcomes in Hospital Care Settings: A Seasonal Cohort Study of Infants in Catalonia, Spain. Pediatr Infect Dis J. 2025;44(5):394-8.

115. Petat H, Moisan A, Dubus C, Chabut M, Daligault M, Grosjean J, et al. Shifting Viral Landscapes: The Impact of Respiratory Syncytial Virus Immunization in Infant Bronchiolitis During 2024-2025 Epidemic Season in France. J Med Virol. 2025;97(8):e70515.

116. Puckett L, Kushner LE, Bio L, Cornell S, Wood M, Schwenk HT. Successful Implementation of Nirsevimab and Factors Influencing Uptake in Neonatal Care. Hosp Pediatr. 2025;15(2):99-107.

117. Raguz MJ, Bozic T, Nikse T. Is immunization with palivizumab really effective in high-risk children? J Mother Child. 2022;26(1):87-92.

118. Razai MS, Kalafat E, Prasad S, Lee-Wo C, Heath PT, Khalil A. Perinatal outcomes and uptake of RSV vaccine during pregnancy in South London: a cross-sectional study. BMJ Open. 2025;15(9):e101592.

119. Razzaghi H, Garacci E, Kahn KE, Lindley MC, Jones JM, Stokley S, et al. Maternal Respiratory Syncytial Virus Vaccination and Receipt of Respiratory Syncytial Virus Antibody (Nirsevimab) by Infants Aged <8 Months - United States, April 2024. MMWR Morb Mortal Wkly Rep. 2024;73(38):837-43.

120. Reina J, Iturbe A, Viana-Ramirez J, Sbert G, Carrasco J, Duenas J. Comparative analysis of acute respiratory infections of viral etiology in children under 6 months with and without nirsevimab in the Balearic Islands (2022-2023 and 2023-2024). Enferm Infecc Microbiol Clin (Engl Ed). 2025;43(4):193-6.

121. Remmele J, Helm PC, Li J, Oberhoffer-Fritz R, Bauer UMM, Ewert P. Twins with at least one with CHD and their immunisation status in direct comparison-are both twins complying with the German immunisation recommendations? Cardiovasc Diagn Ther. 2024;14(6):1108-21.

122. Rius-Peris JM, Palomo-Atance E, Muro-Diaz E, Llorente-Ruiz C, Murcia-Clemente L, Alcaraz R. Nirsevimab Immunisation Significantly Reduces Respiratory Syncytial Virus-Associated Bronchiolitis Hospitalisations and Alters Seasonal Patterns. Acta Paediatr. 2025;114(8):1963-76.

123. Schaffer DeRoo S, Hossain T, Chandereng T, Lazerov J. Nirsevimab Uptake in a Pediatric Primary Care Network During the 2023-2024 RSV Season. JAMA Netw Open. 2025;8(7):e2520440.

124. Scruzzi GF, Franchini CG, Giorgetti AC, Fonseca Ingue L, Sarmiento DD, Belfiore SM, et al. Evaluation of the effectiveness of the respiratory syncytial virus vaccine in children under 6 months of age in Cordoba, Argentina. Arch Argent Pediatr. 2025;123(6):e202510741.

125. Shedlock KE, Hicks SD, Gardner RE, Kaye LD, Lipsett BJ, Schaefer EW, et al. Factors Influencing Parental Decisions on Respiratory Syncytial Virus Immunoprophylaxis. J Pediatr Clin Pract. 2025;17:200153.

126. Silva-Afonso RF, Platas-Abenza G, Guerrero-Soler M, Gallardo-Rodriguez P, Gil-Sanchez F, Perez-Paz G, et al. Effectiveness of immunization strategies for preventing severe acute respiratory infection during the 2023/2024 season in a Spanish health department. Enferm Infecc Microbiol Clin (Engl Ed). 2025;43(7):435-43.

127. Somers J, Hansen B, Burger J, Aronoff S, Tuohy B. Newborn RSV immunization rates and reasons compared to family COVID-19 and influenza immunization status. BMC Pediatr. 2025;25(1):555.

128. Son M, Riley LE, Staniczenko AP, Cron J, Yen S, Thomas C, et al. Nonadjuvanted Bivalent Respiratory Syncytial Virus Vaccination and Perinatal Outcomes. JAMA Netw Open. 2024;7(7):e2419268.

129. Steinberg SR, Shah S, Cuevas J, Zia MTK. Maternal Perspectives on Decision-Making for Newborn RSV Prophylaxis With Nirsevimab. J Pediatric Infect Dis Soc. 2025;14(6).

130. Torres JP, Saure D, Goic M, Thraves C, Pacheco J, Burgos J, et al. Effectiveness and impact of nirsevimab in Chile during the first season of a national immunisation strategy against RSV (NIRSE-CL): a retrospective observational study. Lancet Infect Dis. 2025;25(11):1189-98.

131. Touati S, Debs A, Morin L, Jule L, Claude C, Tissieres P, group Ps. Nirsevimab prophylaxis on pediatric intensive care hospitalization for severe acute bronchiolitis: a clinical and economic analysis. Ann Intensive Care. 2025;15(1):56.

132. Vazquez-Lopez P, Rivas-Garcia A, Luaces-Cubells C, Perez-Gonzalez E, Ales-Palmer ML, Cahis-Vela N, et al. Changes in Care in Spanish Pediatric Emergency Departments After the First Immunization With Nirsevimab. Pediatr Emerg Care. 2025;41(5):365-71.

133. Veyrenche N, Toubiana J, Chappuy H, Delacourt C, Bendavid M, Parize P, et al. Success and limitations of the French public health strategy after 2 years of large implementation of respiratory syncytial virus prophylaxis: experience of a tertiary hospital (2018-2025). Int J Infect Dis. 2025;160:108071.

134. Villani A, Antilici L, Musolino AMC, Merola A, Perno CF, Raponi M, Vittucci AC. RSV bronchiolitis: a disease only for those who do not receive prophylaxis. Eur J Pediatr. 2025;184(7):437.

135. Wadia U, Moore HC, Richmond PC, Levy A, Bell L, Pienaar C, et al. Effectiveness of nirsevimab in preventing RSV-hospitalisation among young children in Western Australia 2024. J Infect. 2025;90(4):106466.

136. Wilcox J, Faville E, Wade K, Bianchini G. 2024 ASHP Midyear Clinical Meeting Poster Abstracts. American Journal of Health-System Pharmacy. 2025;82(Supplement_1):S1-S2873.

137. Williams TC, Marlow R, Cunningham S, Drysdale SB, Groves H, Iskander D, et al. Maternal views on RSV vaccination during the first season of implementation in England and Scotland. Lancet Infect Dis. 2025;25(3):e135-e6.

138. Williams TC, Marlow R, Cunningham S, Drysdale SB, Groves HE, Hunt S, et al. Bivalent prefusion F vaccination in pregnancy and respiratory syncytial virus hospitalisation in infants in the UK: results of a multicentre, test-negative, case-control study. Lancet Child Adolesc Health. 2025;9(9):655-62.

139. Xu H, Aparicio C, Wats A, Araujo BL, Pitzer VE, Warren JL, et al. Real-World Effectiveness of Nirsevimab Against Respiratory Syncytial Virus: A Test-Negative Case-Control Study. medRxiv. 2024.

140. Xu H, Aparicio C, Wats A, Araujo BL, Pitzer VE, Warren JL, et al. Estimated Effectiveness of Nirsevimab Against Respiratory Syncytial Virus. JAMA Netw Open. 2025;8(3):e250380.

141. Yan AP, Feng Y, Hassanieh DE, Starr A, Yi M, Schechter-Finkelstein T, et al. Nirsevimab for Prevention of RSV Infections for Immunocompromised Children With Cancer and Stem Cell Transplant Recipients: A Single-Center Experience. Pediatr Blood Cancer. 2025;72(12):e32032.

142. Zhou Y, Hecker K, Engels G, Andres O, Knies K, Krempl C, et al. Changes in RSV-associated lower respiratory tract infections among hospitalized and outpatient children under 2 years in Northern Bavaria after general recommendation of Nirsevimab immunization in 2024. Infection. 2025;53(6):2869-74.

143. Albar Z, Ladikos C, Tisch DJ, Salata R, Saade E. 371. Social Determinants of Vaccine Uptake: The Impact of Race and Vulnerability on Influenza, SARS-CoV-2, and RSV Vaccination Rates. Open Forum Infectious Diseases. 2025;12(Supplement_1).

144. Anonymous. In Brief: New Warning for the RSV Vaccines Arexvy and Abrysvo. The Medical Letter, Inc.; 2025.

145. Blauvelt CA, Natarajan A, Gaw SL. Uptake of the Respiratory Syncytial Virus (RSV) Vaccine and Nirsevimab Among Pregnant Patients and Their Neonates. American Journal of Obstetrics & Gynecology. 2024;231(6):S1309.

146. Jasmine S. Carino, Clifton Cahoon, Natalie Eubanks, Snyder J. Scientific Abstracts from The Pediatric Pharmacy Association Annual Meeting April 2025. The Journal of Pediatric Pharmacology and Therapeutics. 2025;30(4):545-60.

147. Creus-Costa A, Piñana M, Perramon-Malavez A, Andrés C, Rello-Saltor V, Rossich-Verdés R, et al. P-1186. Significant Reduction in Disease Burden and a Shift in Clinical Diagnoses in Children Hospitalized with Respiratory Syncytial Virus (RSV) after Nirsevimab Implementation in Catalonia (Spain). Open Forum Infectious Diseases. 2025;12(Supplement_1).

148. Georgiadis T, Poupouzas GI, Athanasopoulou G, Athanasiou N, Ranellou K, Ioannou M, et al. Characteristics and outcomes of RSV and Influenza hospitalized patients. 2024.

149. Green X, Hage C, Gallo W, Chahoud M, Karaba A, Werbel W. Long-Term Safety of Respiratory Syncytial Virus Vaccination in Solid Organ Transplant Recipients. American Journal of Transplantation. 2025;25(8):S618-S9.

150. Haddad PA, Barmer C, Craig M, Hudson S, Gupta A. Abstract 807: RSV vaccine hesitancy among southern rural veterans with cancer in the Arkansas-Louisiana-Texas (ArkLATX) region: The impact of vaccine fatigue. Cancer Research. 2025;85(8_Supplement_1):807-.

151. Hamid O, Mohammed SS, Awadalla M, Hammad F, Regueiro MD. S1048 Respiratory Syncytial Virus Vaccine is Associated With Better Outcomes in Inflammatory Bowel Disease Patients over 60 Years Old: A US Propensity-Matched Study. American Journal of Gastroenterology. 2024;119(10S):S735-S6.

152. Hsiao A, Hansen J, Timbol J, Fireman B, Zerbo O, Mari K, et al. Effectiveness of nirsevimab in infants against respiratory syncytial virus and related events. Annals of Allergy, Asthma & Immunology. 2024;133(6):S3-S4.

153. Jacobson KB, Watson AJ, Merchant M, Fireman B, Zerbo O, Klein NP. 517. Maternal RSV Vaccination and Infant Nirsevimab Coverage among Infants Born in the 2023-2024 Respiratory Virus Season in a Large Integrated Healthcare System. Open Forum Infectious Diseases. 2025;12(Supplement_1).

154. Jawad M, Tyler J, Barron-Clark A. 2024 ASHP Midyear Clinical Meeting Poster Abstracts. American Journal of Health-System Pharmacy. 2025;82(Supplement_1):S1-S2873.

155. La E, McGuiness C, Singer D, Yasuda M, Chen C. Poster Abstracts - Academy of Managed Care Pharmacy 2024. JMCP. 2024;30(10-c Suppl):S1-S165.

156. Lai X, Ma Y, Zou W, Soudani S, Fang H. EPH206 Public Health Impact of Nirsevimab Against Lower Respiratory Infections Associated with Respiratory Syncytial Virus Among Chinese Infants. Value in Health. 2023;26(12).

157. Loeb LP, Henkes N, Picco MF, Kinnucan JA, Hashash JG, Farraye FA. OUTCOMES OF RESPIRATORY SYNCYTIAL VIRUS INFECTIONS IN PATIENTS WITH INFLAMMATORY BOWEL DISEASE. 2024.

158. Lupton L, Sun X, Lin S, Mikkilineni S, Samuel L, Fernandes J. HPR152 Characterizing Social Determinants of Health in the Population Receiving Respiratory Syncytial Virus Vaccination in a Nationwide Pharmacy Chain in the United States. Value in Health. 2025;28(6):S220-S1.

159. MacDonald SC, Gandhi S, Adimadhyam S, Albert S, Anastasiou OE, Andrade SE, et al. Supplement: Top Scoring Abstracts of the RCOG World Congress 2025, 23-25 June 2025 | ExCeL London. BJOG. 2025;132(Suppl 5):3-199.

160. Mann S, Mason M, Rellosa N, Ravin K. P-633. Implementation and Uptake of Nirsevimab within Nemours Children’s Health Delaware Healthcare System. Open Forum Infectious Diseases. 2025;12(Supplement_1).

161. Martin M, Woods J, Austin V, Shone J, Connell D. THE INCIDENCE AND IMPACT OF INFLUENZA, RSV AND SARS-COV2 ON A SCOTTISH HEALTH BOARD BETWEEN 2022 AND 2024. 2024.

162. Molnar D, La EM, Verelst F, Curran D, Poston S, Van Bellinghen LA, Graham J. EPH45 Assessing the Public Health Impact of the Adjuvanted Respiratory Syncytial Virus Prefusion F Protein Vaccine Among Older Adults in the United States (US). Value in Health. 2023;26(6).

163. Morris DS, Gu T, Li S. 2024 ASHP Midyear Clinical Meeting Poster Abstracts. American Journal of Health-System Pharmacy. 2025;82(Supplement_1):S1-S2873.

164. Rallabhandi SSH, Salman A, Schultz B, Thameem D. Understanding the Current Prevalence of Rsv: Exploring Associations with Immunization, Infection and Hospitalization Trends. Chest. 2024;166(4).

165. Ransohoff H, Frey NA, Moir O, Traub E, Kim A, Yeganeh N, de St. Maurice A. 307. Respiratory Syncytial Virus (RSV) Infections Among Persons Aged 60 Years and Older, by Vaccination Status, reported via Electronic Laboratory Reporting (ELR) – Los Angeles County (LAC), California. Open Forum Infectious Diseases. 2025;12(Supplement_1).

166. Reich PJ, Walsh T, Harford D, Niesen A, Liviskie C, Zeller B, et al. P-1201. Impact of Demographic Factors and Social Determinants of Health on RSV Immunoprophylaxis in Infants. Open Forum Infectious Diseases. 2025;12(Supplement_1).

167. Shelley JT, Walsh T, Gorham L, Madrian E, Neiditz H, Torres AV, et al. P-700. Examining the Knowledge, Availability, Access and Uptake of RSV Immunoprophylasis among RSV Hospitalized Infants. Open Forum Infectious Diseases. 2025;12(Supplement_1).

168. Simeone R, Newhams MM, Zambrano LD, Orzel A, Lindley M, Payne AB, et al. P-2072. COVID-19 and RSV Prevention Products in Pregnancy – Overcoming COVID-19 Network, United States, October 2023–March 2024. Open Forum Infectious Diseases. 2025;12(Supplement_1).

169. Smith-Jeffcoat SE, Benist S, South E, Oyegun EI, Bertumen JB, Grijalva CG, et al. P-62. RSV Immunization Uptake and Barriers to Access Among Eligible Individuals During the First Season of Availability. Open Forum Infectious Diseases. 2025;12(Supplement_1).

170. Tartof SY, Aliabadi N, Goodwin G, Slezak J, Hong V, Ackerson B, et al. P-604. Preliminary real-world Abrysvo vaccine effectiveness (VE) against Respiratory Syncytial Virus (RSV)-related lower respiratory tract disease (LRTD) hospitalizations and emergency department (ED) visits—Kaiser Permanente of Southern California (KPSC), November 2023-April 2024. Open Forum Infectious Diseases. 2025;12(Supplement_1).

171. Torres JP, Saure D, O´Ryan M, Goic M, Thraves C, Trigo N, et al. 169. Universal Immunization Strategy Against Respiratory Syncytial Virus (RSV) Prevention in Chile with Nirsevimab during the 2024 Winter Season: First Southern Hemisphere Nationwide Effectiveness Data. Open Forum Infectious Diseases. 2025;12(Supplement_1).

172. Trivedi M, Phasakda A, Kosaka E, Trivedi N. 1077 Comparative Analysis of Vaccination Rates for COVID-19, Influenza, Pneumonia, and RSV Among Adults Aged 60+ in California. Journal of the American Pharmacists Association. 2025;65(5).

173. Verheust C, Deraedt Q, Bryan P. 574. Early Post-Marketing Safety Surveillance for the Respiratory Syncytial Virus Prefusion F Protein Vaccine (RSVPreF3 OA). Open Forum Infectious Diseases. 2025;12(Supplement_1).

174. Alami A, Perez-Lloret S, Mattison DR. Safety of RSV Vaccine among Pregnant Individuals: A Real-World Pharmacovigilance Study Using Vaccine Adverse Event Reporting System. 2024.

175. Caillault A, Softic L, Bay P, Ly A, Soulier A, Melica G, et al. Molecular Characterization of Respiratory Syncytial Virus Infections in Elderly Patients During the 2023-2024 Season in the Era of Nirsevimab Introduction. J Infect Dis. 2025;232(1):199-202.

176. Domnich A, Orsi A, Lai PL, Massaro E, Trombetta C-S, Pastorino J, et al. Characteristics of the First Italian Older Adults Vaccinated with an Adjuvanted Respiratory Syncytial Virus (RSV) Vaccine. Medicina. 2025;61(1):67.

177. Falsey AR, Branche AR, Peasley M, Cole M, Petrone KK, Obrecht S, et al. Short-Term Immunogenicity of Licensed Subunit RSV Vaccines in Residents of Long-Term Care Facilities (LTCF) Compared to Community-Dwelling Older Adults. J Am Med Dir Assoc. 2024;25(11):105281.

178. Fourati S, Reslan A, Bourret J, Casalegno JS, Rahou Y, Chollet L, et al. Genotypic and phenotypic characterisation of respiratory syncytial virus after nirsevimab breakthrough infections: a large, multicentre, observational, real-world study. Lancet Infect Dis. 2025;25(3):301-11.

179. Gaffney A, Himmelstein DU, McCormick D, Woolhandler S. Respiratory Syncytial Virus (RSV) Vaccine Uptake Among Older Adults: a Population-Based Study of Massachusetts Towns. J Gen Intern Med. 2024;39(15):3096-8.

180. Gratzl S, Farrar KG, Do D, Masters N, Cartwright BMG. Monitoring Report: Respiratory Viruses - February 2025 Data. 2025.

181. Hannaford A, Chamberlin E, Swank Z, Haren SV, Yates B, Kanwal U, et al. Immunogenicity of RSV Vaccination in Thoracic-Organ Transplantation. The Journal of Heart and Lung Transplantation. 2025;44(4).

182. Jasseh I, Manka M, Mendy S, Bajinka O, Makalo L. Feasibility and Acceptability of Respiratory Syncytial Virus Vaccination in Mothers for Infant Protection at Edward Francis Small Teaching Hospital, the Gambia. J Epidemiol Glob Health. 2025;15(1):118.

183. Jasset OJ, Lopez Zapana PA, Bahadir Z, Shook L, Dennis M, Gilbert E, et al. Enhanced placental antibody transfer efficiency with longer interval between maternal respiratory syncytial virus vaccination and birth. Am J Obstet Gynecol. 2025;232(6):554 e1- e15.

184. Kalampokini S, Fountouktsi N, Spilioti M, Finitsis S, Kimiskidis VK. Longitudinal Extensive Transverse Myelitis After Respiratory Syncytial Virus Vaccination With Positive Anti-Recoverin Antibodies. Case Rep Neurol Med. 2025;2025:6597450.

185. Karaba AH, Hage C, Sengsouk I, Balasubramanian P, Segev DL, Tobian A, Werbel WA. Antibody Response to Respiratory Syncytial Virus Vaccination in Immunocompromised Persons. JAMA. 2025;333(5):429-32.

186. Machida M, Inoue S, Furuse Y, Oka E, Ueda Y, Fukushima S, Tabuchi T. Exploring the knowledge and attitude toward respiratory syncytial virus vaccine and associated factors among pregnant women in Japan during the early post-marketing phase. Vaccine. 2025;61:127434.

187. Mestre-Ferrandiz J, Rivero A, Orrico-Sanchez A, Hidalgo A, Abdalla F, Martin I, et al. Evaluation of antibody-based preventive alternatives for respiratory syncytial virus: a novel multi-criteria decision analysis framework and assessment of nirsevimab in Spain. BMC Infect Dis. 2024;24(1):99.

188. Murray A, Clark E, Chu HY, Englund JA. 160. RSV Pre-F IgG levels in pediatric hematopoietic stem cell transplant (HCT) and leukemia/lymphoma patients. Open Forum Infectious Diseases. 2025;12(Supplement_1).

189. Nyiro JU, Bukusi E, Mwaengo D, Nyaguara A, Nyawanda B, Otieno N, et al. Efficiency of transplacental transfer of respiratory syncytial virus (RSV) specific antibodies among pregnant women in Kenya. Wellcome Open Res. 2022;7:43.

190. Seby R, McCormick BJ, Wolf E, Kuhlman J, Jhawar N, Oman SP, et al. Respiratory Syncytial Virus Vaccine Induced Thrombotic Microangiopathy. J Blood Med. 2025;16:331-5.

191. Wei Z, Yu S. Real-world feasibility of co-administration of RSV, COVID-19, and influenza vaccines in older adults: a VAERS-based analysis. Front Pharmacol. 2025;16:1682119.

192. Wetzke M, Lange M, Beinhauer K, Rope E, Borgmann J, Ritter S, et al. Immunization acceptance after broad recommendation for RSV prophylaxis: Results from a cross-sectional study in Germany. Vaccine. 2025;64:127716.

193. Adhikari S, Chapagain RH, Maharjan J, Kunwar K, Pudasaini S, Singh P, et al. Acceptance of New Respiratory Syncytial Virus Vaccine among Pregnant Women in Nepal for Future Routine Immunization: A Descriptive Crosssectional Study. JNMA J Nepal Med Assoc. 2024;62(274):372-7.

194. Bowe A, Barger C, Esterly L, Sylwestrzak G, Dixon S, Poonawalla I. Healthcare utilization and costs following RSV and influenza vaccination in older adults in the United States. Curr Med Res Opin. 2025:1-7.

195. Brault A, Pontais I, Enouf V, Debeuret C, Bloch E, Paireau J, et al. Effect of nirsevimab on hospitalisations for respiratory syncytial virus bronchiolitis in France, 2023-24: a modelling study. Lancet Child Adolesc Health. 2024;8(10):721-9.

196. Du Z, Pandey A, Moghadas SM, Bai Y, Wang L, Matrajt L, et al. Impact of RSVpreF vaccination on reducing the burden of respiratory syncytial virus in infants and older adults. Nat Med. 2025;31(2):647-52.

197. Hansen CL, Lee L, Bents SJ, Perofsky AC, Sun K, Starita LM, et al. Scenario Projections of Respiratory Syncytial Virus Hospitalizations Averted Due to New Immunizations. JAMA Network Open. 2025;8(6):e2514622-e.

198. Maculaitis MC, Hauber B, Beusterien KM, Will O, Kopenhafer L, Law AW, et al. A latent class analysis of factors influencing preferences for infant respiratory syncytial virus (RSV) preventives among pregnant people in the United States. Hum Vaccin Immunother. 2024;20(1):2358566.

199. Marcellusi A, Bini C, Muzii B, Soudani S, Kieffer A, Beuvelet M, et al. Economic and clinical burden associated with respiratory syncytial virus and impact of universal immunization with nirsevimab in Italy. Glob Reg Health Technol Assess. 2025;12:16-28.

200. Sallam M, Kherfan T, Al-Farajat A, Nemrawi L, Atawneh N, Fram R, et al. Attitude to RSV Vaccination Among a Cohort of Pregnant Women in Jordan: A Cross-Sectional Survey Study. Health Sci Rep. 2025;8(1):e70319.

201. Saure D, O'Ryan M, Torres JP, Trigo N, Diaz G, Goic M, et al. Cost-savings and health impact of strategies for prevention of Respiratory Syncytial Virus with nirsevimab in Chile based on the integrated analysis of 2019-2023 national databases: A retrospective study. J Infect Public Health. 2025;18(4):102680.

202. Trubin P, Azar MM, Kotton CN. The respiratory syncytial virus vaccines are here: Implications for solid organ transplantation. Am J Transplant. 2024;24(6):897-904.

203. Trusinska D, Lee B, Ferdous S, Kwok HHY, Gordon B, Gao J, et al. Real-world uptake of nirsevimab, RSV maternal vaccine, and RSV vaccines for older adults: a systematic review and meta-analysis. EClinicalMedicine. 2025;84:103281.

204. Verelst F, Singer D, Graham J, Grace M, La EM, Biundo E. Public health impact of RSV vaccination among adults aged 60 years and older in the United States using real-world evidence from the initial post-introduction season. Expert Rev Vaccines. 2025;24(1):797-806.

205. Wang X, Kong L, Liu X, Wu P, Zhang L, Ding F. Effectiveness of nirsevimab immunization against RSV infection in preterm infants: a systematic review and meta-analysis. Front Immunol. 2025;16:1581970.

206. Biegus J, Szenborn L, Zymlinski R, Zakliczynski M, Reczuch K, Guzik M, et al. The early safety profile of simultaneous vaccination against influenza and Respiratory Syncytial Virus (RSV) in patients with high-risk heart failure. Vaccine. 2024;42(12):2937-40.

207. Domachowske J, Madhi SA, Simoes EAF, Atanasova V, Cabanas F, Furuno K, et al. Safety of Nirsevimab for RSV in Infants with Heart or Lung Disease or Prematurity. N Engl J Med. 2022;386(9):892-4.

208. Domachowske JB, Chang Y, Atanasova V, Cabanas F, Furuno K, Nguyen KA, et al. Safety of Re-dosing Nirsevimab Prior to RSV Season 2 in Children With Heart or Lung Disease. J Pediatric Infect Dis Soc. 2023;12(8):477-80.

209. Ilangovan K, Radley D, Patton M, Shittu E, Lino MM, Goulas C, et al. Integrated Analysis of the Safety Experience in Adults with the Bivalent Respiratory Syncytial Virus Prefusion F Vaccine. Vaccines (Basel). 2025;13(8).

210. Walsh EE, Eiras D, Woodside J, Jiang Q, Patton M, Marc GP, et al. Efficacy, Immunogenicity, and Safety of the Bivalent RSV Prefusion F (RSVpreF) Vaccine in Older Adults Over 2 RSV Seasons. Clin Infect Dis. 2025.

211. Wilson E, Goswami J, Baqui AH, Doreski PA, Perez-Marc G, Zaman K, et al. Efficacy and Safety of an mRNA-Based RSV PreF Vaccine in Older Adults. New England Journal of Medicine. 2023;389(24):2233-44.

212. Andina Martínez D, Barrueco Ramos C, Escalada Pellitero S, Ranera Málaga A, Guerra Díez JL, Gimeno-Hernández Garza V, et al. Unequal impact of respiratory syncytial virus immunization in patients attending Spanish pediatric emergency departments. Emergencias. 2025;37(5):360-6.

213. Matt U, Nitschmann S, Herold S. [Real world data show effectiveness of new respiratory syncytial virus (RSV) vaccines]. Inn Med (Heidelb). 2025;66(4):442-4.

214. Novoa Pizarro JM, Lindemann Tappert BC, Luchsinger Farías VR, Vargas Munita SL. [Prevention of respiratory syncytial virus infection in infants. What has been done and where are we today?]. Andes Pediatr. 2023;94(6):672-80.

215. Rodríguez-Fernández R, González-Martínez F, Ojeda Velázquez I, Rodríguez Díaz M, Capozzi Bucciol MV, González-Sánchez MI, et al. [Nirsevimab effectiveness against hospital admission for respiratory syncytial virus bronchiolitis in infants]. Rev Esp Quimioter. 2024;37(6):498-503.

216. Scruzzi GF, Franchini CG, Giorgetti AC, Fonseca Ingüe L, Sarmiento DD, Belfiore SM, et al. [Safety of respiratory syncytial virus vaccine in pregnant women]. Medicina (B Aires). 2025;85(5):999-1007.

217. Vera-Punzano N, Navascués A, Armendáriz L, Viguria N, Herranz-Aguirre M, García Cenoz M, et al. [Nirsevimab immunization effectiveness against respiratory syncytial virus hospitalization in newborns: two season of use in Navarre, Spain]. An Sist Sanit Navar. 2025;48(2).

218. Institute JB. Critical Appraisal Tools for Use in JBI Systematic Reviews: JBI; 2020 [Available from: <https://jbi.global/critical-appraisal-tools>.

219. Bajema KL, Yan L, Li Y, Argraves S, Rajeevan N, Fox A, et al. Respiratory syncytial virus vaccine effectiveness among US veterans, September, 2023 to March, 2024: a target trial emulation study. Lancet Infect Dis. 2025;25(6):625-33.

220. Birabaharan M, Johns ST, Kaelber DC, Martin TCS, Mehta SR. Atrial Fibrillation after RSV Vaccination Among Older Adults. Clin Infect Dis. 2024.

221. Domnich A, Orsi A, Lai PL, Massaro E, Trombetta CS, Pastorino J, et al. Characteristics of the First Italian Older Adults Vaccinated with an Adjuvanted Respiratory Syncytial Virus (RSV) Vaccine. Medicina (Kaunas). 2025;61(1).

222. Donahue JG, Cocoros NM, Kieke BA, Hanson KE, Weintraub ES, Yih WK, et al. Near real-time surveillance and tree-based data mining to assess the safety of respiratory syncytial virus vaccines in older adults in the vaccine safety datalink. Vaccine. 2025;67:127873.

223. Godonou E-T, Callear AP, Juntila-Raymond CL, Raji D, Smith M, Rumfelt KE, et al. Respiratory syncytial virus (RSV) vaccine effectiveness and antibody correlates of protection among older adults in the Community Vaccine Effectiveness (CoVE) observational study. medRxiv. 2025:2025.03.14.25323981.

224. Hall VG, Alexander AA, Mavandadnejad F, Kern-Smith M, Dang X, Kang R, et al. Safety and immunogenicity of adjuvanted respiratory syncytial virus vaccine in high-risk transplant recipients: an interventional cohort study. Clin Microbiol Infect. 2025.

225. Hause AM, Moro PL, Baggs J, Zhang B, Marquez P, Melgar M, et al. Early Safety Findings Among Persons Aged >/=60 Years Who Received a Respiratory Syncytial Virus Vaccine - United States, May 3, 2023-April 14, 2024. MMWR Morb Mortal Wkly Rep. 2024;73(21):489-94.

226. Havlin J, Skotnicova A, Dvorackova E, Palavandishvili N, Smetanova J, Svorcova M, et al. Respiratory syncytial virus prefusion F3 vaccine in lung transplant recipients elicits CD4+ T cell response in all vaccinees. Am J Transplant. 2025;25(7):1452-60.

227. Kim HS, Lo NC, Boscardin WJ, Guterman EL, Bajema K, Weiser SD, et al. Low Uptake and Disparities in Respiratory Syncytial Virus Vaccination Among US Veterans. Open Forum Infect Dis. 2025;12(11):ofaf434.

228. Levy L, Yahav D, Benzimra M, Bezalel Y, Hoffman T, Shirin N, et al. Neutralizing Antibody Response to the AreXvy Respiratory Syncytial Virus Vaccine in Lung Transplant Recipients: Assessment Against Reference and Seasonal Strains. Vaccines (Basel). 2025;13(4).

229. Lotscher J, Walti CS, Heller S, Hengy Linder F, Drexler B, Gerull S, et al. Respiratory Syncytial Virus Vaccination in Adult Allogeneic Hematopoietic Cell Transplant Recipients. JAMA. 2025;334(16):1478-80.

230. Murphy A, Liu Z, De Souza HG, Chilson EL, Moucka R, Kardel P, et al. Disparities in Respiratory Syncytial Virus Vaccine Uptake in the Medicare Fee-for-Service Population During 2023-2024 Season. J Am Geriatr Soc. 2025.

231. Patrick R, Mahale P, Ackerson BK, Hong V, Shaw S, Kapadia B, et al. Respiratory syncytial virus vaccine uptake among adults aged >/=60 years in a large, integrated healthcare system in Southern California 2023-2024. Vaccine. 2025;53:127033.

232. Redjoul R, Robin C, Softic L, Ourghanlian C, Cabanne L, Beckerich F, et al. Respiratory Syncytial Virus Vaccination in Allogeneic Hematopoietic Stem Cell Transplant Recipients. JAMA Netw Open. 2025;8(9):e2533828.

233. Reses HE, Dubendris H, Haas L, Barbre K, Ananth S, Rowe T, et al. Coverage with Influenza, Respiratory Syncytial Virus, and Updated COVID-19 Vaccines Among Nursing Home Residents - National Healthcare Safety Network, United States, December 2023. MMWR Morb Mortal Wkly Rep. 2023;72(51):1371-6.

234. Reses HE, Segovia G, Dubendris H, Barbre K, Ananth S, Lape-Newman B, et al. Coverage with Influenza, Respiratory Syncytial Virus, and COVID-19 Vaccines Among Nursing Home Residents - National Healthcare Safety Network, United States, November 2024. MMWR Morb Mortal Wkly Rep. 2024;73(46):1052-7.

235. Bao Z, Gao W, Yu X, Chai L, Liu Y. Post-marketing safety monitoring of RSV vaccines: A real-world study based on the Vaccine Adverse Event Reporting System (VAERS). Hum Vaccin Immunother. 2025;21(1):2550857.

236. Geng X, Wang W. Respiratory syncytial virus vaccination among US adults aged >/=60 years. Front Immunol. 2024;15:1427550.

237. La EM, McGuiness CB, Singer D, Yasuda M, Chen CC. RSV vaccination uptake among adults aged 60 years and older in the United States during the 2023-2025 vaccination seasons. Hum Vaccin Immunother. 2025;21(1):2535755.

238. Li J, Zhang Z, Wang M. Post-licensure safety of respiratory syncytial virus vaccines, Vaccine Adverse Event Reporting System, United States, May 2023-December 2024. Prev Med Rep. 2025;56:103150.

239. Morrison R, Sarmiento J, Park J, Lim G, Renda C, Whelan M, et al. A process evaluation of Ontario, Canada's 2023-24 older-adult RSV vaccination program. Hum Vaccin Immunother. 2025;21(1):2550089.

240. Motta M, Callaghan T, Padmanabhan M, Ross J, Gargano LM, Bowman S, Yokum D. Quantifying the prevalence and determinants of respiratory syncytial virus (RSV) vaccine hesitancy in US adults aged 60 or older. Public Health. 2025;238:3-6.

241. Nguyen T, Dawes L, Huang YA, Tay E, Dymock M, O'Moore M, et al. Short term safety profile of respiratory syncytial virus vaccine in adults aged >/= 60 years in Australia. Lancet Reg Health West Pac. 2025;56:101506.

242. Rizzo KR, Yen CJ, Quint J, Hoover C, Schechter R. Sociodemographic disparities in COVID-19 and RSV vaccine uptake among California adults >/=60 years old who received influenza vaccination. Vaccine. 2025;62:127535.

243. Surie D, Yuengling KA, Safdar B, Ginde AA, Peltan ID, Brown SM, et al. Patient- and Community-Level Characteristics Associated With Respiratory Syncytial Virus Vaccination. JAMA Netw Open. 2025;8(4):e252841.

244. Viskupic F, Wiltse DL, Djira G. RSV vaccine uptake among seniors: A path analysis approach. Vaccine. 2025;62:127505.

245. Fry SE, Terebuh P, Kaelber DC, Xu R, Davis PB. Effectiveness and Safety of Respiratory Syncytial Virus Vaccine for US Adults Aged 60 Years or Older. JAMA Netw Open. 2025;8(5):e258322.

246. Payne AB, Watts JA, Mitchell PK, Dascomb K, Irving SA, Klein NP, et al. Respiratory syncytial virus (RSV) vaccine effectiveness against RSV-associated hospitalisations and emergency department encounters among adults aged 60 years and older in the USA, October, 2023, to March, 2024: a test-negative design analysis. Lancet. 2024;404(10462):1547-59.

247. Surie D, Self WH, Zhu Y, Yuengling KA, Johnson CA, Grijalva CG, et al. RSV Vaccine Effectiveness Against Hospitalization Among US Adults 60 Years and Older. JAMA. 2024;332(13):1105-7.

248. Symes R, Whitaker HJ, Ahmad S, Arnold D, Banerjee S, Evans CM, et al. Vaccine effectiveness of a bivalent respiratory syncytial virus (RSV) pre-F vaccine against RSV-associated hospital admission among adults aged 75-79 years in England: a multicentre, test-negative, case-control study. Lancet Infect Dis. 2025.

249. Tartof SY, Aliabadi N, Goodwin G, Slezak J, Hong V, Ackerson B, et al. Estimated Vaccine Effectiveness for Respiratory Syncytial Virus-Related Lower Respiratory Tract Disease. JAMA Netw Open. 2024;7(12):e2450832.

250. Tartof SY, Aliabadi N, Goodwin G, Slezak J, Hong V, Ackerson B, et al. Estimated Vaccine Effectiveness for Respiratory Syncytial Virus-Related Acute Respiratory Illness in Older Adults: Findings From the First Postlicensure Season. Clin Infect Dis. 2025.

251. Hameed SS, Robertson C, Morrison K, McQueenie R, McMenamin J, Ghebrehewet S, Marsh K. Early evidence of RSV vaccination impact on hospitalisation rates of older people in Scotland. Lancet Infect Dis. 2025;25(3):256-8.

252. Mensah AA, Whitaker H, Andrews NJ, Watson CH. Early impact of RSV vaccination in older adults in England. Lancet. 2025;405(10485):1139-40.

253. Lloyd PC, Shah, P.B., Zhang, H.T., Shah, N., Nair, N., Wan, Z., Mu, M., Clarke, T.C., Chen, M., Lin, X., Do, R., Wang, J., Wu, Y., Yoganand Chillarige, Forshee, R.A., Anderson, S.A. Evaluation of Guillain-Barré syndrome following Respiratory Syncytial Virus Vaccination among Medicare Beneficiaries 65 Years and Older. medRxiv (preprint). 2025.
